# Supplementary material for: Topological identification and interpretation for single-cell gene regulation elucidation across multiple platforms using scMGCA
Source: Nat Commun. 2023 Jan 25;14:400. doi: 10.1038/s41467-023-36134-7 (PMC9877026; doi:10.1038/s41467-023-36134-7)
Supplement: Supplementary file 1 — Supplementary Information [file 41467_2023_36134_MOESM1_ESM.pdf]

Supplementary Information "Topological Identification and Interpretation for Single-cell Gene Regulation Elucidation across Multiple Platforms using scMGCA"

Zhuohan Yu[1], Yanchi Su[1], Yifu Lu[1], Yuning Yang[2], Fuzhou Wang[3], Shixiong Zhang[3], Yi Chang[1], Ka-Chun Wong[3*], Xiangtao Li[1*]

[1]School of Artificial Intelligence, Jilin University, Jilin, China

[2]Donnelly Centre for Cellular and Biomolecular Research, University of Toronto, Toronto, ON, Canada

[3]Department of Computer Science, City University of Hong Kong, Hong Kong SAR

DRAFT

Supplementary Note 1: scMGCA provides better performance than other single-cell clustering methods across multiple platforms

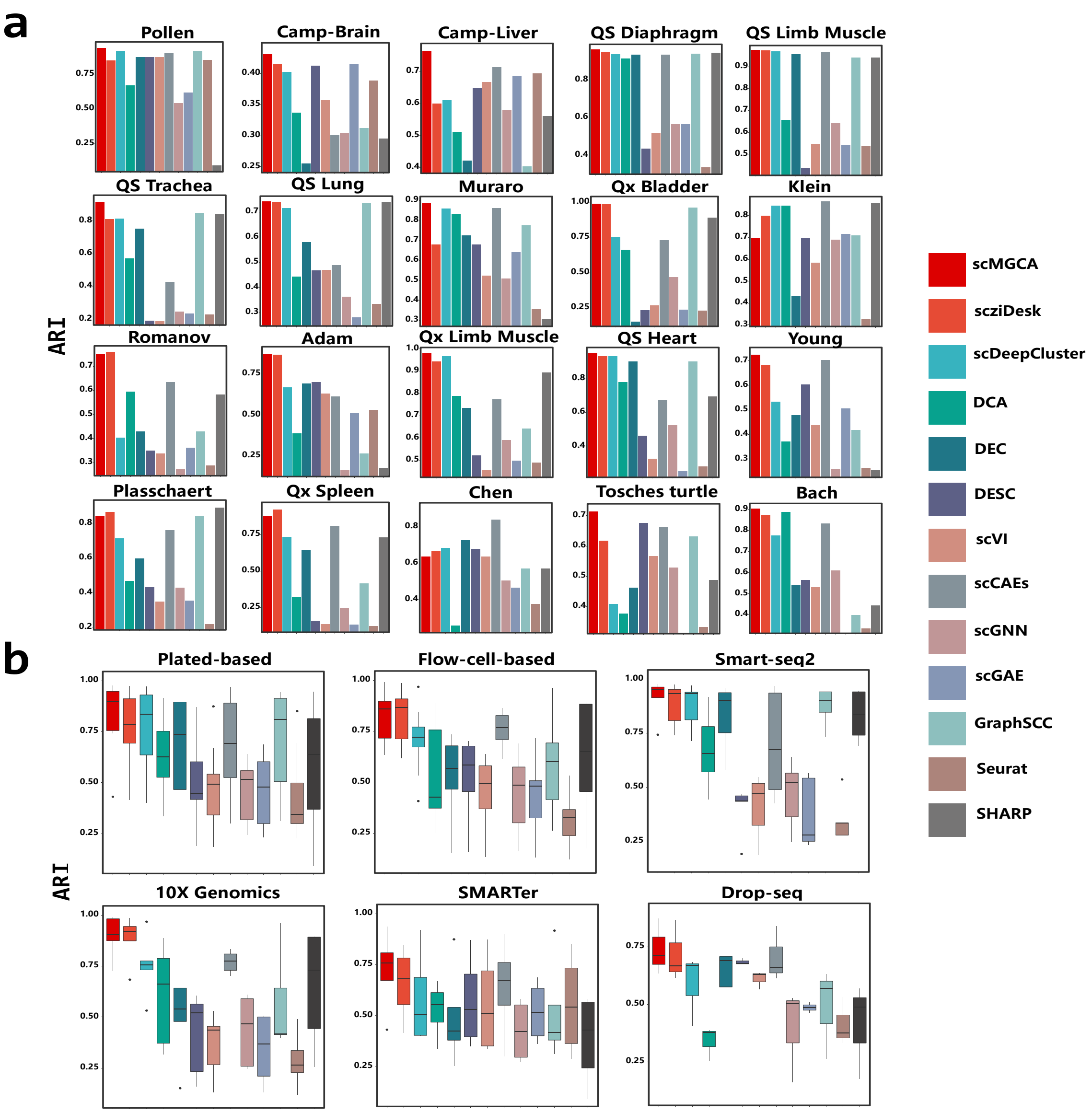

Supplementary Fig. 1. Comparisons of ARI values between scMGCA and 12 single-cell clustering methods on **(a)** 20 real scRNA-seq datasets across multiple platforms and **(b)** on different platforms (n=10,10,5,5,4,3 in each group; center line, median; box limits, upper and lower quartiles; whiskers, 1.5× interquartile range). The blank bar chart in figures generated by scGAE are due to the fact that scGAE cannot run them even with large memory. Source data are provided as a Source Data file.

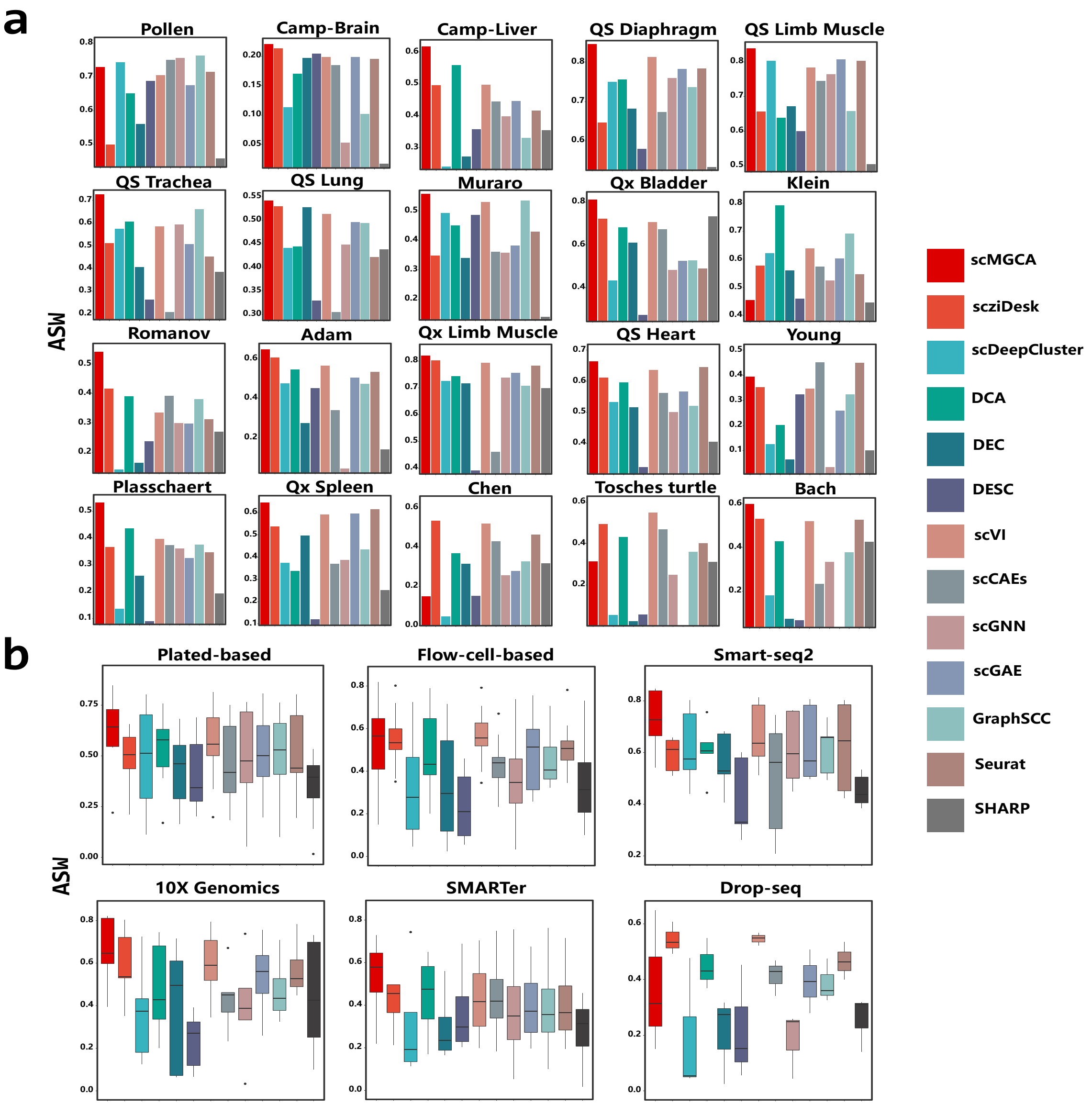

Supplementary Fig. 2. Comparisons of ASW values between scMGCA and 12 single-cell clustering methods on **(a)** 20 real scRNA-seq datasets across multiple platforms and **(b)** on different platforms (n=10,10,5,5,4,3 in each group; center line, median; box limits, upper and lower quartiles; whiskers, 1.5× interquartile range). The blank bar chart in figures generated by scGAE are due to the fact that scGAE cannot run them even with large memory. Source data are provided as a Source Data file.

Supplementary Table 1. Comparison performance of scziDesk with different numbers of highly variable genes measured by NMI, ARI and ASW

Dataset	NMI					ARI					ASW				
	300	500	1000	1500	2000	300	500	1000	1500	2000	300	500	1000	1500	2000
Pollen	0.8982	0.8810	**0.9365**	0.9314	0.8846	0.8776	0.8476	**0.9630**	0.8486	0.8403	0.5317	0.4982	**0.5869**	0.5045	0.4865
Camp-Brain	0.5153	**0.5232**	0.4978	0.4925	0.4964	0.3960	**0.4151**	0.3949	0.3898	0.3678	0.1926	**0.2123**	0.1633	0.1423	0.1678
Camp-Liver	0.7332	0.7343	0.7317	**0.7802**	0.7293	0.5443	0.6005	0.5436	**0.6250**	0.5382	0.4327	**0.4954**	0.4868	0.4849	0.4107
QS Diaphragm	0.8951	0.9210	**0.9362**	0.9239	0.8985	0.9113	0.9517	**0.9741**	0.9714	0.9185	0.7169	0.6466	0.8657	0.8349	**0.8837**
QS Limb Muscle	0.8942	0.9468	**0.9563**	0.9562	0.9535	0.9232	0.9743	0.9745	0.9709	**0.9771**	0.6770	0.6567	**0.8524**	0.8471	0.8504
QS Trachea	0.6439	0.7341	**0.7536**	0.7066	0.7510	0.7662	0.8085	**0.8340**	0.7650	0.8291	0.4428	0.5108	0.5131	0.6253	**0.6954**
QS Lung	**0.8063**	0.7543	0.7580	0.7530	0.7664	**0.7784**	0.7401	0.7397	0.5778	0.5868	0.4642	0.5295	0.4476	0.6097	**0.6172**
Muraro	**0.5233**	0.7349	0.7352	**0.8880**	0.8131	0.3544	0.6784	0.6846	**0.9301**	0.7233	0.0793	0.3478	0.6776	0.3844	**0.6940**
Qx Bladder	0.9101	0.9707	0.9648	0.9653	**0.9910**	0.9411	0.9858	0.9837	0.9843	**0.9968**	0.7318	0.7206	**0.7734**	0.7477	0.7171
Klein	0.7430	0.7777	0.7837	**0.8448**	0.8142	0.7902	0.7984	0.8068	**0.8749**	0.8128	0.5297	0.5775	0.6404	**0.7127**	0.7100
Romanov	0.7201	**0.7274**	0.6660	0.6715	0.6903	0.7566	**0.7603**	0.6299	0.6711	0.7080	0.3233	0.4166	0.4027	**0.4167**	0.4114
Adam	0.8502	**0.8509**	0.8313	0.8429	0.7418	0.8413	**0.8680**	0.8214	0.8400	0.6301	0.5776	**0.6074**	0.5876	0.5843	0.6049
Qx Limb Muscle	0.8338	0.9131	0.9269	0.8587	**0.9263**	0.8746	**0.9441**	0.9376	0.7915	0.9313	0.7295	0.8028	0.7841	0.825	**0.8428**
QS Heart	0.7357	0.8723	0.8941	0.8613	**0.9214**	0.6035	0.9324	0.9557	0.9042	**0.9585**	0.5059	0.6121	**0.7882**	0.7554	0.7208
Young	0.7765	0.7394	0.7343	**0.7976**	0.7366	0.6924	0.6836	0.6940	**0.7361**	0.6884	0.3701	0.3519	0.4139	**0.4880**	0.4432
Plasschaert	0.5748	**0.7899**	0.7867	0.6561	0.6747	0.4111	**0.8634**	0.8591	0.5058	0.5858	0.3628	0.3657	**0.4129**	0.3888	0.4117
Qx Spleen	0.8046	0.8289	0.8212	0.8014	**0.8646**	0.8956	0.9197	0.9291	0.8906	**0.9294**	**0.6909**	0.5368	0.5573	0.6519	0.6481
Chen	0.6511	0.7101	0.7319	0.7117	**0.7538**	0.5738	0.6677	0.7337	0.3132	**0.7498**	0.3168	0.5338	0.5659	**0.5853**	0.5623
Tosches turtle	0.5879	**0.6082**	0.5731	0.5789	0.5379	0.6017	**0.6165**	0.5901	0.6019	0.5517	0.4526	**0.4930**	0.4237	0.4538	0.4103
Bach	0.7953	0.8343	0.8134	0.8269	**0.8645**	0.7406	**0.8738**	0.8293	0.8385	0.8646	0.5406	0.5308	**0.6231**	0.5996	0.5763
Average	0.7446	**0.7926**	0.7916	0.7924	0.7905	0.7137	**0.7965**	0.7939	0.7515	0.7594	0.4834	0.5223	0.5783	0.5821	**0.5932**

Supplementary Table 2. Comparison performance of scziDesk with different hidden layer numbers measured by NMI, ARI and ASW.
scziDesk1 is with ([Input layer]-64-32), scziDesk2 is with ([Input layer]-256-64-32), and scziDesk3 is with ([Input layer]-516-256-64-32)

Dataset	NMI			ARI			ASW		
	scziDesk1	scziDesk2	scziDesk3	scziDesk1	scziDesk2	scziDesk3	scziDesk1	scziDesk2	scziDesk3
Pollen	0.7922	**0.8810**	0.8248	0.6678	**0.8476**	0.7493	**0.5610**	0.4982	0.5453
Camp-Brain	0.5066	**0.5232**	0.5202	0.3916	0.4151	**0.4238**	0.1751	0.2123	**0.2210**
Camp-Liver	**0.7395**	0.7343	0.7329	0.5492	**0.6005**	0.5422	0.4320	**0.4954**	0.2928
QS Diaphragm	0.9100	**0.9210**	0.8846	0.9342	**0.9517**	0.9119	**0.6583**	0.6466	0.6276
QS Limb Muscle	0.9286	**0.9468**	0.9341	0.9435	**0.9743**	0.9495	0.6504	**0.6567**	0.6485
QS Trachea	0.6957	0.7341	**0.8039**	0.7505	**0.8085**	0.7899	**0.6256**	0.5108	0.5554
QS Lung	0.7055	0.7543	**0.7563**	0.4801	**0.7401**	0.5311	0.5359	0.5295	**0.6377**
Muraro	**0.7571**	0.7349	0.7365	0.6528	**0.6784**	0.4959	**0.4390**	0.3478	0.3315
Qx Bladder	**0.9760**	0.9707	0.9537	0.9848	**0.9858**	0.9806	**0.7720**	0.7206	0.4581
Klein	0.7584	**0.7777**	0.7498	0.7774	**0.7984**	0.7146	0.5627	**0.5775**	0.5504
Romanov	0.7165	0.7274	**0.7569**	0.7428	0.7603	**0.7770**	0.4071	0.4166	**0.6893**
Adam	0.8147	**0.8509**	0.8312	0.7989	**0.8680**	0.8253	**0.6199**	0.6074	0.6129
Qx Limb Muscle	0.9102	**0.9131**	0.9125	0.9362	**0.9441**	0.9337	**0.8472**	0.8028	0.8206
QS Heart	0.8086	0.8723	**0.8816**	0.6647	0.9324	**0.9367**	**0.7076**	0.6121	0.6943
Young	0.6957	**0.7394**	0.7216	0.5595	**0.6836**	0.5962	0.3923	0.3519	**0.4177**
Plasschaert	**0.8065**	0.7899	0.6759	**0.8711**	0.8634	0.5343	0.4334	0.3657	**0.4835**
Qx Spleen	0.7509	0.8289	**0.8614**	0.8718	0.9197	**0.9266**	0.5073	**0.5368**	0.5227
Chen	**0.7788**	0.7101	0.7749	0.7821	0.6677	**0.7901**	0.5159	**0.5338**	0.5022
Tosches turtle	0.5941	**0.6082**	0.5821	0.6053	**0.6165**	0.5942	0.4130	**0.4930**	0.4139
Bach	**0.8825**	0.8343	0.7995	**0.8968**	0.8738	0.7262	0.5938	0.5308	**0.6000**
Average	0.7764	**0.7926**	0.7847	0.7431	**0.7965**	0.7365	**0.5425**	0.5223	0.5313

Supplementary Table 3. Comparison performance of scDeepCluster with different numbers of highly variable genes measured by NMI, ARI and ASW

Dataset	NMI					ARI					ASW				
	300	500	1000	1500	2000	300	500	1000	1500	2000	300	500	1000	1500	2000
Pollen	**0.9499**	0.9344	0.9250	0.9401	0.9379	0.9335	0.9185	0.9146	**0.9359**	0.9253	0.7597	0.7437	0.7490	0.7525	**0.7729**
Camp-Brain	0.5263	**0.5365**	0.5293	0.5322	0.5271	0.3919	**0.4021**	0.3973	0.4015	0.3987	0.0124	0.1124	0.1294	0.1268	**0.2435**
Camp-Liver	0.7824	0.7738	0.7848	0.7801	**0.8273**	**0.6187**	0.6102	0.6169	0.6139	0.6731	0.1815	0.241	0.2141	0.3463	**0.4636**
QS Diaphragm	0.9179	0.9191	**0.9572**	0.9562	0.9520	0.9349	0.9392	0.9756	0.979	**0.9798**	0.7883	0.7494	0.8472	0.8296	**0.8297**
QS Limb Muscle	0.8896	0.9505	0.9528	**0.9591**	0.9013	0.9306	0.9701	0.9729	**0.9762**	0.9343	0.7158	**0.8021**	0.7711	0.7505	0.7966
QS Trachea	0.6314	0.7330	**0.7394**	0.5472	0.7240	0.6928	**0.8118**	0.7055	0.5050	0.5716	0.3502	0.5746	**0.6432**	0.2442	0.4655
QS Lung	0.7246	**0.7844**	0.7725	0.7669	0.7489	0.5893	**0.7145**	0.5982	0.6466	0.4993	0.3000	**0.4409**	0.0845	0.3471	0.2089
Muraro	0.6554	0.8292	0.8568	**0.8574**	0.8204	0.5445	0.8589	**0.8742**	0.8727	0.8561	0.3549	0.4931	0.6263	**0.6334**	0.4867
Qx Bladder	0.8021	0.8065	0.7987	**0.8287**	0.7907	0.7617	0.7556	0.7474	**0.8478**	0.7447	0.6361	0.4327	**0.5988**	0.5855	0.4090
Klein	0.6870	**0.8104**	0.6869	0.6920	0.6697	0.6960	**0.8449**	0.5640	0.5598	0.5457	0.4540	**0.6227**	0.5720	0.4650	0.3769
Romanov	0.5594	0.5635	**0.6805**	0.6659	0.6793	0.3988	0.4042	0.6496	0.6255	**0.6521**	0.1194	0.1423	0.4223	**0.4246**	0.3470
Adam	0.7860	0.7669	0.6805	**0.8837**	0.8515	0.7043	0.6689	0.5713	**0.8934**	0.8298	0.4651	0.4773	0.2665	**0.688**	0.6178
Qx Limb Muscle	0.8295	0.9527	**0.9821**	0.9450	0.9369	0.7760	0.9675	**0.9829**	0.9572	0.9515	0.5655	0.7247	**0.7873**	0.7671	0.7628
QS Heart	0.8007	**0.8891**	0.8837	0.8815	0.8753	0.8357	**0.9336**	0.9312	0.9290	0.9291	0.4588	**0.5336**	0.4656	0.5297	0.4355
Young	0.7312	0.7219	**0.7943**	0.7446	0.7373	0.5401	0.5320	**0.6774**	0.6333	0.6141	0.1429	0.1253	**0.4064**	0.2798	0.2164
Plasschaert	0.6589	0.7156	0.6537	**0.7181**	0.6462	0.6008	0.7113	0.5904	**0.7127**	0.6283	0.0727	0.1359	0.0015	0.0176	**0.1746**
Qx Spleen	**0.6869**	0.6698	0.5617	0.5838	0.5765	**0.7685**	0.7316	0.4219	0.5150	0.4699	**0.4117**	0.3736	0.0191	0.0798	0.1523
Chen	0.7201	**0.7597**	0.7430	0.7321	0.7283	0.6174	**0.6842**	0.5254	0.4680	0.4541	**0.0836**	0.0464	0.0835	0.0431	0.0768
Tosches turtle	0.6887	0.6800	0.6824	0.7345	**0.7589**	0.4743	0.4076	0.4221	0.4709	**0.5152**	0.0080	0.0545	0.0411	0.0128	**0.0929**
Bach	0.7078	**0.7868**	0.7631	0.7639	0.7826	0.5754	**0.7749**	0.6579	0.6099	0.7624	0.0613	**0.1811**	0.1764	0.1293	0.1695
Average	0.7368	**0.7792**	0.7714	0.7757	0.7736	0.6693	**0.7321**	0.6898	0.7077	0.6968	0.3471	0.4004	0.3953	0.4026	**0.4049**

Supplementary Table 4. Comparison performance of scDeepCluster with different hidden layer numbers measured by NMI, ARI and ASW. scDeepCluster1 is with ([Input layer]-64-32), scDeepCluster2 is with ([Input layer]-256-64-32), and scDeepCluster3 is with ([Input layer]-516-256-64-32)

Dataset	NMI			ARI			ASW		
	scDeepCluster1	scDeepCluster2	scDeepCluster3	scDeepCluster1	scDeepCluster2	scDeepCluster3	scDeepCluster1	scDeepCluster2	scDeepCluster3
Pollen	0.9341	0.9344	**0.9415**	0.9389	0.9185	**0.9494**	**0.7792**	0.7437	0.7148
Camp-Brain	0.5312	**0.5365**	0.3727	0.4007	**0.4021**	0.2553	0.1614	0.1124	**0.2074**
Camp-Liver	0.7468	**0.7738**	0.7317	**0.7221**	0.6102	0.5389	**0.4353**	0.2410	0.2097
QS Diaphragm	**0.9605**	0.9191	0.8663	**0.9785**	0.9392	0.9398	**0.7768**	0.7494	0.7189
QS Limb Muscle	0.9494	**0.9505**	0.8430	**0.9751**	0.9701	0.9056	0.7933	**0.8021**	0.5682
QS Trachea	**0.8278**	0.7330	0.6885	**0.8633**	0.8118	0.7320	**0.5938**	0.5746	0.5248
QS Lung	0.7601	**0.7844**	0.6390	0.5881	**0.7145**	0.4649	0.4308	**0.4409**	0.1122
Muraro	**0.8312**	0.8292	0.8186	**0.8860**	0.8589	0.8728	**0.5675**	0.4931	0.4487
Qx Bladder	0.7832	0.8065	**0.8079**	0.7375	**0.7556**	0.7554	**0.6263**	0.4327	0.3692
Klein	0.7232	**0.8104**	0.5445	0.7092	**0.8449**	0.4426	0.4825	**0.6227**	0.3434
Romanov	**0.6939**	0.5635	0.5433	**0.6929**	0.4042	0.4091	**0.4278**	0.1423	0.0401
Adam	**0.8285**	0.7669	0.7523	**0.7860**	0.6689	0.6713	**0.4979**	0.4773	0.3538
Qx Limb Muscle	0.9402	**0.9527**	0.8434	0.9509	**0.9675**	0.7585	0.7160	**0.7247**	0.4864
QS Heart	**0.9018**	0.8891	0.8183	**0.9461**	0.9336	0.8727	0.5107	**0.5336**	0.3380
Young	0.7241	0.7219	**0.7728**	0.5658	0.5320	**0.6169**	**0.3704**	0.1253	0.1595
Plasschaert	0.6739	**0.7156**	0.6321	0.6146	**0.7113**	0.5522	0.1289	**0.1359**	0.1054
Qx Spleen	0.6466	**0.6698**	0.5246	0.6721	**0.7316**	0.5129	0.3461	**0.3736**	0.0832
Chen	0.7114	0.7597	**0.7972**	0.4579	0.6842	**0.7648**	0.0315	**0.0464**	0.0431
Tosches turtle	0.6708	0.6800	**0.7017**	0.3745	0.4076	**0.4932**	0.0474	0.0545	**0.0654**
Bach	0.7362	**0.7868**	0.7231	0.6213	**0.7749**	0.6949	**0.2008**	0.1811	0.1548
Average	0.7787	**0.7792**	0.7181	0.7241	**0.7321**	0.6602	**0.4462**	0.4004	0.3024

Supplementary Table 5. Comparison performance of DCA with different numbers of highly variable genes measured by NMI, ARI and ASW

Dataset	NMI					ARI					ASW				
	300	500	1000	1500	2000	300	500	1000	1500	2000	300	500	1000	1500	2000
Pollen	0.7784	0.7773	0.7494	**0.8120**	0.7785	**0.6898**	0.5940	0.5911	0.6680	0.6302	0.4310	0.3317	0.3786	**0.6505**	0.3683
Camp-Brain	0.4018	0.4401	0.4296	**0.4609**	0.4509	0.3394	**0.3839**	0.3704	0.3366	0.3613	0.0194	0.0441	0.0401	**0.1698**	0.0532
Camp-Liver	0.7913	**0.8052**	0.7193	0.6918	0.7020	0.7066	**0.7419**	0.5703	0.5117	0.5774	0.4023	0.4237	0.3960	**0.5589**	0.4232
QS Diaphragm	0.6889	0.8420	0.8919	**0.9174**	0.8941	0.5577	0.8829	0.9411	0.9165	**0.9489**	0.4918	0.6031	0.7252	**0.7557**	0.6646
QS Limb Muscle	0.7354	0.8265	**0.8571**	0.7691	0.8324	0.7575	0.8825	**0.9266**	0.6567	0.7883	0.4742	0.5801	0.5421	**0.6378**	0.5801
QS Trachea	**0.8020**	0.7663	0.7158	0.6688	0.6773	**0.8708**	0.8492	0.6716	0.5707	0.5732	**0.6067**	0.4651	0.4229	0.6059	0.3340
QS Lung	0.6447	**0.6945**	0.6514	0.6400	0.6665	0.5270	**0.5615**	0.5294	0.4429	0.5179	0.3226	0.3818	0.2535	**0.4440**	0.4149
Muraro	**0.5159**	0.5315	0.6414	**0.7865**	0.5361	0.2979	0.3510	0.6100	**0.8300**	0.5916	0.2922	0.2436	0.1818	**0.4515**	0.1679
Qx Bladder	0.6972	0.6638	0.7139	0.6659	**0.7258**	0.7253	0.6853	0.7344	0.6610	**0.7395**	0.2567	0.4611	0.1519	**0.6813**	0.1047
Klein	0.8294	0.8437	0.8512	0.8537	**0.8564**	0.8258	0.8385	0.8418	0.8446	**0.8467**	0.4569	0.4678	0.4998	**0.7920**	0.5209
Romanov	0.5211	0.5476	0.5569	0.5561	**0.6137**	0.4366	0.4495	0.4504	0.5946	**0.6688**	0.1134	0.0461	0.1786	**0.3910**	0.3803
Adam	**0.6380**	0.6195	0.6268	0.5119	0.5977	**0.5137**	0.4855	0.4842	0.3885	0.4349	0.2434	0.2024	0.1913	**0.5485**	0.1352
Qx Limb Muscle	0.7666	0.7799	0.7946	**0.8152**	0.8045	0.7159	0.7268	0.7527	**0.7875**	0.7726	0.4928	0.4823	0.4638	**0.7440**	0.5325
QS Heart	0.5933	0.6578	0.7205	**0.7854**	0.7745	0.4859	0.5266	0.5733	**0.7804**	0.7027	0.1796	0.1649	0.1871	**0.5961**	0.3270
Young	**0.6238**	0.6042	0.5132	0.5339	0.5846	**0.4366**	0.3948	0.3275	0.3716	0.4051	0.0509	0.0503	0.0599	**0.2020**	0.0421
Plasschaert	0.6170	0.6279	0.6375	**0.6466**	0.6126	**0.4910**	0.4842	0.4709	0.4660	0.4139	0.1106	0.0513	0.1096	**0.4371**	0.1165
Qx Spleen	**0.6532**	0.6418	0.5842	0.5398	0.5485	**0.5467**	0.5371	0.5304	0.3173	0.3546	0.3009	0.2996	0.2857	**0.3366**	0.2620
Chen	0.5551	0.5669	**0.6296**	0.5890	0.6043	0.3864	0.5049	**0.5865**	0.2546	0.4446	0.3173	0.3062	0.3087	0.3693	**0.3779**
Tosches turtle	**0.6766**	0.6523	0.6308	0.5947	0.6299	**0.4606**	0.4199	0.4193	0.3767	0.4112	0.3133	0.3143	0.3116	**0.4312**	0.3773
Bach	0.7610	0.7597	0.7415	**0.8372**	0.7483	0.7372	0.7633	0.7522	**0.8871**	0.7474	0.1033	0.1770	0.2006	**0.4291**	0.1566
Average	0.6645	0.6824	0.6828	**0.6838**	0.6819	0.5754	0.6032	**0.6067**	0.5832	0.5965	0.2990	0.3048	0.2944	**0.5116**	0.3170

Supplementary Table 6. Comparison performance of DCA with different hidden layer numbers measured by NMI, ARI and ASW. DCA1 is with ([Input layer]-64-32), DCA2 is with ([Input layer]-256-64-32), and DCA3 is with ([Input layer]-516-256-64-32)

Dataset	NMI			ARI			ASW		
	DCA1	DCA2	DCA3	DCA1	DCA2	DCA3	DCA1	DCA2	DCA3
Pollen	0.8120	**0.8188**	0.8101	0.6680	**0.7007**	0.6552	**0.6505**	0.3941	0.3727
Camp-Brain	**0.4609**	0.4478	0.4606	0.3366	0.3890	**0.4221**	**0.1698**	0.0475	0.0389
Camp-Liver	0.6918	0.7105	**0.7149**	0.5117	**0.5745**	0.5710	**0.5589**	0.4172	0.4908
QS Diaphragm	**0.9174**	0.8969	0.8819	0.9165	**0.9214**	0.9068	**0.7557**	0.6505	0.6908
QS Limb Muscle	**0.7691**	0.7659	0.7376	0.6567	**0.6731**	0.6115	0.6378	0.6216	**0.6427**
QS Trachea	0.6688	0.6874	**0.7606**	0.5707	0.5845	**0.8468**	**0.6059**	0.3429	0.3850
QS Lung	0.6400	0.6684	**0.6793**	0.4429	0.5016	**0.5201**	**0.4440**	0.2670	0.2901
Muraro	**0.7865**	0.5897	0.5688	**0.8300**	0.6759	0.6328	**0.4515**	0.1659	0.0779
Qx Bladder	0.6659	**0.6890**	0.6840	0.6610	**0.7004**	0.6978	**0.6813**	0.5956	0.6149
Klein	**0.8537**	0.8504	0.8528	**0.8446**	0.8404	0.8417	**0.7920**	0.5038	0.4962
Romanov	**0.5561**	0.5342	0.5152	0.5946	**0.5958**	0.5699	**0.3910**	0.3025	0.2936
Adam	0.5119	0.5804	**0.6143**	0.3885	0.4730	**0.4763**	**0.5485**	0.1620	0.1703
Qx Limb Muscle	0.8152	0.8355	**0.8503**	0.7875	0.7974	**0.8079**	**0.7440**	0.4988	0.4744
QS Heart	**0.7854**	0.7742	0.7702	**0.7804**	0.6504	0.6915	**0.5961**	0.2818	0.3681
Young	0.5339	0.5917	**0.5958**	0.3716	**0.4192**	0.3976	**0.2020**	0.0544	0.0498
Plasschaert	0.6466	**0.6836**	0.6815	0.4660	**0.5966**	0.5873	**0.4371**	0.1284	0.0493
Qx Spleen	0.5398	0.5482	**0.5647**	0.3173	0.4403	**0.5167**	**0.3366**	0.2967	0.2680
Chen	0.5890	**0.6019**	0.5803	0.2546	**0.4328**	0.4174	**0.3693**	0.3331	0.3941
Tosches turtle	0.5947	**0.6305**	0.6075	0.3767	**0.4281**	0.3378	**0.4312**	0.3219	0.3301
Bach	**0.8372**	0.7579	0.7346	**0.8871**	0.8555	0.8467	**0.4291**	0.3683	0.3752
Average	**0.6838**	0.6831	0.6833	0.5832	0.6125	**0.6177**	**0.5116**	0.3377	0.3436

Supplementary Table 7. Comparison performance of DEC with different numbers of highly variable genes measured by NMI, ARI and ASW

Dataset	NMI					ARI					ASW				
	300	500	1000	1500	2000	300	500	1000	1500	2000	300	500	1000	1500	2000
Pollen	0.9008	0.8775	**0.9042**	0.8657	0.8743	0.8968	0.8740	**0.9164**	0.8438	0.8743	0.6625	0.5596	**0.6715**	0.5968	0.4473
Camp-Brain	**0.4925**	0.4331	0.4708	0.4618	0.4834	**0.3464**	0.2551	0.3300	0.2932	0.3462	0.1777	**0.1962**	0.1328	0.1351	0.1275
Camp-Liver	**0.7018**	0.6282	0.5899	0.6618	0.6115	**0.5135**	0.4215	0.3679	0.4632	0.3976	**0.5613**	0.2714	0.3494	0.4236	0.0990
QS Diaphragm	0.6963	0.8815	0.8919	**0.9132**	0.8460	0.8015	0.9372	0.9539	**0.9558**	0.9314	0.5042	0.6810	0.6903	0.7303	**0.7801**
QS Limb Muscle	0.8090	**0.9257**	0.6871	0.9097	0.7009	0.8694	**0.9562**	0.5293	0.9469	0.5407	0.5401	0.6716	0.6930	**0.7932**	0.6485
QS Trachea	0.557	0.6843	0.6220	0.6488	**0.7590**	0.6102	0.7518	0.7743	0.7791	**0.8407**	0.3409	0.4064	0.5189	0.5062	**0.6222**
QS Lung	0.6329	0.7285	0.7428	0.7336	**0.7853**	0.4977	0.5793	0.6208	0.6802	**0.7448**	0.4301	0.5280	0.5594	0.5424	**0.5918**
Muraro	0.5842	0.7449	0.8375	0.8424	**0.871**	0.4684	0.7245	0.8895	0.8927	**0.9060**	0.2100	0.3412	0.5984	**0.6419**	0.6013
Qx Bladder	0.3403	0.3610	0.4078	0.4817	**0.5117**	0.1554	0.1515	**0.3643**	0.3433	0.3607	0.5139	**0.6101**	0.5613	0.5454	0.5744
Klein	**0.6932**	0.5397	0.5168	0.5596	0.5346	**0.7501**	0.4327	0.4123	0.4478	0.4546	0.5217	**0.5597**	0.3912	0.4320	0.4938
Romanov	0.5439	**0.5901**	0.585	0.5355	0.5268	0.4153	**0.4289**	0.4128	0.3900	0.3748	0.1422	0.1650	0.3033	0.2501	**0.3406**
Adam	0.6648	**0.747**	0.6359	0.5327	0.5369	0.4888	**0.6903**	0.4614	0.2927	0.2651	**0.2958**	0.2744	0.1945	0.1962	0.2801
Qx Limb Muscle	0.6889	**0.7645**	0.7516	0.5408	0.7218	0.6025	**0.7346**	0.7300	0.3386	0.6079	0.4334	0.7160	**0.7363**	0.3185	0.2833
QS Heart	0.7143	**0.8216**	0.5476	0.7889	0.7991	0.7686	**0.9025**	0.3340	0.8330	0.8716	0.4598	**0.5165**	0.0268	0.2548	0.2680
Young	**0.6476**	0.6420	0.5428	0.6170	0.6163	**0.5170**	0.4785	0.3359	0.4168	0.4144	0.0952	**0.0644**	0.0383	0.0146	0.0711
Plasschaert	0.6372	0.6433	0.7052	0.6808	**0.7500**	0.5883	0.5978	0.7184	0.6721	**0.7254**	0.2260	0.2587	0.2516	**0.2804**	0.2130
Qx Spleen	0.6905	0.6117	**0.7219**	0.6787	0.6384	0.8169	0.6421	**0.8503**	0.8100	0.7552	0.4025	**0.4961**	0.4461	0.3992	0.2678
Chen	0.745	0.7603	0.7697	**0.7877**	0.7564	0.8009	0.7253	0.8426	**0.8496**	0.8254	0.2046	**0.3166**	0.1481	0.2114	0.1016
Tosches turtle	0.6166	0.6617	**0.663**	0.6628	0.6530	0.4334	0.4619	0.3632	**0.4914**	0.4056	0.0406	0.0243	0.0453	0.0234	**0.0409**
Bach	0.6257	0.6259	**0.7355**	0.4299	0.6712	0.6700	0.5393	**0.7639**	0.2563	0.5990	0.1392	0.0726	0.1201	**0.3558**	0.0109
Average	0.6491	**0.6836**	0.6665	0.6667	0.6824	0.6006	**0.6143**	0.5986	0.5998	0.6121	0.3451	**0.3865**	0.3738	0.3826	0.3432

Supplementary Table 8. Comparison performance of DEC with different hidden layer numbers measured by NMI, ARI and ASW. DEC1 is with ([Input layer]-2000-10), DEC2 is with ([Input layer]-500-2000-10), and DEC3 is with ([Input layer]-500-500-2000-10)

Dataset	NMI			ARI			ASW		
	DEC1	DEC2	DEC3	DEC1	DEC2	DEC3	DEC1	DEC2	DEC3
Pollen	0.8595	**0.8941**	0.8775	0.7761	**0.9049**	0.8740	**0.6943**	0.6238	0.5596
Camp-Brain	**0.4620**	0.4609	0.4331	0.3108	**0.3109**	0.2551	0.1835	**0.2099**	0.1962
Camp-Liver	0.6093	**0.6680**	0.6282	0.4074	**0.4338**	0.4215	0.1378	**0.3577**	0.2714
QS Diaphragm	0.8751	**0.8945**	0.8815	0.9076	**0.9383**	0.9372	0.7157	**0.7792**	0.6810
QS Limb Muscle	0.8589	0.8200	**0.9257**	0.8897	0.8906	**0.9562**	**0.7347**	0.6743	0.6716
QS Trachea	0.6390	0.6534	**0.6843**	0.6712	0.7347	**0.7518**	0.3557	**0.4404**	0.4064
QS Lung	0.7237	0.7185	**0.7285**	0.5690	0.5781	**0.5793**	**0.5630**	0.5188	0.5280
Muraro	**0.7469**	0.5786	0.7449	0.7222	0.3436	**0.7245**	0.3845	**0.4001**	0.3412
Qx Bladder	**0.4525**	0.4072	0.3610	**0.2791**	0.2549	0.1515	**0.6217**	0.6125	0.6101
Klein	0.4858	0.4854	**0.5397**	0.3852	0.4059	**0.4327**	0.5034	0.3330	**0.5597**
Romanov	0.5804	0.561	**0.5901**	0.4122	0.422	**0.4289**	**0.2497**	0.1793	0.1650
Adam	0.649	**0.7730**	0.7470	0.4812	**0.7034**	0.6903	0.2770	**0.4708**	0.2744
Qx Limb Muscle	**0.7797**	0.7281	0.7645	**0.805**	0.7139	0.7346	**0.7629**	0.6055	0.7160
QS Heart	0.7882	0.7064	**0.8216**	0.8488	0.7736	**0.9025**	**0.5370**	0.3496	0.5165
Young	0.6241	0.5646	**0.6420**	0.4672	0.4119	**0.4785**	**0.0879**	0.0409	0.0644
Plasschaert	**0.7627**	0.7539	0.6433	**0.7280**	0.7157	0.5978	0.1423	0.2278	**0.2587**
Qx Spleen	0.5885	0.4821	**0.6117**	0.6198	0.5379	**0.6421**	0.3604	0.1728	**0.4961**
Chen	**0.7719**	0.7570	0.7603	**0.7533**	0.7404	0.7253	**0.3464**	0.1590	0.3166
Tosches turtle	**0.6830**	0.6696	0.6617	**0.5026**	0.4764	0.4619	0.1019	**0.1108**	0.0243
Bach	**0.7181**	0.6903	0.6259	0.7265	**0.7478**	0.5393	**0.2668**	0.2619	0.0726
Average	0.6829	0.6633	**0.6836**	0.6131	0.6019	**0.6143**	**0.4013**	0.3764	0.3865

Supplementary Table 9. Comparison performance of DESC with different numbers of highly variable genes measured by NMI, ARI and ASW

Dataset	NMI					ARI					ASW				
	300	500	1000	1500	2000	300	500	1000	1500	2000	300	500	1000	1500	2000
Pollen	**0.9356**	0.9253	0.9209	0.9120	0.9109	0.8821	0.8708	**0.9184**	0.8863	0.8555	0.6740	0.6880	0.7015	**0.7116**	0.6478
Camp-Brain	**0.5388**	0.5307	0.4892	0.4867	0.4820	**0.4309**	0.4125	0.3699	0.3789	0.3582	0.1745	**0.2033**	0.1363	0.1551	0.1185
Camp-Liver	**0.8440**	0.8123	0.8001	0.7843	0.7802	**0.7098**	0.6478	0.5920	0.5732	0.5631	**0.5189**	0.3575	0.4142	0.4262	0.3772
QS Diaphragm	0.7308	0.7180	0.7023	**0.7483**	0.7446	**0.4986**	0.4354	0.3732	0.4983	0.4870	**0.6719**	0.5789	0.6665	0.6464	0.5978
QS Limb Muscle	0.7144	**0.7459**	0.7012	0.7235	0.6883	0.3843	**0.4344**	0.3604	0.3929	0.3753	**0.6373**	0.6002	0.4370	0.4717	0.4523
QS Trachea	0.5801	0.5230	0.5447	0.5473	**0.5825**	**0.2822**	0.1909	0.1907	0.1964	0.2206	0.3013	0.2611	0.2684	0.2824	**0.3301**
QS Lung	0.7416	**0.7504**	0.7134	0.7231	0.7002	0.4756	0.4666	0.3703	0.3374	**0.7002**	0.2130	**0.3297**	0.2855	0.2951	0.1788
Muraro	0.6630	**0.7821**	0.7727	0.7376	0.7114	0.5469	**0.6780**	0.5720	0.4654	0.4112	0.3378	**0.4865**	0.3922	0.3365	0.3059
Qx Bladder	**0.5803**	0.5307	0.5113	0.4956	0.5159	**0.3456**	0.2332	0.2014	0.1732	0.2027	0.3087	0.2715	**0.3627**	0.1512	0.2414
Klein	0.7006	0.6987	0.7859	0.8085	**0.8274**	0.7106	0.6980	0.7719	0.7897	**0.8062**	0.4423	0.4592	0.6083	0.6131	**0.6277**
Romanov	0.5486	0.5791	**0.6048**	0.5728	0.5690	0.3565	0.3497	**0.3854**	0.3213	0.2731	0.2623	0.2377	**0.2861**	0.1937	0.0988
Adam	0.7967	**0.8057**	0.7667	0.7929	0.7750	0.6988	**0.7006**	0.5941	0.6592	0.5814	0.4345	0.4518	0.4608	**0.4611**	0.4515
Qx Limb Muscle	0.7425	**0.7720**	0.7493	0.7417	0.7432	0.4736	**0.5220**	0.4259	0.4096	0.4215	0.4481	0.3909	0.4574	**0.4979**	0.4541
QS Heart	**0.7629**	0.7424	0.6899	0.6695	0.6767	**0.5604**	0.4598	0.3644	0.3176	0.3473	0.2053	**0.3235**	0.1749	0.1330	0.0670
Young	0.7877	0.7858	**0.8003**	0.7796	0.7376	0.6108	0.6036	**0.6678**	0.5732	0.5037	0.3641	0.3239	**0.4162**	0.3804	0.2130
Plasschaert	0.5852	**0.5982**	0.5747	0.5919	0.5793	0.3849	**0.4315**	0.3614	0.3460	0.3351	0.0899	0.0902	**0.1134**	0.0254	0.0343
Qx Spleen	0.4305	0.4439	0.4637	**0.4880**	0.4898	0.1543	0.1585	0.1567	**0.2306**	0.2263	0.0376	0.1195	**0.1318**	0.1245	0.1198
Chen	0.5952	0.7441	**0.7798**	0.7736	0.7431	0.4713	**0.6776**	0.6452	0.6064	0.4813	0.1702	0.1514	**0.2257**	0.1700	0.0176
Tosches turtle	0.6300	0.7013	**0.7357**	0.7122	0.6604	0.4029	0.6758	**0.7137**	0.6985	0.4589	**0.1276**	0.0567	0.0780	0.0030	0.0562
Bach	0.7566	0.7313	0.7900	**0.8151**	0.7568	0.7617	0.5634	0.6750	**0.7907**	0.5731	0.1101	0.0666	0.0931	**0.1153**	0.0075
Average	0.6833	**0.6960**	0.6948	0.6952	0.6837	0.5071	**0.5105**	0.4855	0.4822	0.4591	0.3265	0.3224	**0.3355**	0.3097	0.2699

Supplementary Table 10. Comparison performance of DESC with different hidden layer numbers measured by NMI, ARI and ASW. DESC1 is with ([Input layer]-32-16), DESC2 is with ([Input layer]-128-32-16), and DESC3 is with ([Input layer]-256-128-32-16)

Dataset	NMI			ARI			ASW		
	DESC1	DESC2	DESC3	DESC1	DESC2	DESC3	DESC1	DESC2	DESC3
Pollen	**0.9253**	0.9218	0.9062	0.8708	**0.8951**	0.8607	0.6880	**0.6959**	0.6003
Camp-Brain	0.5307	**0.5326**	0.4979	**0.4125**	0.3704	0.3385	**0.2033**	0.1345	0.1164
Camp-Liver	0.8123	**0.8223**	0.8015	0.6478	**0.6535**	0.5824	0.3575	**0.3889**	0.2445
QS Diaphragm	0.7180	**0.7266**	0.6980	0.4354	**0.4564**	0.4156	**0.5789**	0.4235	0.5589
QS Limb Muscle	**0.7459**	0.7002	0.6724	**0.4344**	0.3546	0.3237	**0.6002**	0.5457	0.4917
QS Trachea	0.5230	**0.5655**	0.5599	0.1909	0.2347	**0.2361**	**0.2611**	0.2576	0.1407
QS Lung	**0.7504**	0.7314	0.7307	**0.4666**	0.3681	0.3860	**0.3297**	0.2700	0.2356
Muraro	**0.7821**	0.7549	0.7588	**0.6780**	0.5554	0.5661	**0.4865**	0.3806	0.4571
Qx Bladder	0.5307	0.5347	**0.5393**	0.2332	0.2512	**0.2879**	0.2715	0.2047	**0.295**
Klein	0.6987	0.7170	**0.7319**	0.6980	0.7168	**0.7474**	0.4592	0.5003	**0.5556**
Romanov	0.5791	0.5874	**0.6022**	0.3497	0.4025	**0.4848**	0.2377	**0.2786**	0.2076
Adam	**0.8057**	0.7979	0.7913	**0.7006**	0.6848	0.6812	0.4518	0.4186	**0.4792**
Qx Limb Muscle	0.7720	0.7906	**0.7950**	0.5220	**0.5744**	0.5346	0.3909	0.4237	**0.4533**
QS Heart	**0.7424**	0.7122	0.7168	**0.4598**	0.4058	0.4326	**0.3235**	0.0179	0.1044
Young	0.7858	**0.8181**	0.8012	0.6036	**0.7111**	0.6933	0.3239	**0.4377**	0.3371
Plasschaert	0.5982	0.5740	**0.6324**	0.4315	0.3851	**0.5157**	0.0902	0.0125	**0.0922**
Qx Spleen	0.4439	0.4836	**0.5213**	0.1585	0.1994	**0.3163**	0.1195	**0.2011**	0.2001
Chen	0.7441	0.7336	**0.7547**	0.6776	0.5741	**0.7363**	**0.1514**	0.0931	0.0539
Tosches turtle	**0.7013**	0.6689	0.6844	**0.6758**	0.4449	0.4812	0.0567	0.0386	**0.0723**
Bach	0.7313	**0.8499**	0.7539	0.5634	**0.8993**	0.5819	0.0666	**0.1971**	0.1422
Average	0.6960	**0.7012**	0.6975	**0.5105**	0.5069	0.5101	**0.3224**	0.2960	0.2919

Supplementary Table 11. Comparison performance of scVI with different numbers of highly variable genes measured by NMI, ARI and ASW

Dataset	NMI					ARI					ASW				
	300	500	1000	1500	2000	300	500	1000	1500	2000	300	500	1000	1500	2000
Pollen	0.9344	**0.9454**	0.9139	0.9393	0.9105	0.8642	**0.8739**	0.8606	0.8696	0.8577	0.7134	0.7044	**0.7695**	0.7672	0.7353
Camp-Brain	0.4964	**0.5082**	0.4785	0.4659	0.5025	0.3538	0.3568	**0.3586**	0.3379	0.3438	0.1878	**0.1982**	0.1790	0.1767	0.1616
Camp-Liver	**0.8553**	0.8161	0.8305	0.8396	0.8496	**0.7172**	0.6676	0.6826	0.6831	0.6886	0.5118	0.4969	0.5641	0.5448	**0.5943**
QS Diaphragm	0.7578	0.7569	0.7575	0.7689	**0.7942**	0.5522	0.5174	0.5086	0.5331	**0.5742**	0.8033	0.8131	0.7982	0.8237	**0.8374**
QS Limb Muscle	0.7325	**0.7754**	0.7715	0.7562	0.7558	0.4384	**0.5474**	0.5249	0.4914	0.5047	0.8020	0.7832	**0.8155**	0.7967	0.8018
QS Trachea	0.5485	0.5438	**0.5592**	0.5435	0.5537	0.1845	0.1862	**0.2044**	0.1962	0.2005	0.5837	0.5856	**0.6518**	0.6305	0.6505
QS Lung	0.7380	**0.7493**	0.7420	0.7489	0.7397	0.4685	**0.4695**	0.4537	0.4652	0.4656	0.4741	**0.5129**	0.5064	0.5093	0.4923
Muraro	0.7411	**0.7615**	0.7428	0.7596	0.7515	0.4964	**0.5243**	0.4748	0.5123	0.4902	0.5178	**0.5307**	0.4040	0.3165	0.5149
Qx Bladder	**0.6133**	0.5501	0.5280	0.5478	0.5138	**0.3803**	0.2671	0.2385	0.2671	0.2172	**0.7281**	0.7066	0.6043	0.6390	0.6833
Klein	0.6906	0.7018	0.7201	**0.7639**	0.7629	0.5558	0.5850	0.5542	**0.6293**	0.6235	0.6203	0.6392	0.7045	0.7111	**0.7464**
Romanov	0.5572	0.5719	0.5671	0.5546	**0.5806**	0.3259	0.3374	0.3572	0.3297	**0.3814**	**0.3681**	0.3358	0.3363	0.2908	0.2872
Adam	**0.7734**	0.7708	0.7556	0.7366	0.7659	**0.6944**	0.6303	0.5793	0.5603	0.6219	0.5198	0.5676	0.5939	0.5792	**0.6036**
Qx Limb Muscle	0.7395	**0.7523**	0.7361	0.7277	0.7393	0.4346	**0.4545**	0.4278	0.4180	0.4276	**0.8034**	0.7930	0.7628	0.7640	0.7855
QS Heart	0.6542	**0.6663**	0.6583	0.6578	0.6449	0.3155	0.3236	0.3213	**0.3252**	0.3048	**0.6481**	0.6358	0.5918	0.5957	0.6376
Young	0.6034	0.6485	0.6664	0.6970	**0.7159**	0.3278	0.4367	0.4716	0.5103	**0.5422**	0.2432	**0.3461**	0.2705	0.2795	0.3331
Plasschaert	0.5480	0.5822	0.5809	**0.5904**	0.5655	0.2721	**0.3474**	0.3296	0.3373	0.3088	0.3608	**0.3974**	0.3692	0.3882	0.3441
Qx Spleen	0.4030	0.4441	**0.4591**	0.4489	0.4545	0.1245	0.1325	0.1503	0.1465	**0.1513**	0.4759	**0.5909**	0.5372	0.4824	0.4775
Chen	0.7876	**0.8042**	0.7048	0.7872	0.7670	0.6347	**0.6369**	0.6122	0.5812	0.5112	0.3754	**0.5212**	0.5168	0.4618	0.4573
Tosches turtle	0.7017	**0.7238**	0.6893	0.6966	0.6923	0.4866	**0.5664**	0.4729	0.5492	0.4926	0.5202	**0.5487**	0.5572	0.5312	0.5237
Bach	0.7340	0.7397	0.7349	**0.7414**	0.7280	**0.5992**	0.5309	0.4956	0.5071	0.4685	0.5133	**0.5190**	0.4551	0.4810	0.4486
Average	0.6805	**0.6906**	0.6798	0.6886	0.6894	0.4613	**0.4696**	0.4539	0.4625	0.4588	0.5385	**0.5613**	0.5494	0.5385	0.5558

Supplementary Table 12. Comparison performance of scVI with different hidden layer numbers measured by NMI, ARI and ASW. scVI1 is with ([Input layer]-128-10), scVI2 is with ([Input layer]-256-128-10), and scVI3 is with ([Input layer]-512-256-128-10)

Dataset	NMI			ARI			ASW		
	scVI1	scVI2	scVI3	scVI1	scVI2	scVI3	scVI1	scVI2	scVI3
Pollen	**0.9454**	0.9015	0.9230	**0.8739**	0.8527	0.8609	0.7044	0.7177	**0.7431**
Camp-Brain	**0.5082**	0.4881	0.4718	**0.3568**	0.3324	0.3118	0.1982	**0.2160**	0.1533
Camp-Liver	0.8161	0.8283	**0.8552**	0.6676	0.6960	**0.6991**	**0.4969**	0.4558	0.4839
QS Diaphragm	**0.7569**	0.7269	0.6519	**0.5174**	0.4683	0.3245	0.8131	**0.8249**	0.7672
QS Limb Muscle	0.7754	0.7484	**0.7764**	**0.5474**	0.4902	0.5236	0.7832	0.7738	**0.7989**
QS Trachea	**0.5438**	0.5370	0.5431	0.1862	0.1680	**0.1870**	0.5856	**0.6564**	0.6423
QS Lung	**0.7493**	0.7424	0.7372	**0.4695**	0.4595	0.4469	0.5129	0.6007	**0.6042**
Muraro	**0.7615**	0.7533	0.7563	**0.5243**	0.5189	0.5204	0.5307	0.4175	**0.5477**
Qx Bladder	0.5501	**0.5738**	0.5150	0.2671	**0.3065**	0.2482	**0.7066**	0.5987	0.6281
Klein	0.7018	**0.7245**	0.6979	**0.5850**	0.5633	0.4809	0.6392	0.6233	**0.6446**
Romanov	**0.5719**	0.5645	0.5528	**0.3374**	0.3094	0.3013	0.3358	0.2884	**0.3461**
Adam	0.7708	**0.7709**	0.7616	0.6303	**0.6613**	0.6528	**0.5676**	0.5558	0.5527
Qx Limb Muscle	0.7523	**0.7565**	0.7461	0.4545	**0.4787**	0.4491	**0.7930**	0.7913	0.7843
QS Heart	0.6663	**0.6916**	0.6652	0.3236	**0.3872**	0.3491	0.6358	**0.6946**	0.6336
Young	**0.6485**	0.6482	0.6451	0.4367	**0.4538**	0.4429	**0.3461**	0.2411	0.2001
Plasschaert	**0.5822**	0.5800	0.5110	**0.3474**	0.3386	0.2273	**0.3974**	0.3493	0.3351
Qx Spleen	0.4441	**0.4463**	0.4272	0.1325	**0.1463**	0.1313	**0.5909**	0.5696	0.5872
Chen	0.8042	**0.8086**	0.8039	0.6369	**0.6545**	0.6534	**0.5212**	0.4988	0.4779
Tosches turtle	0.7238	**0.7816**	0.7627	0.5664	**0.5706**	0.5453	0.5487	0.5291	**0.5648**
Bach	**0.7397**	0.7200	0.7249	**0.5309**	0.4798	0.5186	**0.5190**	0.4934	0.4968
Average	**0.6906**	0.6896	0.6764	**0.4696**	0.4668	0.4437	**0.5613**	0.5448	0.5496

Supplementary Table 13. Comparison performance of scGNN with different numbers of highly variable genes measured by NMI, ARI and ASW

Dataset	NMI					ARI					ASW				
	300	500	1000	1500	2000	300	500	1000	1500	2000	300	500	1000	1500	2000
Pollen	**0.8965**	0.7705	0.8510	0.8828	0.8766	0.8126	0.5427	0.7704	0.8154	**0.8164**	0.7513	**0.7564**	0.6771	0.6847	0.6781
Camp-Brain	0.3638	0.3579	0.3614	**0.3665**	0.3655	0.2945	0.3041	**0.3412**	0.2916	0.2835	0.0296	**0.0528**	0.0249	0.0204	0.0348
Camp-Liver	0.7304	**0.7497**	0.6775	0.7253	0.7206	0.5402	**0.5806**	0.5054	0.5544	0.5500	**0.4163**	0.3985	0.2113	0.1373	0.2809
QS Diaphragm	**0.7660**	0.7608	0.7306	0.7422	0.7231	**0.5857**	0.5646	0.4726	0.4944	0.4522	0.6041	**0.7594**	0.6224	0.5978	0.5317
QS Limb Muscle	0.7297	**0.7726**	0.7008	0.7168	0.6945	0.5360	**0.6399**	0.4451	0.5151	0.4344	0.6421	**0.7636**	0.6841	0.6303	0.6747
QS Trachea	0.5499	0.5699	0.5631	0.5668	**0.5711**	0.2617	0.2459	**0.2813**	0.2608	0.2729	0.5369	0.5944	0.5347	0.5998	**0.6496**
QS Lung	0.6079	**0.6642**	0.6225	0.6501	0.6454	0.3087	**0.3631**	0.3153	0.3629	0.3516	0.4905	0.4487	**0.5122**	0.4515	0.4589
Muraro	**0.6600**	0.6294	0.6124	0.6108	0.5868	0.5047	**0.508**	0.4903	0.4676	0.4067	0.1100	**0.3574**	0.0248	0.0217	0.0239
Qx Bladder	0.5667	**0.6471**	0.5648	0.5686	0.5932	0.3763	**0.4678**	0.3538	0.3529	0.4195	0.4535	**0.482**	0.4781	0.4012	0.4650
Klein	0.5937	**0.7097**	0.6169	0.6633	0.6449	0.4388	**0.6885**	0.4350	0.4955	0.4766	0.2514	**0.5241**	0.3088	0.3201	0.3126
Romanov	**0.4039**	0.3510	0.3934	0.3922	0.3676	**0.3181**	0.2726	0.3088	0.2831	0.2830	0.2627	**0.2998**	0.2721	0.2270	0.2779
Adam	0.2721	0.2731	0.3431	**0.3449**	0.3259	0.1453	0.1608	0.1924	**0.2188**	0.2047	0.0302	0.0444	0.0344	0.0195	**0.0494**
Qx Limb Muscle	0.7346	0.7457	0.7170	0.7124	**0.7547**	0.6088	0.5899	0.5267	0.5119	**0.6094**	0.5330	**0.7380**	0.6021	0.5890	0.5437
QS Heart	0.6059	0.6540	**0.7012**	0.6859	0.6804	0.4876	0.5222	**0.5749**	0.5361	0.5322	0.5123	0.5003	0.5381	0.5079	**0.5417**
Young	0.3798	**0.4143**	0.3495	0.3807	0.3925	0.2533	**0.2588**	0.2158	0.2345	0.2471	**0.0419**	0.0332	0.0293	0.0303	0.0325
Plasschaert	0.5824	**0.5856**	0.5663	0.5661	0.5626	0.3824	**0.4272**	0.3506	0.3501	0.3388	0.3219	**0.3618**	0.3047	0.3027	0.3046
Qx Spleen	0.4186	0.4621	0.4012	0.4238	**0.4660**	0.2355	0.2464	0.2269	0.2324	**0.2523**	0.3541	**0.3868**	0.3579	0.3653	0.3704
Chen	0.6139	**0.6854**	0.6205	0.6623	0.6252	0.5310	0.5035	0.5573	**0.5809**	0.5441	0.2846	0.2576	**0.2891**	0.2632	0.2639
Tosches turtle	0.5161	**0.5427**	0.5007	0.4936	0.5160	0.5070	**0.5279**	0.3002	0.4147	0.4250	0.1319	**0.2485**	0.1516	0.2031	0.1673
Bach	0.7377	**0.7430**	0.7286	0.6950	0.6887	0.5877	**0.6089**	0.5732	0.5153	0.5132	0.2938	**0.3334**	0.2563	0.2457	0.2469
Average	0.5865	**0.6044**	0.5811	0.5925	0.5901	0.4358	**0.4512**	0.4119	0.4244	0.4207	0.3526	**0.4171**	0.3457	0.3309	0.3454

Supplementary Table 14. Comparison performance of scGNN with different hidden layer numbers measured by NMI, ARI and ASW. scGNN1 is with ([Input layer]-32-16), scGNN2 is with ([Input layer]-128-32-16), and scGNN3 is with ([Input layer]-256-128-32-16)

Dataset	NMI			ARI			ASW		
	scGNN1	scGNN2	scGNN3	scGNN1	scGNN2	scGNN3	scGNN1	scGNN2	scGNN3
Pollen	0.7705	**0.7946**	0.7524	0.5427	**0.7052**	0.5710	0.7564	**0.7683**	0.7542
Camp-Brain	0.3579	0.3308	**0.3681**	0.3041	0.2873	**0.3162**	**0.0528**	0.0476	0.0517
Camp-Liver	**0.7497**	0.7245	0.7182	**0.5806**	0.5650	0.5344	**0.3985**	0.3868	0.3927
QS Diaphragm	**0.7608**	0.7376	0.7388	**0.5646**	0.5465	0.5395	**0.7594**	0.7329	0.7354
QS Limb Muscle	**0.7726**	0.7655	0.7503	**0.6399**	0.6175	0.5580	**0.7636**	0.7417	0.6933
QS Trachea	0.5699	0.5781	**0.5866**	0.2459	0.2881	**0.2973**	**0.5944**	0.5822	0.5764
QS Lung	**0.6642**	0.6582	0.6182	0.3631	**0.3653**	0.3170	0.4487	**0.4581**	0.4526
Muraro	**0.6294**	0.6104	0.6286	**0.508**	0.5035	0.5061	0.3574	**0.3620**	0.3548
Qx Bladder	0.6471	0.6264	**0.6505**	**0.4678**	0.4568	0.4675	**0.4820**	0.4331	0.4785
Klein	0.7097	**0.7226**	0.7120	0.6885	**0.6912**	0.6843	0.5241	0.5239	**0.5547**
Romanov	**0.3510**	0.306	0.3484	0.2726	0.2915	**0.303**	**0.2998**	0.2865	0.2744
Adam	**0.2731**	0.2703	0.2566	**0.1608**	0.1386	0.1319	**0.0444**	0.0296	0.0458
Qx Limb Muscle	0.7457	**0.7510**	0.7437	0.5899	**0.6174**	0.5942	**0.7380**	0.7028	0.6517
QS Heart	0.6540	**0.6853**	0.6801	0.5222	0.5211	**0.5295**	0.5003	0.5283	**0.5560**
Young	**0.4143**	0.3468	0.3671	**0.2588**	0.2177	0.2496	0.0332	0.0257	**0.0345**
Plasschaert	**0.5856**	0.5843	0.5826	**0.4272**	0.4163	0.3854	**0.3618**	0.3603	0.3244
Qx Spleen	0.4621	**0.4686**	0.4683	0.2464	**0.2571**	0.2555	**0.3868**	0.3749	0.3758
Chen	0.6854	**0.6937**	0.6825	0.5035	**0.5365**	0.5026	0.2576	**0.2879**	0.2315
Tosches turtle	**0.5427**	0.5348	0.5316	**0.5279**	0.5077	0.5023	**0.2485**	0.1916	0.2258
Bach	**0.7430**	0.7409	0.7365	**0.6089**	0.5933	0.5821	0.3334	**0.3348**	0.2974
Average	**0.6044**	0.5965	0.5961	0.4512	**0.4562**	0.4414	**0.4171**	0.4080	0.4031

Supplementary Table 15. Comparison performance of scGAE with different numbers of highly variable genes measured by NMI, ARI and ASW

Dataset	NMI					ARI					ASW				
	300	500	1000	1500	2000	300	500	1000	1500	2000	300	500	1000	1500	2000
Pollen	0.8073	0.8133	**0.8327**	0.8321	0.8152	0.6151	0.6158	0.6613	0.6598	0.6572	0.6080	**0.6752**	0.5712	0.5903	0.6438
Camp-Brain	0.5181	**0.5201**	0.5086	0.4736	0.4726	0.4047	**0.4152**	0.3866	0.3602	0.3657	**0.2299**	0.1982	0.1112	0.1283	0.2040
Camp-Liver	**0.8420**	0.8109	0.8181	0.8142	0.7945	**0.7683**	0.6872	0.684	0.6572	0.6036	**0.6370**	0.4455	0.5596	0.4944	0.5097
QS Diaphragm	0.7262	0.7351	0.7480	0.7375	**0.7609**	0.6172	0.5638	0.6302	0.6273	**0.6467**	0.7009	0.7829	0.7681	**0.8165**	0.7766
QS Limb Muscle	0.6607	**0.7398**	0.7198	0.6551	0.7187	0.4543	**0.5419**	0.5349	0.5260	0.4355	0.5811	0.8067	**0.8074**	0.7240	0.7844
QS Trachea	0.4693	0.5148	0.4973	**0.5239**	0.4880	0.1720	0.2335	0.1568	**0.2399**	0.1337	0.3654	0.5073	0.4531	**0.5227**	0.4432
QS Lung	0.6970	0.6766	0.6783	**0.6989**	0.6901	**0.4838**	0.2797	0.2902	0.3113	0.3130	0.4436	0.4963	0.4522	**0.4982**	0.3519
Muraro	0.5369	**0.7619**	0.7096	0.6885	0.6877	0.3281	**0.6413**	0.4525	0.3645	0.4059	0.2678	0.3820	**0.5307**	0.4843	0.4586
Qx Bladder	0.4842	0.5006	0.4948	**0.5176**	0.4694	0.2034	0.2369	0.1730	**0.2877**	0.1476	**0.6757**	0.5240	0.6258	0.6151	0.4296
Klein	0.5775	0.6848	0.7196	0.7585	**0.7919**	0.5694	0.7148	0.6755	0.7399	**0.7750**	0.5167	0.6035	0.6199	0.6472	**0.6778**
Romanov	0.4501	**0.5205**	0.5368	0.5042	0.5641	0.1938	**0.3609**	0.2602	0.3584	0.2787	0.2246	0.2974	0.3114	0.3553	**0.3573**
Adam	0.6302	0.6784	0.6450	**0.7011**	0.6929	0.3413	0.5090	0.3422	**0.5381**	0.5336	0.4190	**0.5070**	0.4611	0.4448	0.4334
Qx Limb Muscle	0.6744	**0.7569**	0.7562	0.7363	0.7561	0.4818	**0.4983**	0.4944	0.4884	0.4886	0.6004	**0.7551**	0.7303	0.7517	0.7205
QS Heart	0.5177	**0.6039**	0.5939	0.5877	0.5996	0.1704	**0.2497**	0.2185	0.2141	0.2355	0.4789	**0.5677**	0.5293	0.5466	0.4487
Young	0.6822	0.6536	0.6763	**0.7025**	0.6364	0.5176	0.5066	0.5121	**0.5220**	0.2966	0.2339	**0.2597**	0.2587	0.2307	0.1832
Plasschaert	0.3954	0.5563	0.5296	**0.6068**	0.5590	0.1098	0.3540	0.2350	**0.3778**	0.2818	0.2099	0.3252	0.2996	0.2731	**0.3627**
Qx Spleen	0.4040	0.4287	**0.458**	0.4306	0.4350	0.1447	0.1314	**0.2100**	0.1686	0.1700	0.5371	**0.5957**	0.5651	0.4334	0.4002
Chen	0.6386	**0.6703**	0.6625	0.6360	0.6658	0.3962	0.4631	**0.6762**	0.4293	0.6035	0.1414	0.2800	0.3234	**0.3744**	0.3671
Tosches turtle	-	-	-	-	-	-	-	-	-	-	-	-	-	-	-
Bach	-	-	-	-	-	-	-	-	-	-	-	-	-	-	-
Average	0.5951	**0.6459**	0.6436	0.6447	0.6443	0.3873	**0.4446**	0.4219	0.4373	0.4096	0.4373	**0.5005**	0.4988	0.4962	0.4752

The '-' is due to the fact that scGAE cannot run them even with large memory.

Supplementary Table 16. Comparison performance of scGAE with different hidden layer numbers measured by NMI, ARI and ASW. scGAE1 is with ([Input layer]-128-10), scGAE2 is with ([Input layer]-256-128-10), and scGAE3 is with ([Input layer]-512-256-128-10)

Dataset	NMI			ARI			ASW		
	scGAE1	scGAE2	scGAE3	scGAE1	scGAE2	scGAE3	scGAE1	scGAE2	scGAE3
Pollen	0.8133	**0.8351**	0.7866	0.6158	**0.6979**	0.6286	**0.6752**	0.4677	0.6157
Camp-Brain	**0.5201**	0.5103	0.4722	0.4152	**0.4600**	0.3527	**0.1982**	0.1422	0.1358
Camp-Liver	0.8109	**0.8184**	0.7960	0.6872	**0.6877**	0.6266	**0.4455**	0.4056	0.2328
QS Diaphragm	0.7351	0.7288	**0.7587**	0.5638	0.6584	**0.6736**	**0.7829**	0.6975	0.6872
QS Limb Muscle	0.7398	**0.7862**	0.7580	0.5419	**0.6758**	0.6277	**0.8067**	0.7291	0.6024
QS Trachea	0.5148	0.5055	**0.5227**	**0.2335**	0.1969	0.1980	**0.5073**	0.4923	0.4470
QS Lung	**0.6766**	0.6610	0.6484	0.2797	0.2792	**0.3059**	**0.4963**	0.3232	0.3750
Muraro	**0.7619**	0.7066	0.6810	**0.6413**	0.4837	0.5015	0.3820	**0.3936**	0.2866
Qx Bladder	0.5006	**0.5304**	0.5172	0.2369	**0.2537**	0.2231	**0.5240**	0.2835	0.3860
Klein	**0.6848**	0.6390	0.5736	**0.7148**	0.6586	0.4751	**0.6035**	0.5336	0.4006
Romanov	**0.5205**	0.4939	0.5136	**0.3609**	0.2049	0.2468	**0.2974**	0.2460	0.2366
Adam	**0.6784**	0.6601	0.6568	**0.5090**	0.5069	0.5016	**0.5070**	0.4476	0.3770
Qx Limb Muscle	**0.7569**	0.7461	0.7148	0.4983	**0.5534**	0.4846	**0.7551**	0.5808	0.6185
QS Heart	0.6039	**0.6075**	0.5697	0.2497	**0.2691**	0.2149	**0.5677**	0.4434	0.3455
Young	0.6536	**0.6557**	0.6251	**0.5066**	0.4855	0.4231	**0.2597**	0.1997	0.1220
Plasschaert	0.5563	0.5206	**0.5590**	**0.3540**	0.2578	0.3513	**0.3252**	0.2186	0.1099
Qx Spleen	**0.4287**	0.4118	0.4194	**0.1314**	0.1138	0.1274	**0.5957**	0.4961	0.2248
Chen	0.6703	**0.6990**	0.6521	0.4631	**0.5119**	0.4322	**0.2800**	0.2742	0.2631
Tosches turtle	-	-	-	-	-	-	-	-	-
Bach	-	-	-	-	-	-	-	-	-
Average	**0.6459**	0.6398	0.6236	**0.4446**	0.4420	0.4108	**0.5005**	0.4097	0.3593

The '-' is due to the fact that scGAE cannot run them even with large memory.

Supplementary Table 17. Comparison performance of GraphSCC with different numbers of highly variable genes measured by NMI, ARI and ASW

Dataset	NMI					ARI					ASW				
	300	500	1000	1500	2000	300	500	1000	1500	2000	300	500	1000	1500	2000
Pollen	0.9195	0.9148	0.9174	0.8818	**0.9233**	0.8826	**0.9240**	0.9040	0.8458	0.9167	0.6954	0.6813	0.6481	0.6906	**0.7625**
Camp-Brain	0.4658	0.4347	0.4706	**0.4728**	0.4683	0.2817	0.2498	0.3111	**0.3215**	0.3119	**0.2422**	0.1383	0.1633	0.1055	0.1016
Camp-Liver	**0.7637**	0.6688	0.6997	0.6144	0.6354	**0.5661**	0.4315	0.4517	0.4011	0.4033	**0.4737**	0.4645	0.4504	0.2867	0.3308
QS Diaphragm	0.6664	**0.8966**	0.6453	0.8888	0.8752	0.4671	**0.9519**	0.4663	0.9474	0.9421	0.5572	0.6932	0.6588	**0.7977**	0.7363
QS Limb Muscle	0.7025	0.7009	**0.9214**	0.9050	0.9080	0.5329	0.5303	**0.9571**	0.9314	0.9412	0.4684	**0.7577**	0.7568	0.7189	0.6572
QS Trachea	0.6080	0.7102	0.8010	0.8104	**0.8123**	0.6784	0.8020	0.8127	0.8476	**0.8468**	0.4368	0.5169	0.5069	0.5737	**0.6606**
QS Lung	0.6586	0.6824	0.7334	0.6629	**0.7569**	0.5189	0.5180	0.7084	0.6050	**0.7348**	0.2598	0.4450	0.4630	0.4692	**0.4942**
Muraro	0.5830	0.5723	0.7632	0.7731	**0.7885**	0.3789	0.3391	0.7454	0.7539	**0.7741**	0.1549	0.4759	0.5889	**0.6053**	0.5351
Qx Bladder	0.3825	0.4541	0.3572	**0.9508**	0.9264	0.1603	0.1638	0.1612	**0.9815**	0.9605	**0.6448**	0.5697	0.4663	0.5159	0.5270
Klein	0.6401	**0.7484**	0.7391	0.7243	0.7202	0.6638	**0.7789**	0.7518	0.7227	0.7090	0.4645	0.5936	0.6526	0.6814	**0.6918**
Romanov	0.3808	0.6080	**0.6241**	0.6138	0.5752	0.2296	0.4501	**0.4564**	0.4436	0.4297	0.1997	0.2498	0.3196	0.3244	**0.3805**
Adam	0.5533	0.5606	0.5698	0.5776	**0.5779**	0.2381	0.2444	0.2482	0.2602	**0.2633**	0.4619	0.5022	**0.5033**	0.4603	0.4754
Qx Limb Muscle	0.4378	0.6501	**0.8182**	0.7920	0.7763	0.2092	0.4534	**0.8066**	0.7405	0.6421	0.5165	0.7410	**0.7731**	0.7400	0.7073
QS Heart	0.6614	0.8350	0.8449	0.8421	**0.8474**	0.6643	0.8908	0.8963	0.8953	**0.9008**	0.3843	**0.5709**	0.5621	0.4993	0.5206
Young	0.5633	0.6047	0.6290	**0.6533**	0.6460	0.3712	0.3795	0.4153	**0.4470**	0.4183	0.2345	0.2033	0.2444	**0.3828**	0.3246
Plasschaert	0.4508	0.7489	0.7994	**0.8035**	0.7832	0.2619	0.7965	**0.8681**	0.8648	0.8396	0.1621	0.2541	0.3073	0.3358	**0.3762**
Qx Spleen	0.4496	**0.6581**	0.5050	0.4420	0.4243	0.3974	**0.7874**	0.5633	0.4387	0.4138	0.3627	0.4504	**0.4623**	0.3596	0.4346
Chen	0.5144	0.6497	**0.7046**	0.6979	0.7039	0.2531	0.4543	**0.5743**	0.5727	0.5696	0.1774	0.3166	0.2956	0.2988	**0.3266**
Tosches turtle	0.6442	0.6937	0.7027	0.6940	**0.7102**	0.4986	0.5555	0.6176	0.5321	**0.6318**	0.1947	0.3035	0.2832	0.2784	**0.3599**
Bach	0.4966	**0.6950**	0.5369	0.5446	0.5370	0.3897	**0.6546**	0.4012	0.4063	0.3988	0.2092	0.3468	0.3323	**0.3897**	0.3780
Average	0.5771	0.6744	0.6891	0.7173	**0.7198**	0.4322	0.5678	0.6059	0.6480	**0.6524**	0.3650	0.4637	0.4719	0.4757	**0.4890**

Supplementary Table 18. Comparison performance of GraphSCC with different hidden layer numbers measured by NMI, ARI and ASW. GraphSCC1 is with ([Input layer]-64-10), GraphSCC2 is with ([Input layer]-256-64-10), and GraphSCC3 is with ([Input layer]-512-256-64-10)

Dataset	NMI			ARI			ASW		
	GraphSCC1	GraphSCC2	GraphSCC3	GraphSCC1	GraphSCC2	GraphSCC3	GraphSCC1	GraphSCC2	GraphSCC3
Pollen	0.9107	0.8032	**0.9233**	**0.9274**	0.8962	0.9167	0.7512	0.7594	**0.7625**
Camp-Brain	0.2866	0.3964	**0.4683**	0.2236	0.2636	**0.3119**	**0.119**	0.0930	0.1016
Camp-Liver	0.5608	0.5791	**0.6354**	0.3618	0.3884	**0.4033**	0.3086	0.2701	**0.3308**
QS Diaphragm	0.8637	**0.8764**	0.8752	0.9346	**0.9478**	0.9421	**0.7471**	0.7447	0.7363
QS Limb Muscle	0.8514	0.8635	**0.9080**	0.9128	0.9136	**0.9412**	0.6182	0.6189	**0.6572**
QS Trachea	**0.8381**	0.8134	0.8123	**0.8586**	0.6782	0.8468	0.5514	0.5869	**0.6606**
QS Lung	0.7354	0.7117	**0.7569**	0.7207	0.7053	**0.7348**	**0.5020**	0.4925	0.4942
Muraro	0.6932	0.6969	**0.7885**	0.6488	0.6531	**0.7741**	0.5318	0.5337	**0.5351**
Qx Bladder	0.9129	**0.9306**	0.9264	0.9600	**0.9677**	0.9605	0.5144	**0.5446**	0.5270
Klein	0.7112	**0.7804**	0.7202	0.7206	**0.7683**	0.7090	0.6585	**0.6941**	0.6918
Romanov	0.4460	0.4430	**0.5752**	**0.4346**	0.4109	**0.4297**	**0.3887**	**0.4050**	**0.3805**
Adam	0.4292	0.4525	**0.5779**	0.3508	**0.3665**	0.2633	0.4435	0.4546	**0.4754**
Qx Limb Muscle	0.7398	**0.7982**	0.7763	0.6169	**0.6819**	0.6421	0.6877	**0.7291**	0.7073
QS Heart	0.9038	**0.9102**	0.8474	0.8406	0.8628	**0.9008**	**0.6011**	0.5964	0.5206
Young	0.6025	0.5332	**0.6460**	0.4084	0.3223	**0.4183**	**0.4114**	0.2594	0.3246
Plasschaert	**0.8148**	0.7655	0.7832	**0.8541**	0.8295	0.8396	0.3660	0.3472	**0.3762**
Qx Spleen	0.5497	**0.5848**	0.4243	0.5120	**0.5615**	0.4138	0.4012	**0.4788**	0.4346
Chen	**0.7829**	0.7002	0.7039	**0.6014**	0.5237	0.5696	0.2653	**0.3739**	0.3266
Tosches turtle	0.6513	0.3906	**0.7102**	0.6223	0.5762	**0.6318**	0.2483	0.3395	**0.3599**
Bach	**0.7637**	0.6650	0.5370	**0.7904**	0.7191	0.3988	**0.4673**	0.4501	0.3780
Average	0.7024	0.6847	**0.7198**	**0.6650**	0.6518	0.6524	0.4791	0.4886	**0.4890**

Supplementary Note 2: Comparison of scMGCA and comparative methods for visualization of clustering results on 20 datasets via UMAP

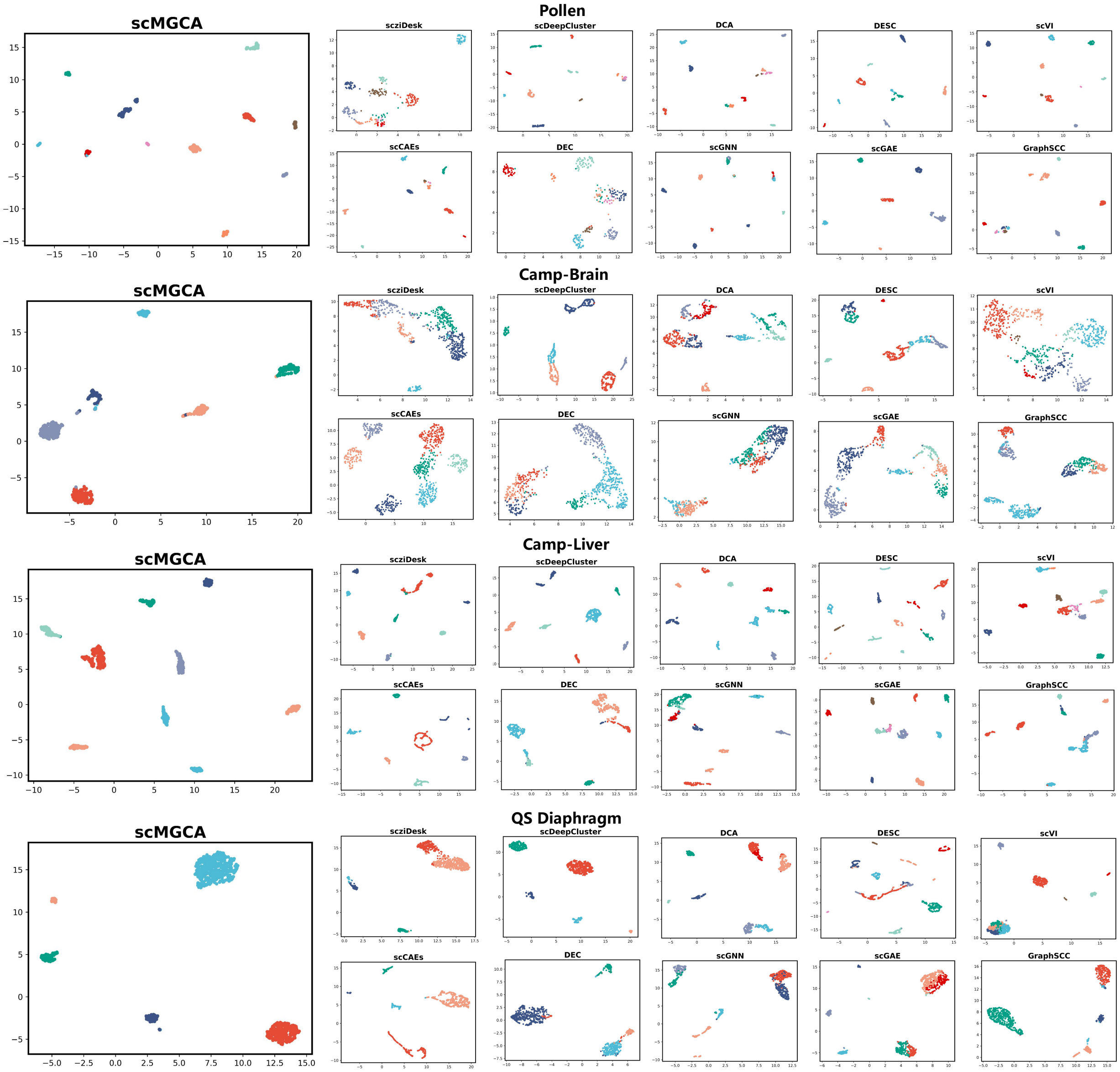

Supplementary Fig. 3. Comparison of clustering results with 2D visualization by UMAP on the ‘Pollen’, ‘Camp-Brain’, ‘Camp-Liver’, and ‘QS Diaphragm’ dataset.

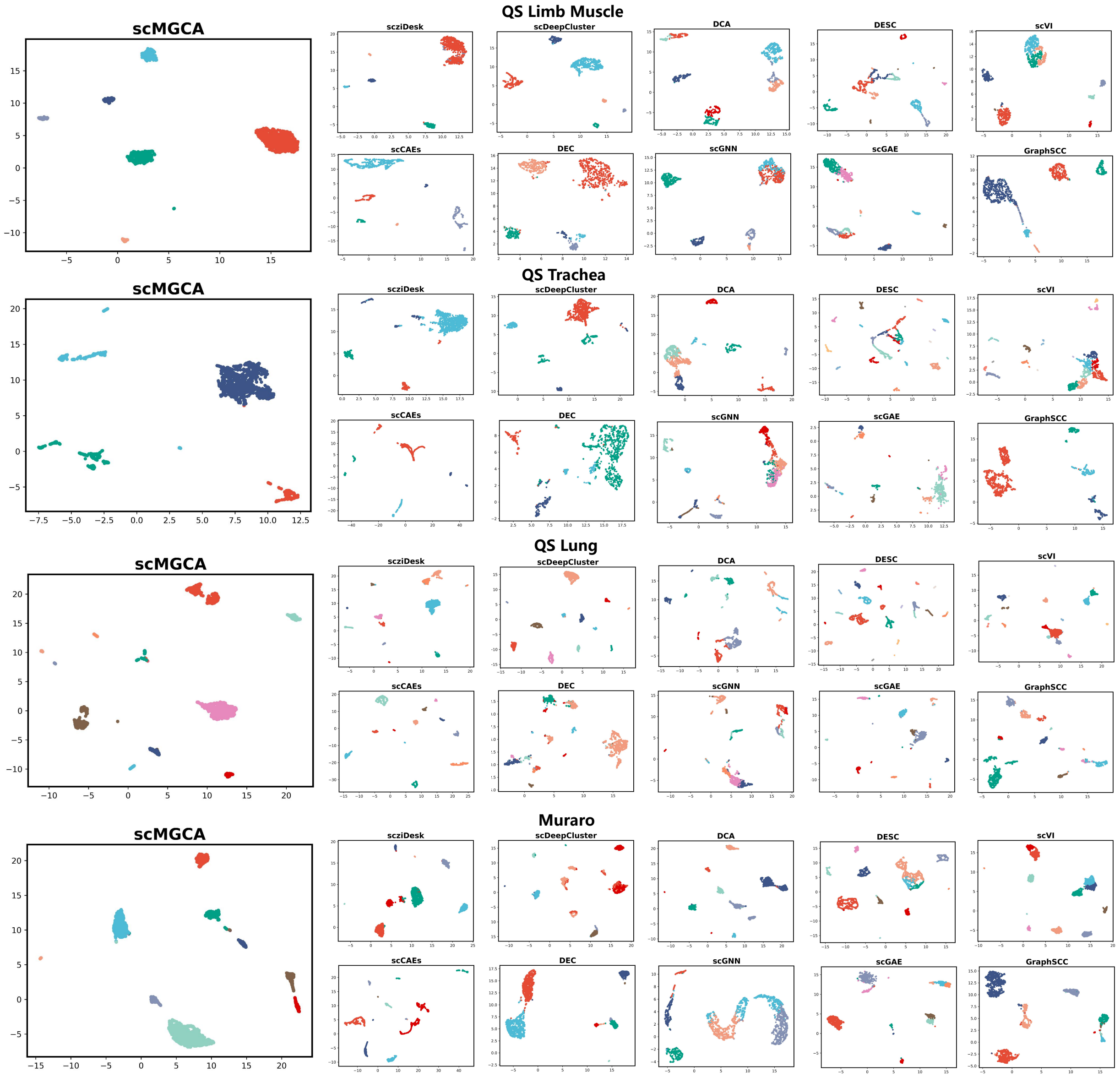

Supplementary Fig. 4. Comparison of clustering results with 2D visualization by UMAP on the 'QS Limb Muscle', 'QS Trachea', 'QS Lung', and 'Muraro' dataset.

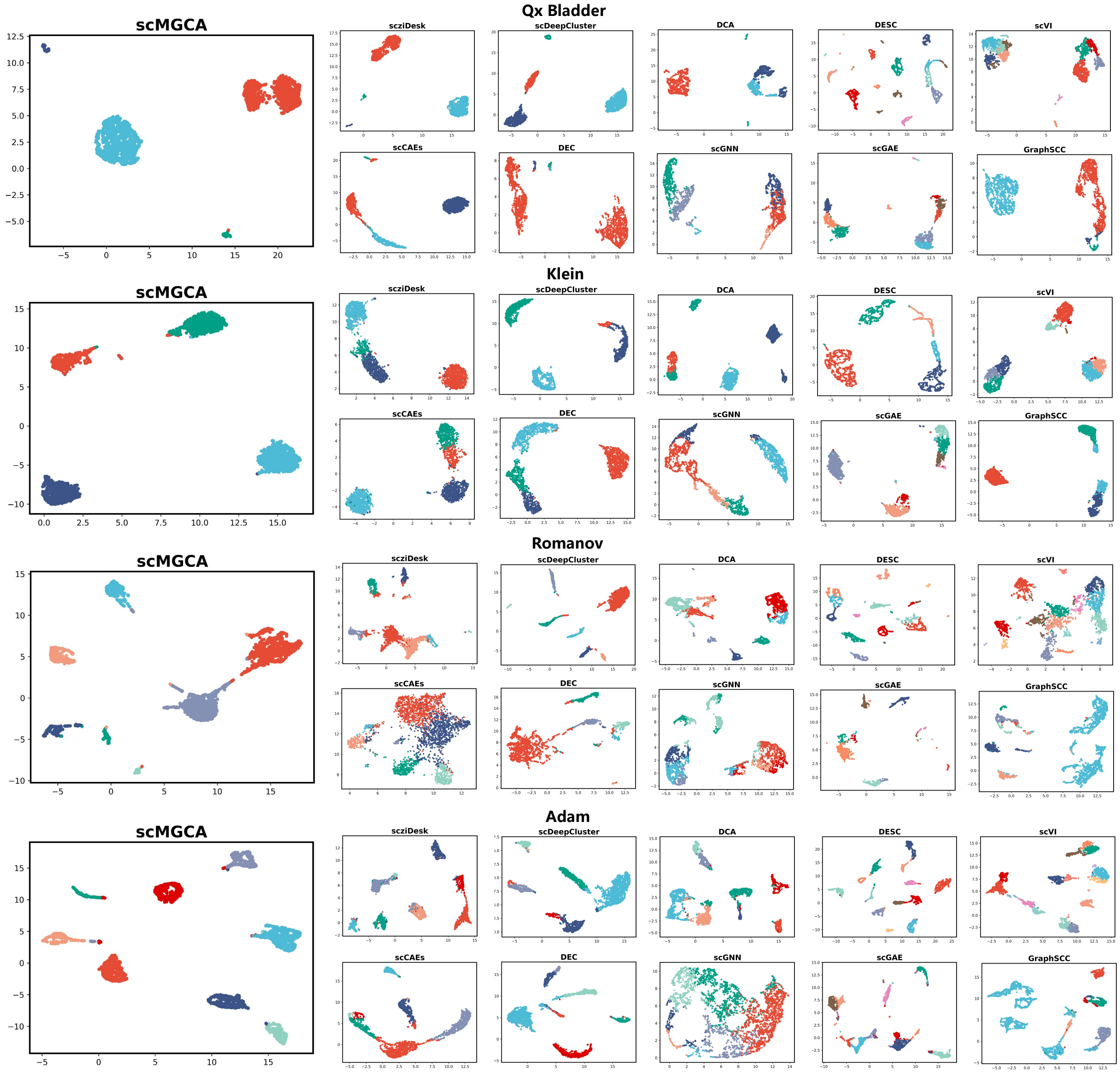

Supplementary Fig. 5. Comparison of clustering results with 2D visualization by UMAP on the ‘Qx Bladder’, ‘Klein’, ‘Romanov’, and ‘Adam’ dataset.

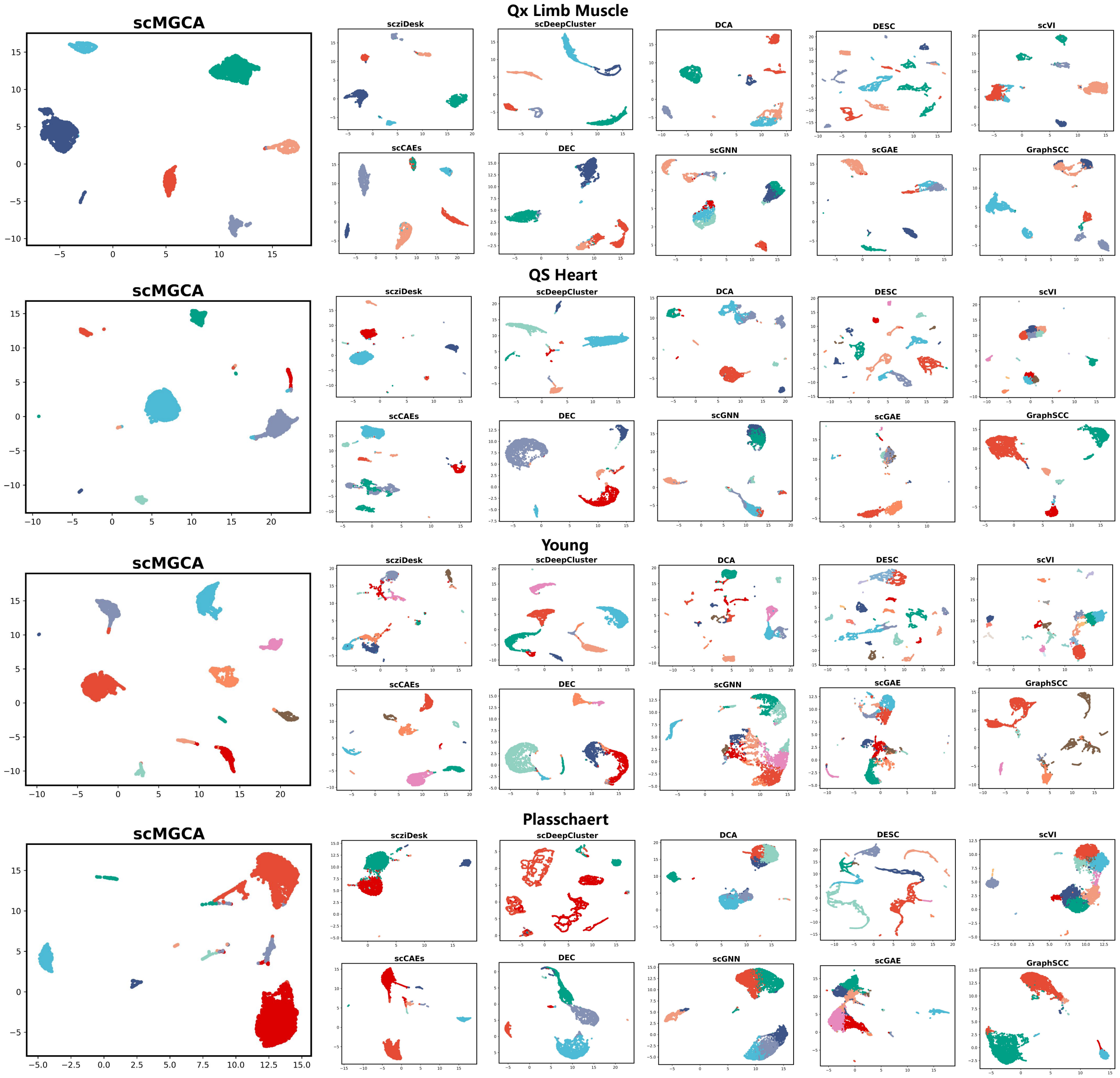

Supplementary Fig. 6. Comparison of clustering results with 2D visualization by UMAP on the ‘Qx Limb Muscle’, ‘QS Heart’, ‘Young’, and ‘Plasschaert’ dataset.

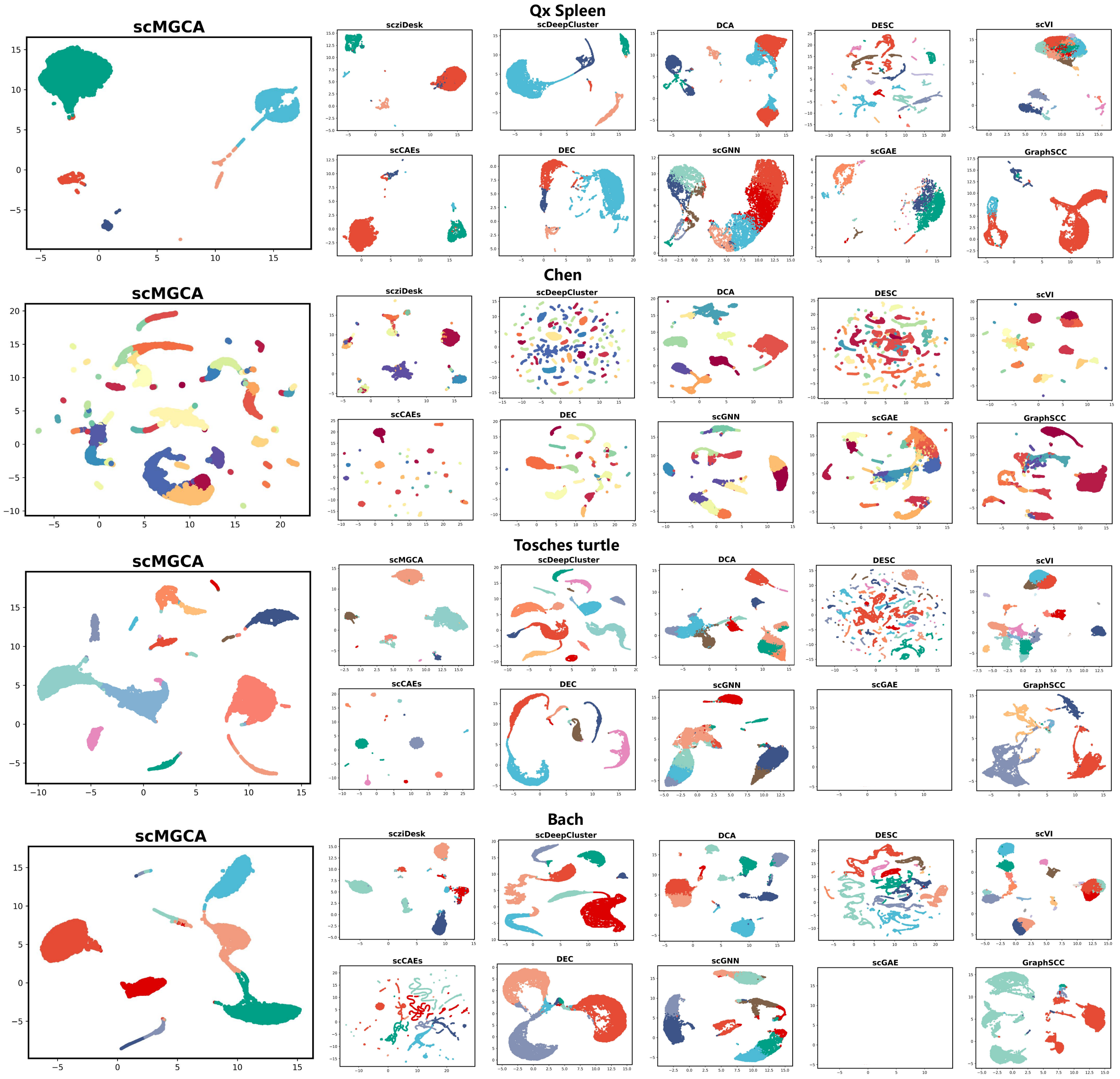

Supplementary Fig. 7. Comparison of clustering results with 2D visualization by UMAP on the ‘Qx Spleen’, ‘Chen’, ‘Tosches turtle’, and ‘Bach’ dataset. The blank figures generated by scGAE are due to the fact that scGAE cannot run them even with large memory.

Supplementary Note 3: scMGCA can detect rare cell types and small clusters that cannot be detected by other methods

We compared scMGCA with other deep learning methods including scziDesk, scDeepCluster, DCA, DEC, scGNN, scGAE, and GraphSCC on four datasets ('QS_heart', 'Muraro', 'Qx_limb Muscle', and 'Adam') that contain the rare cell types and small clusters for in-depth examination of the effectiveness. To construct a fair and explicit comparison, we first applied t-SNE to project the raw single-cell data into a 2D space and visualized it using true labels. After that, we applied the cluster labels obtained by scMGCA and the other deep learning methods, including scziDesk, scDeepCluster, DCA, DEC, scGNN, scGAE, and GraphSCC (Supplementary Fig. 8) to visualize in the same 2D space. We observe that scMGCA can detect rare cell types and small clusters that the other methods cannot pick up on those datasets, as depicted in Supplementary Fig. 8. Indeed, for the 'QS Heart' dataset, only scMGCA was able to accurately detect two small clusters of smooth muscle cells and myofibroblast cells, which were aggregated by the other methods. A similar situation was seen in the 'Muraro' dataset, where only scMGCA and DCA could distinguish delt and gamma cells; the other methods either merged these two cell types into one group (e.g., scziDesk, scDeepCluster, and scGAE), or grouped the two cell types as in other larger cell populations (e.g., DEC, scGNN, and GraphSCC). Furthermore, scMGCA, scziDesk and scGAE could accurately detect T cells in the 'Qx Limb Muscle' dataset, while the remaining methods assigned T cells to other types of cell clusters. In particular, compared to most non-GNN-based methods (except DEC) that successfully identified delta cells in the 'Adam' dataset, all GNN-based methods failed, except scMGCA which still correctly identified delta cells in this situation. Overall, our proposed scMGCA either outperforms or complements existing approaches in identifying cell types and could reliably detect rare cell types and small clusters.

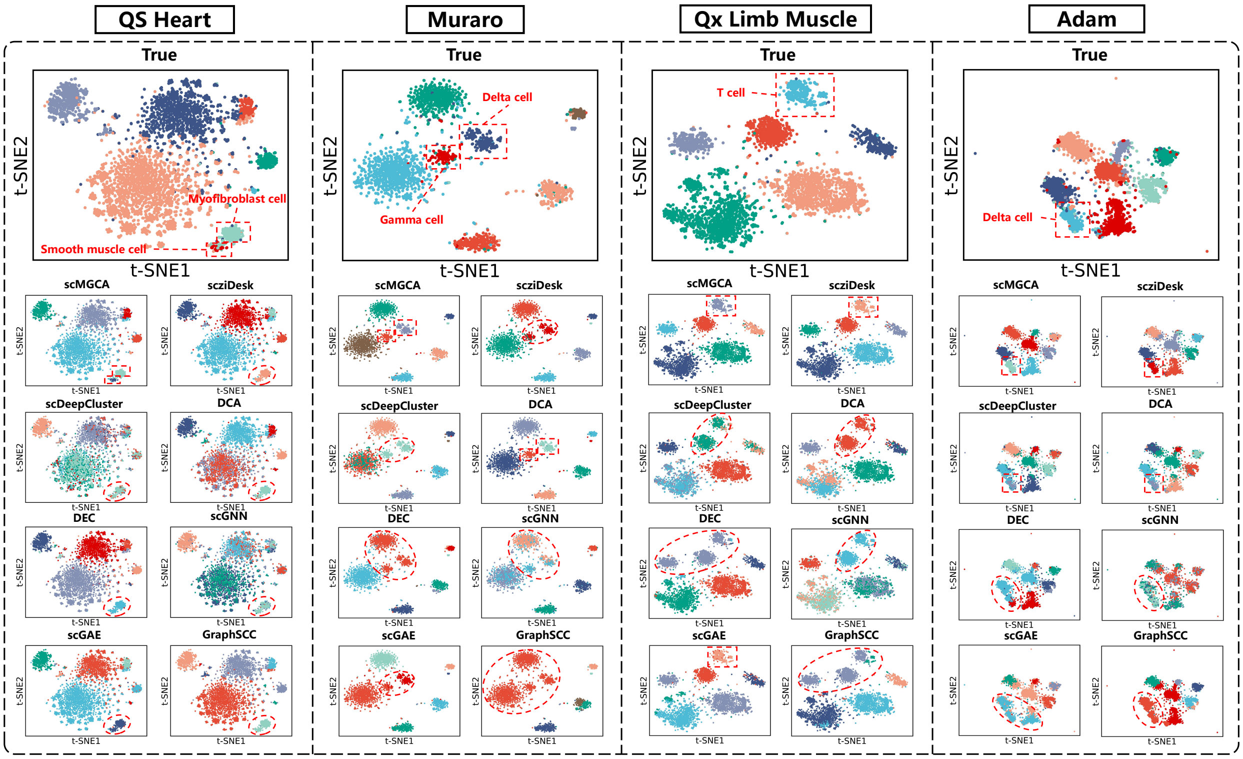

Supplementary Fig. 8. scMGCA can detect rare cell types and small clusters.

Supplementary Note 4: Running time of scMGCA under different numbers of cells and genes on 'Tabula Muris'

To evaluate how running time is changed along the number of cells and number of genes, we also tested the respective running time of scMGCA for different numbers of cells and different numbers of genes on 'Tabula Muris' (Supplementary Fig. 9). Specifically, we randomly selected 5000, 10000, 20000, 30000, 40000, 50000, 60000, and 70000 cells from the original 70118 cells, to evaluate the impact from the number of cells. For the number of genes, we selected 500, 1000, 2000, 3000, 4000, 5000, 6000, and 7000 highly variable genes using the Scanpy package, to demonstrate the effect of different numbers of genes. It can be observed from Supplementary Fig. 9 that scMGCA demonstrates a monotonically linear increase in running time along with the number of cells and genes, demonstrating its computational scalability.

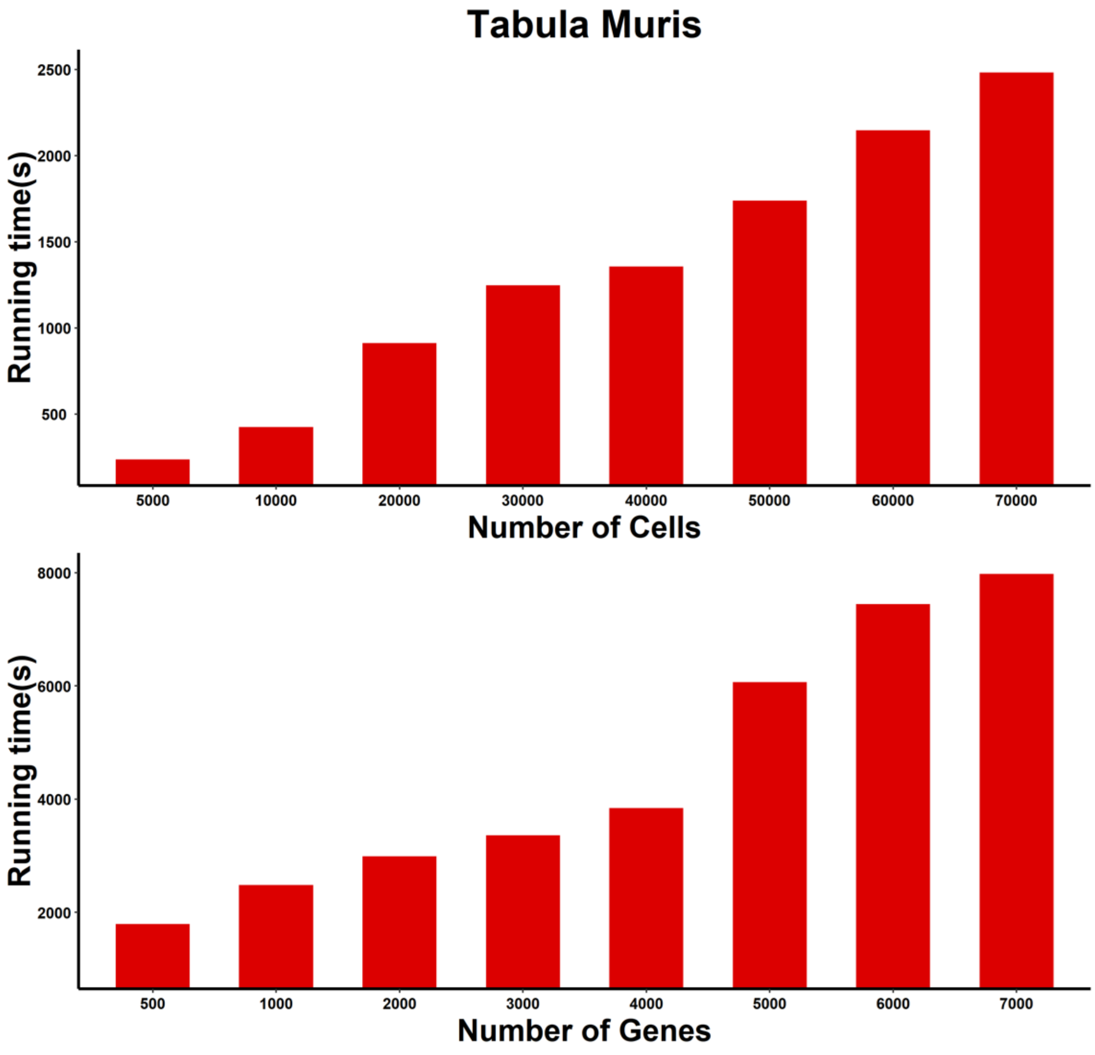

Supplementary Fig. 9. Running time of scMGCA with different numbers of cells or genes on 'Tabula Muris'. Source data are provided as a Source Data file.

Supplementary Note 5: Analysis of the mouse brain data with 1.3 million cells

The original dataset was downloaded from the 10x Genomics website which contains 1,306,127 cells and 27,998 genes: https://support.10xgenomics.com/single-cell-gene-expression/datasets/1.3.0/1M_neurons.

Data preprocessing: We used SCANPY to preprocess the dataset: 1. Cells and genes were filtered using "$scanpy.pp.filter\_cells$" with (min_counts=200) and "$scanpy.pp.filter\_genes$" with (min_counts=20), respectively. 2. Gene expression levels were normalized using a "$scanpy.pp.normalize\_total$" with (target_sum=1e4). 3. The data was log-transformed using "$scanpy.pp.log1p$". 4. The data after normalization was used with "$scanpy.pp.highly\_variable\_genes$" (min_mean=0.0125, max_mean=3, min_disp=0.5, n_top_genes = 1000) to identify the top 1000 highly variable genes.

Parameters setting: For the 1.3 million dataset, we used the GCN-based encoder of scMGCA comprising two layers, with the first hidden layer containing 32 nodes and the second hidden layer including 20 nodes. The decoder and encoder are symmetrical, thus the neural network architecture is [1000, 32, 20, 32, 1000]. The initialization center adopted the Leiden algorithm, and the resolution was set to 0.2. The remaining settings included pretrain_epoch = 200, maxiter = 100, and batch = 2000. It is worth noting that the number of training epochs of DEC was the same as that of scMGCA, and other parameters were used by default. DESC used the parameters set in its paper for analyzing 1.3 million dataset. For Seurat, SHARP and SCANPY, we followed the default parameters.

Analysis of the mouse brain data containing 1.3 million cells. Supplementary Fig. 10 depicts the 2D visualization of the latent embedding representation of scMGCA using t-SNE, with the Leiden method identifying 14 clusters. In addition, we compared the running time and memory usage of scMGCA and the compared methods on the 1.3 million cell dataset, including DEC, DESC, Seurat, SHARP, and SCANPY. For additional comparisons, we randomly selected 10,000, 30,000, 50,000, 1,000,000, 1,300,000 cells from the 1.3 million cell dataset, and evaluated the running time and memory usage in these 5 different cases (Supplementary Fig. 11). For the traditional methods, the running time is shorter than other methods, which is largely consistent with the evaluation in (1). However, we can observe that Seurat and SHARP require more than 60GB of memory and SCANPY requires nearly 40GB of memory to analyze 1.3 million cells, rendering them inaccessible to the majority of researchers who lack enough computational resources. Meanwhile, although the running time of scMGCA is high, it is still within a reasonable range and scMGCA is the only GNN-based method (in contrast to scGNN, scGAE, and GraphSCC) that was able to be successfully run on an 1.3 million cell dataset. In terms of memory consumption, DESC outperforms the other methods. The memory consumption of DEC and scMGCA are both and smaller than the traditional methods. Interestingly, the time and memory requirements of the deep learning method DESC on large-scale datasets is more balanced than other methods. Possibly, for the deep graph learning method scMGCA, the construction and calculation of cell graph can incur additional time and memory costs, resulting in the extra time and memory consumption accordingly. Moreover, since a small number of pretrain_epoch may not be helpful for further analysis, our model increases the number of pretrain_epoch to 200 as opposed to DESC, which only evaluates pretrain_epoch 10 times on large-scale data; therefore, it greatly increases the final running time.

In summary, we can observe that, excluding the extra time and memory consumption aspects caused by cell graph computation, there is not much difference between scMGCA and deep learning methods. Moreover, it is worth noting that among several

graph neural networks compared in this paper, scMGCA is the only one that can run successfully; it can also be regarded as an advancement in deep graph learning method development.

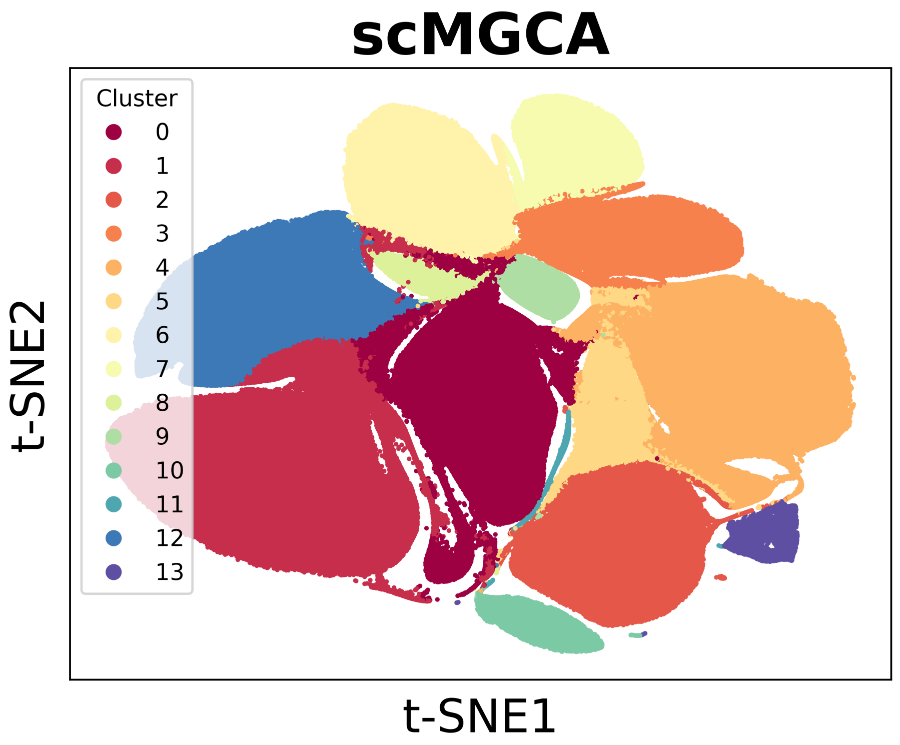

Supplementary Fig. 10. 2D visualization for clustering results of scMGCA on 1.3 million mouse dataset via t-SNE.

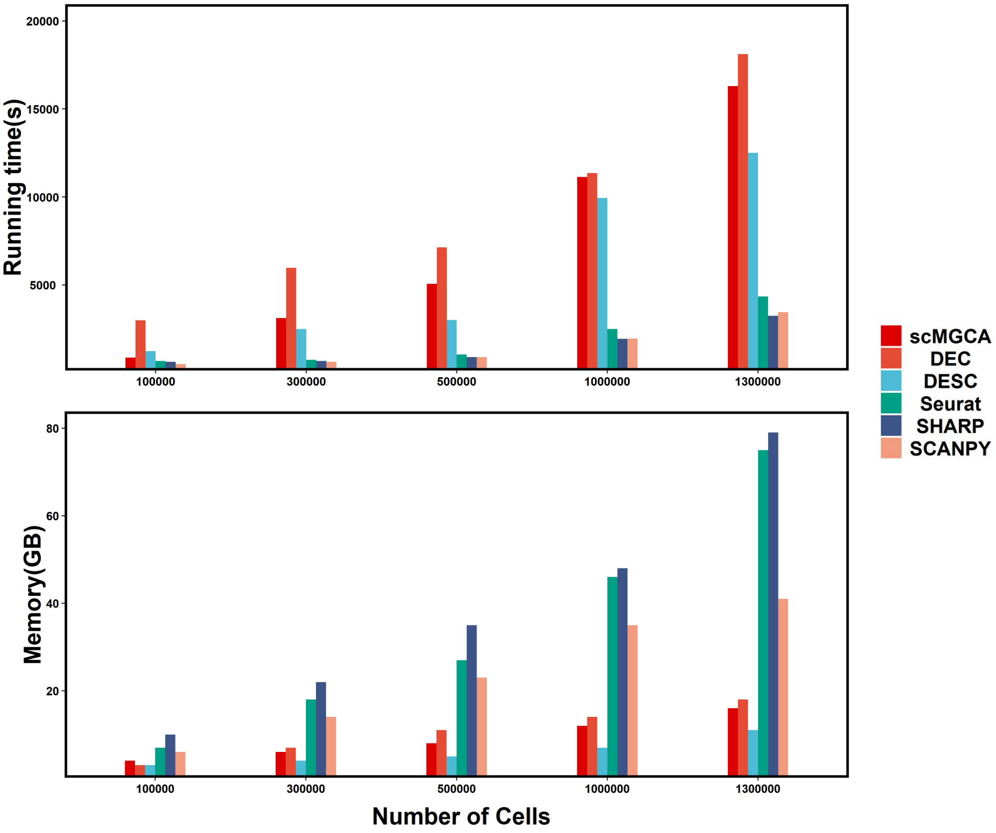

Supplementary Fig. 11. Comparison of running time and memory usage of scMGCA to other methods with different numbers of cells. Source data are provided as a Source Data file.

Supplementary Note 6: Comparison of scMGCA with t-SNE and UMAP across a wider parameter space

We have extended the parameter space of t-SNE and UMAP and conducted experiments to demonstrate the performance comparison. The overview of different parameter cases for t-SNE and UMAP is summarized in Supplementary Table 1 and the comparison results are tabulated in Supplementary Table 2 and Table 3.

For t-SNE, there are three adjustable parameters including “*perplexity*”, “*metric*”, and “*init*” respectively. The “*perplexity*” is related to the number of nearest neighbors for the manifold learning algorithm, generally between 5 and 50. We enumerated the value among the set [10, 30, 50]. The “*metric*” is distance function between instances in the feature space, we enumerated four kinds of distance methods: Euclidean distance, cosine distance, Pearson correlation coefficient, and Minkowski distance. The “*init*” is the initialization method of embedding; it is mainly divided into random sampling and PCA. For UMAP, there are also three important parameters, including “*n_neighbors*”, “*metric*”, and “*min_dist*” respectively. The “*n_neighbors*” is the size of the local neighborhood for manifold approximation, we chose the value from the set [10, 15, 20]. The “*metric*” is the same as t-SNE, we still chose four distance methods. The “*metric*” is the effective minimum distance between the embedding points, generally between 0.001 and 0.5, we chose the value from the set [0.001, 0.01, 0.1]. To provide a comprehensive comparison, we compared different combinations of all the parameters of t-SNE and UMAP, which yielded 24 combinations of t-SNE parameters and 36 combinations of UMAP parameters, as summarized in Supplementary Table 1.

Then, we compared these cases for scMGCA on 20 single-cell datasets using the average silhouette width (ASW) (Supplementary Table 2 and Table 3). It can be observed that scMGCA still has good dimensionality reduction performance as compared to the best cases of t-SNE and UMAP on each dataset. Specifically, we have 19 and 15 out of the 20 datasets outperforming t-SNE and UMAP, respectively. In particular, the average ASW of scMGCA for the 20 datasets is also superior to the best case 19 (p=50, m=cosine, i=pca) and case 29 (n=20, m=cosine, d=0.01) for t-SNE and UMAP, respectively. It is worth noting that the ASW of UMAP and t-SNE will be improved when the “*metric*” is chosen as “cosine” and “correlation”.

Supplementary Table 19. Overview of different parameter cases for t-SNE and UMAP

t-SNE				UMAP					
p = perplexity m = metric i = init				n = n_neighbors m = metric d = min_dist					
case1	p=10,m=euclidean,i=pca	case13	p=30,m=correlation,i=pca	case1	n=10,m=euclidean,d=0.001	case13	n=15,m=euclidean,d=0.001	case25	n=20,m=euclidean,d=0.001
case2	p=10,m=euclidean,i=random	case14	p=30,m=correlation,i=random	case2	n=10,m=euclidean,d=0.01	case14	n=15,m=euclidean,d=0.01	case26	n=20,m=euclidean,d=0.01
case3	p=10,m=cosine,i=pca	case15	p=30,m=minkowski,i=pca	case3	n=10,m=euclidean,d=0.1	case15	n=15,m=euclidean,d=0.1	case27	n=20,m=euclidean,d=0.1
case4	p=10,m=cosine,i=random	case16	p=30,m=minkowski,i=random	case4	n=10,m=cosine,d=0.001	case16	n=15,m=cosine,d=0.001	case28	n=20,m=cosine,d=0.001
case5	p=10,m=correlation,i=pca	case17	p=50,m=euclidean,i=pca	case5	n=10,m=cosine,d=0.01	case17	n=15,m=cosine,d=0.01	case29	n=20,m=cosine,d=0.01
case6	p=10,m=correlation,i=random	case18	p=50,m=euclidean,i=random	case6	n=10,m=cosine,d=0.1	case18	n=15,m=cosine,d=0.1	case30	n=20,m=cosine,d=0.1
case7	p=10,m=minkowski,i=pca	case19	p=50,m=cosine,i=pca	case7	n=10,m=correlation,d=0.001	case19	n=15,m=correlation,d=0.001	case31	n=20,m=correlation,d=0.001
case8	p=10,m=minkowski,i=random	case20	p=50,m=cosine,i=random	case8	n=10,m=correlation,d=0.01	case20	n=15,m=correlation,d=0.01	case32	n=20,m=correlation,d=0.01
case9	p=30,m=euclidean,i=pca	case21	p=50,m=correlation,i=pca	case9	n=10,m=correlation,d=0.1	case21	n=15,m=correlation,d=0.1	case33	n=20,m=correlation,d=0.1
case10	p=30,m=euclidean,i=random	case22	p=50,m=correlation,i=random	case10	n=10,m=minkowski,d=0.001	case22	n=15,m=minkowski,d=0.001	case34	n=20,m=minkowski,d=0.001
case11	p=30,m=cosine,i=pca	case23	p=50,m=minkowski,i=pca	case11	n=10,m=minkowski,d=0.01	case23	n=15,m=minkowski,d=0.01	case35	n=20,m=minkowski,d=0.01
case12	p=30,m=cosine,i=random	case24	p=50,m=minkowski,i=random	case12	n=10,m=minkowski,d=0.1	case24	n=15,m=minkowski,d=0.1	case36	n=20,m=minkowski,d=0.1

Supplementary Table 20. The ASW comparison of t-SNE and scMGCA

Datasets	scMGCA	t-SNE																							
		case1	case2	case3	case4	case5	case6	case7	case8	case9	case10	case11	case12	case13	case14	case15	case16	case17	case18	case19	case20	case21	case22	case23	case24
Pollen	0.7286	0.4778	0.4775	0.6885	0.7063	0.6902	0.6742	0.3965	0.4051	0.5117	0.5191	0.6627	0.6591	0.6470	0.6347	0.4696	0.438	0.4834	0.4802	0.6346	0.6429	0.6193	0.6143	0.4961	0.5024
Camp-Brain	0.2201	0.0688	0.0565	0.0657	0.0537	0.0742	0.0612	0.0650	0.0617	0.0626	0.0733	0.0744	0.0732	0.0997	0.0986	0.0026	0.0207	0.0946	0.1086	0.1230	0.1166	0.1161	0.1025	0.0179	0.0379
Camp-Liver	0.6177	0.5094	0.5013	0.4499	0.4742	0.4545	0.4646	0.4618	0.4648	0.5557	0.5658	0.5594	0.5653	0.5592	0.5737	0.5186	0.5253	0.5658	0.5740	0.5804	0.5851	0.5802	0.5959	0.5273	0.5293
QS Diaphragm	0.8458	0.2620	0.2704	0.4408	0.4391	0.3842	0.3758	0.2538	0.2677	0.2597	0.2700	0.5152	0.5093	0.4629	0.4626	0.2809	0.2791	0.2995	0.2835	0.5249	0.5294	0.4554	0.4644	0.2559	0.2687
QS Limb Muscle	0.8386	0.2740	0.2659	0.4195	0.4247	0.3959	0.3741	0.2776	0.2571	0.2808	0.2747	0.5345	0.5323	0.4720	0.4797	0.3082	0.2720	0.2862	0.2823	0.5585	0.5528	0.4935	0.4871	0.2944	0.2801
QS Trachea	0.7261	0.2018	0.2041	0.2223	0.2699	0.1995	0.2400	0.2039	0.1946	0.2397	0.2441	0.3627	0.3435	0.2846	0.3210	0.1970	0.2416	0.2287	0.2246	0.4042	0.4085	0.3174	0.3596	0.2213	0.2367
QS Lung	0.5414	0.1855	0.2334	0.2319	0.2345	0.2452	0.2393	0.2059	0.2570	0.2262	0.2436	0.3549	0.3666	0.3406	0.359	0.2142	0.2162	0.2511	0.2386	0.4260	0.4434	0.4170	0.4060	0.2229	0.2184
Muraro	0.5584	0.3250	0.3211	0.3401	0.3173	0.2786	0.2736	0.3001	0.3198	0.3949	0.3826	0.3919	0.3782	0.3373	0.3408	0.3875	0.3755	0.4030	0.4081	0.4294	0.3801	0.3658	0.3755	0.3840	0.4116
Qx Bladder	0.8105	0.2471	0.3021	0.3069	0.3663	0.2971	0.2357	0.2510	0.2814	0.3137	0.3476	0.4449	0.5167	0.4314	0.3878	0.3208	0.3421	0.2752	0.2708	0.4758	0.4937	0.4435	0.4117	0.2806	0.3057
Klein	0.4544	0.0843	0.0787	0.2466	0.2439	0.2415	0.2405	0.0585	0.0454	0.1555	0.1555	0.3193	0.2199	0.3056	0.2864	0.0786	0.0686	0.1778	0.1778	0.3376	0.3231	0.3371	0.3482	0.1013	0.0894
Romanov	0.5416	0.1562	0.1334	0.1715	0.1391	0.1453	0.1200	0.1452	0.0968	0.1620	0.1522	0.2298	0.2336	0.2139	0.2097	0.1515	0.1796	0.1735	0.1654	0.2731	0.2633	0.2532	0.2428	0.1632	0.1553
Adam	0.6495	0.3392	0.3526	0.3696	0.3822	0.3761	0.3604	0.3376	0.3309	0.3850	0.3958	0.4210	0.4346	0.4211	0.4429	0.3780	0.3953	0.4026	0.4010	0.4601	0.4527	0.4590	0.4501	0.3756	0.3667
Qx Limb Muscle	0.8207	0.4026	0.4121	0.3529	0.3628	0.3336	0.3681	0.4069	0.4159	0.4771	0.4739	0.4826	0.4986	0.4781	0.4625	0.4792	0.5075	0.5100	0.5156	0.5468	0.5406	0.5551	0.5207	0.5192	0.5160
QS Heart	0.6643	0.1538	0.1921	0.1873	0.1185	0.0755	0.1046	0.2223	0.2029	0.2578	0.2624	0.2383	0.2228	0.2160	0.2293	0.2477	0.2630	0.2740	0.2570	0.3245	0.2743	0.2966	0.2721	0.2697	0.2757
Young	0.3955	0.2141	0.1918	0.1473	0.1455	0.1492	0.1543	0.2350	0.1781	0.2830	0.2627	0.2290	0.2117	0.2479	0.2072	0.2754	0.2526	0.2946	0.2568	0.2524	0.2180	0.2553	0.2294	0.2783	0.2320
Plasschaert	0.5319	0.0930	0.0462	-0.0476	-0.0929	-0.1235	-0.1397	0.0240	-0.0137	0.0540	0.0045	0.0805	-0.0282	0.0514	-0.053	-0.0513	0.0006	0.0742	0.037	0.0721	0.0375	-0.0395	-0.0292	0.0422	0.0459
Qx Spleen	0.6455	0.1951	0.2019	0.1565	0.0766	0.1574	0.1037	0.1743	0.1975	0.1806	0.1215	0.2285	0.2128	0.1958	0.1852	0.1574	0.1112	0.1705	0.1676	0.2529	0.2519	0.2024	0.1797	0.1466	0.1358
Chen	0.1510	0.0650	0.1019	0.0619	0.0815	0.0360	-0.0075	0.0668	0.0755	0.1184	0.1891	0.1778	0.1604	0.1481	0.1646	0.1306	0.1494	0.1693	0.1756	0.2101	0.2032	0.1411	0.1638	0.1691	0.1480
Tosches turtle	0.3129	0.0569	0.1222	0.0026	0.0032	0.0101	0.0184	0.0537	0.0854	0.1126	0.1731	0.1098	0.0745	0.1123	0.0758	0.1094	0.1713	0.1217	0.1882	0.1645	0.1430	0.1623	0.1228	0.1634	0.1907
Bach	0.5986	0.1631	0.1773	0.1174	0.0854	0.1229	0.0922	0.1593	0.1736	0.2054	0.2012	0.1658	0.1755	0.1739	0.1776	0.2097	0.2038	0.1918	0.2125	0.2152	0.2046	0.2117	0.2075	0.2112	0.2199
Average	0.5827	0.2237	0.2321	0.2466	0.2416	0.2272	0.2177	0.2150	0.2149	0.2618	0.2656	0.3292	0.3180	0.3099	0.3023	0.2433	0.2507	0.2724	0.2713	0.3633	0.3532	0.3321	0.3262	0.2570	0.2583

Supplementary Table 21. The ASW comparison of UMAP and scMGCA

Datasets	scMGCA	UMAP																							
		case1	case2	case3	case4	case5	case6	case7	case8	case9	case10	case11	case12	case13	case14	case15	case16	case17	case18	case19	case20	case21	case22	case23	case24
Pollen	0.7286	0.5859	0.5709	0.5503	0.7935	0.7928	0.7652	0.7758	0.7793	0.7641	0.5859	0.5709	0.5503	0.5785	0.5661	0.5617	0.789	0.7873	0.7541	0.7881	0.7819	0.7715	0.5785	0.5661	0.5617
Camp-Brain	0.2201	0.0852	0.0964	0.0925	0.1606	0.1650	0.1500	0.1525	0.1606	0.1500	0.0852	0.0964	0.0925	0.0906	0.0901	0.1057	0.1846	0.1896	0.1624	0.1931	0.1750	0.1807	0.0906	0.0901	0.1057
Camp-Liver	0.6177	0.6696	0.659	0.6534	0.6058	0.5896	0.6131	0.6473	0.6093	0.6402	0.6696	0.659	0.6534	0.6521	0.6631	0.6618	0.6589	0.6528	0.6269	0.6844	0.6634	0.6643	0.6521	0.6631	0.6618
QS Diaphragm	0.8458	0.3240	0.3238	0.3208	0.6229	0.6353	0.6154	0.5944	0.5871	0.5553	0.3240	0.3238	0.3208	0.3270	0.3196	0.3269	0.6800	0.6606	0.6330	0.6217	0.6240	0.6137	0.3270	0.3196	0.3269
QS Limb Muscle	0.8386	0.3848	0.3575	0.3806	0.7179	0.7035	0.6516	0.6585	0.6601	0.6276	0.3848	0.3575	0.3806	0.4011	0.3868	0.3788	0.7731	0.7604	0.7178	0.7286	0.7337	0.6790	0.4011	0.3868	0.3788
QS Trachea	0.7261	0.3120	0.3287	0.3124	0.4406	0.4547	0.4052	0.4573	0.3844	0.4013	0.3120	0.3287	0.3124	0.2664	0.2938	0.2500	0.4593	0.4847	0.4178	0.3621	0.4210	0.4087	0.2664	0.2938	0.2500
QS Lung	0.5414	0.2135	0.2972	0.1901	0.4885	0.5009	0.4689	0.5303	0.5211	0.5064	0.2135	0.2972	0.1901	0.2929	0.3031	0.2209	0.5143	0.5385	0.3590	0.5358	0.5313	0.5238	0.2929	0.3031	0.2209
Muraro	0.5584	0.4046	0.3991	0.3744	0.4528	0.4070	0.4255	0.3829	0.361	0.3549	0.4046	0.3991	0.3744	0.4032	0.4108	0.4185	0.4358	0.4312	0.4097	0.3576	0.3471	0.3645	0.4032	0.4108	0.4185
Qx Bladder	0.8105	0.5260	0.5196	0.4691	0.7443	0.7584	0.7068	0.7437	0.7525	0.7264	0.5260	0.5196	0.4691	0.4971	0.4967	0.4429	0.7531	0.7417	0.7089	0.7585	0.7056	0.7328	0.4971	0.4967	0.4429
Klein	0.4544	0.0279	0.0400	0.0348	0.3800	0.3816	0.3698	0.3652	0.3680	0.3634	0.0279	0.0400	0.0348	0.0485	0.0313	0.0600	0.4001	0.4042	0.3922	0.3989	0.4028	0.3850	0.0485	0.0313	0.0600
Romanov	0.5416	0.2483	0.2168	0.2131	0.2813	0.2836	0.2671	0.3164	0.2909	0.2661	0.2483	0.2168	0.2131	0.2304	0.2524	0.2056	0.3239	0.3271	0.2707	0.3063	0.306	0.2809	0.2304	0.2524	0.2056
Adam	0.6495	0.4381	0.4388	0.4207	0.5236	0.5136	0.5095	0.5062	0.5018	0.4913	0.4381	0.4388	0.4207	0.4529	0.4469	0.4357	0.5245	0.5228	0.5010	0.5167	0.5208	0.4983	0.4529	0.4469	0.4357
Qx Limb Muscle	0.8207	0.6879	0.6767	0.6417	0.6876	0.6862	0.6487	0.7026	0.6975	0.6556	0.6879	0.6767	0.6417	0.6823	0.6705	0.6448	0.7090	0.7230	0.6750	0.7149	0.7191	0.6598	0.6823	0.6750	0.6448
QS Heart	0.6643	0.4369	0.4330	0.4438	0.5003	0.5279	0.5125	0.5165	0.5323	0.4821	0.4642	0.4570	0.4461	0.4462	0.4495	0.4399	0.5132	0.5102	0.4754	0.4957	0.5101	0.4752	0.4406	0.4510	0.4463
Young	0.3955	0.3826	0.3757	0.3462	0.4507	0.4489	0.4280	0.4599	0.4689	0.4245	0.4051	0.3833	0.4007	0.4389	0.3869	0.3155	0.4326	0.4148	0.3987	0.4500	0.4620	0.3989	0.4143	0.4250	0.4155
Plasschaert	0.5319	0.2131	0.2172	0.2021	0.1280	0.1501	0.1072	0.1394	0.1241	0.1387	0.1945	0.1958	0.2192	0.1884	0.1910	0.1775	0.1240	0.1907	0.0366	0.0728	0.0318	0.0616	0.1714	0.2115	0.1973
Qx Spleen	0.6455	0.5228	0.5283	0.4681	0.42	0.5246	0.515	0.4815	0.5332	0.5309	0.4969	0.5259	0.4943	0.4618	0.4454	0.4314	0.5412	0.5244	0.4976	0.4962	0.5359	0.4699	0.4479	0.4486	0.4317
Chen	0.1510	0.3683	0.3413	0.3591	0.3436	0.3585	0.3751	0.4009	0.3627	0.3477	0.3817	0.3952	0.3486	0.3632	0.3693	0.3530	0.4497	0.4130	0.4172	0.4221	0.3459	0.3579	0.3623	0.3547	0.3570
Tosches turtle	0.3129	0.3013	0.3162	0.295	0.3291	0.3498	0.3485	0.3265	0.3419	0.3430	0.3150	0.3166	0.2866	0.3134	0.3175	0.3211	0.3237	0.3743	0.3580	0.3870	0.3900	0.2662	0.2950	0.2859	0.3184
Bach	0.5986	0.4181	0.4090	0.3825	0.4292	0.4142	0.3982	0.4151	0.4076	0.3763	0.4084	0.4111	0.3741	0.4021	0.3989	0.3625	0.4402	0.4137	0.3253	0.4322	0.4112	0.3815	0.4007	0.3990	0.3650
Average	0.5827	0.3775	0.3773	0.3575	0.4750	0.4823	0.4641	0.4786	0.4722	0.4573	0.3787	0.3805	0.3612	0.3769	0.3747	0.3557	0.5015	0.5033	0.4569	0.4861	0.4809	0.4587	0.3728	0.3756	0.3622

Datasets	scMGCA	UMAP											
		case25	case26	case27	case28	case29	case30	case31	case32	case33	case34	case35	case36
Pollen	0.7286	0.5418	0.5498	0.5179	0.7935	0.7888	0.7686	0.7912	0.7944	0.7737	0.5418	0.5498	0.5179
Camp-Brain	0.2201	0.1088	0.1071	0.1025	0.1877	0.2127	0.1805	0.1958	0.2146	0.1942	0.1088	0.1071	0.1025
Camp-Liver	0.6177	0.6489	0.6361	0.6364	0.6567	0.6728	0.6643	0.6845	0.6754	0.665	0.6489	0.6361	0.6364
QS Diaphragm	0.8458	0.3395	0.3305	0.3478	0.7004	0.7021	0.6749	0.6602	0.6580	0.6330	0.3395	0.3305	0.3478
QS Limb Muscle	0.8386	0.3685	0.3735	0.3698	0.7773	0.7938	0.7471	0.7460	0.7291	0.7145	0.3685	0.3735	0.3698
QS Trachea	0.7261	0.2740	0.2808	0.3034	0.4552	0.5194	0.4201	0.4418	0.4245	0.3920	0.2740	0.2808	0.3034
QS Lung	0.5414	0.2250	0.2744	0.2785	0.5403	0.5353	0.5263	0.5389	0.5356	0.5276	0.2250	0.2744	0.2785
Muraro	0.5584	0.4084	0.4133	0.3825	0.4210	0.4094	0.4284	0.3785	0.3681	0.3846	0.4084	0.4133	0.3825
Qx Bladder	0.8105	0.4380	0.4573	0.3909	0.7161	0.7381	0.7087	0.7607	0.7747	0.6931	0.4380	0.4573	0.3909
Klein	0.4544	0.0456	0.0437	0.0667	0.4247	0.4173	0.4061	0.4121	0.4123	0.3969	0.0456	0.0437	0.0667
Romanov	0.5416	0.2418	0.2545	0.2088	0.3136	0.3298	0.3073	0.3186	0.3217	0.3167	0.2418	0.2545	0.2088
Adam	0.6495	0.4507	0.4519	0.4365	0.5382	0.5412	0.5203	0.5294	0.5294	0.5018	0.4507	0.4519	0.4365
Qx Limb Muscle	0.8207	0.7097	0.6953	0.6365	0.7323	0.7405	0.7062	0.7416	0.7252	0.6768	0.7097	0.6953	0.6365
QS Heart	0.6643	0.4553	0.4576	0.4540	0.5182	0.5221	0.4520	0.5156	0.5188	0.4844	0.4611	0.4497	0.4562
Young	0.3955	0.3870	0.3986	0.4101	0.4584	0.3944	0.3776	0.3843	0.4312	0.4236	0.4149	0.4123	0.4084
Plasschaert	0.5319	0.1978	0.2018	0.1921	0.0909	0.0993	0.0986	0.0659	-0.0057	0.0445	0.1869	0.1794	0.1862
Qx Spleen	0.6455	0.4273	0.4325	0.4276	0.4992	0.4924	0.4622	0.4713	0.4515	0.4524	0.4159	0.4298	0.4013
Chen	0.1510	0.3567	0.3771	0.3265	0.3984	0.4233	0.4186	0.4316	0.3802	0.3941	0.3472	0.3492	0.3257
Tosches turtle	0.3129	0.2931	0.2886	0.2933	0.3590	0.3412	0.3720	0.3848	0.3782	0.3650	0.3125	0.3080	0.2852
Bach	0.5986	0.3950	0.3851	0.3615	0.4213	0.4183	0.3687	0.3979	0.3863	0.3896	0.3897	0.3866	0.3523
Average	0.5827	0.3656	0.3705	0.3572	0.5001	0.5046	0.4804	0.4925	0.4852	0.4712	0.3664	0.3692	0.3547

Supplementary Note 7: Visualization of scMGCA and three dimensionality reduction methods (PCA, UMAP and t-SNE) on 20 datasets

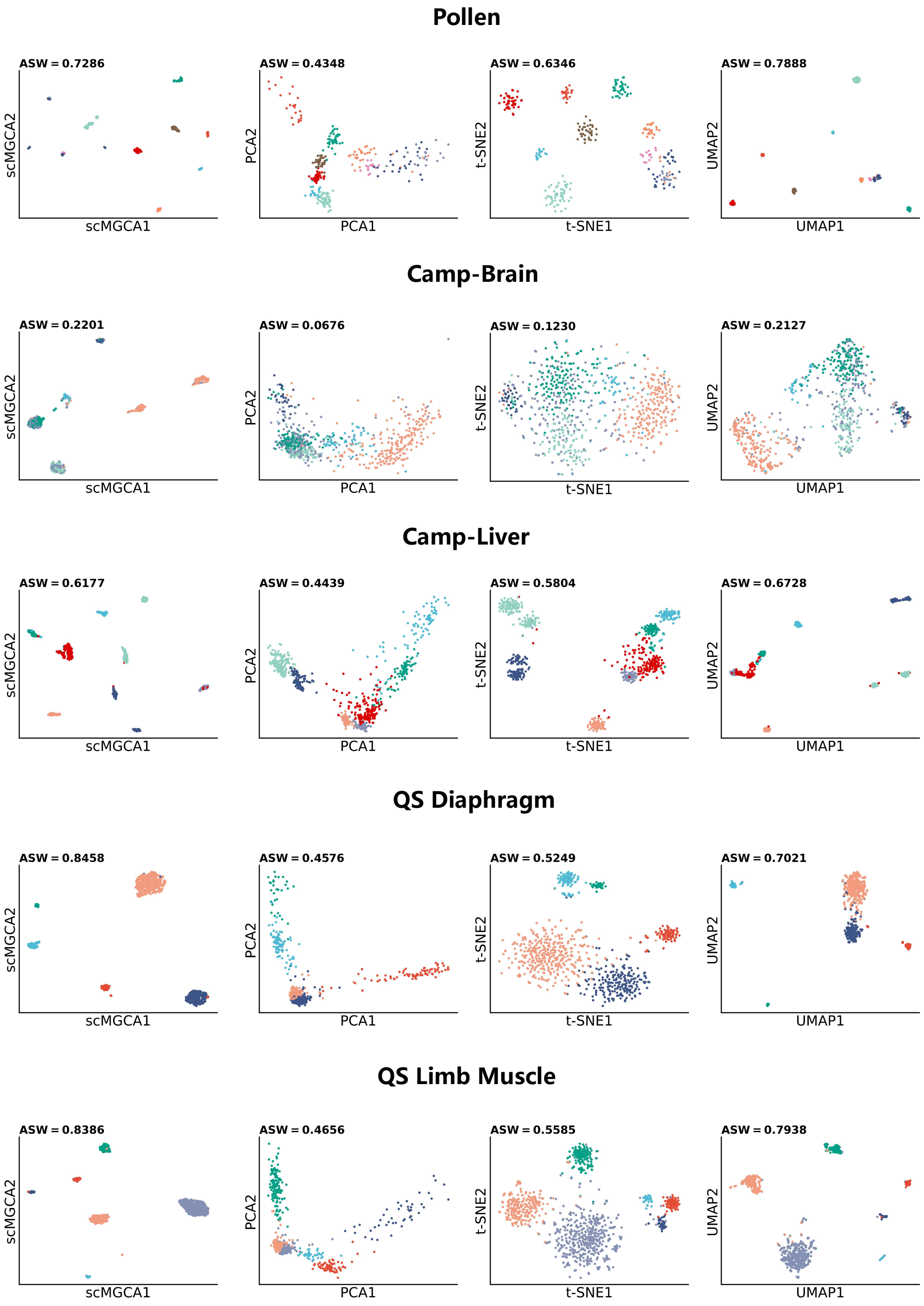

Supplementary Fig. 12. Comparison of dimensionality reduction and visualization between scMGCA and other three dimensionality reduction methods (PCA,t-SNE and UMAP) on the 'Pollen', 'Camp-Brain', 'Camp-Liver', 'QS Diaphgram', and 'QS Limb Muscle' dataset.

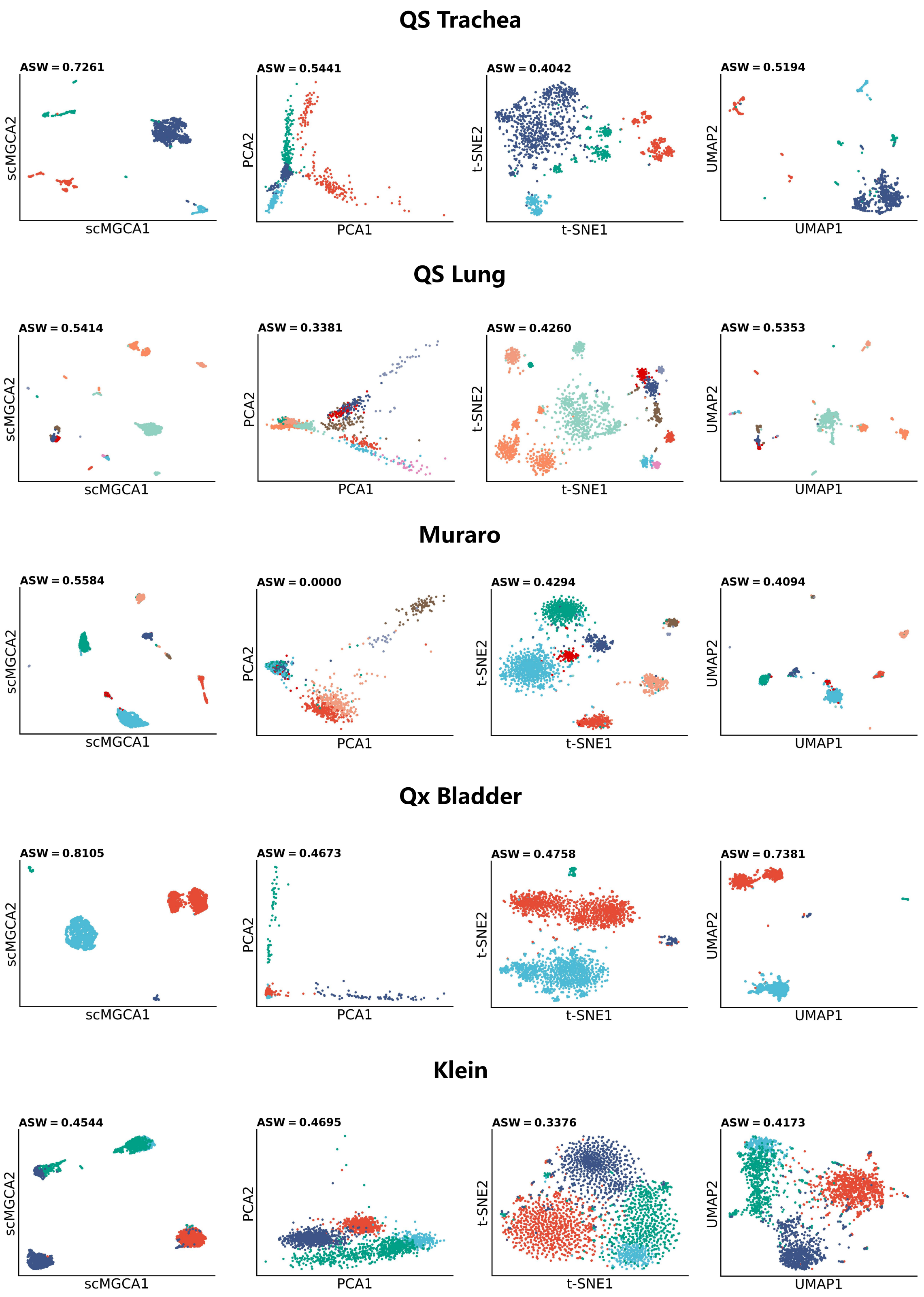

Supplementary Fig. 13. Comparison of dimensionality reduction and visualization between scMGCA and other three dimensionality reduction methods (PCA,t-SNE and UMAP) on the 'QS Trachea', 'QS Lung', 'Muraro', 'Qx Bladder', and 'Klein' dataset.

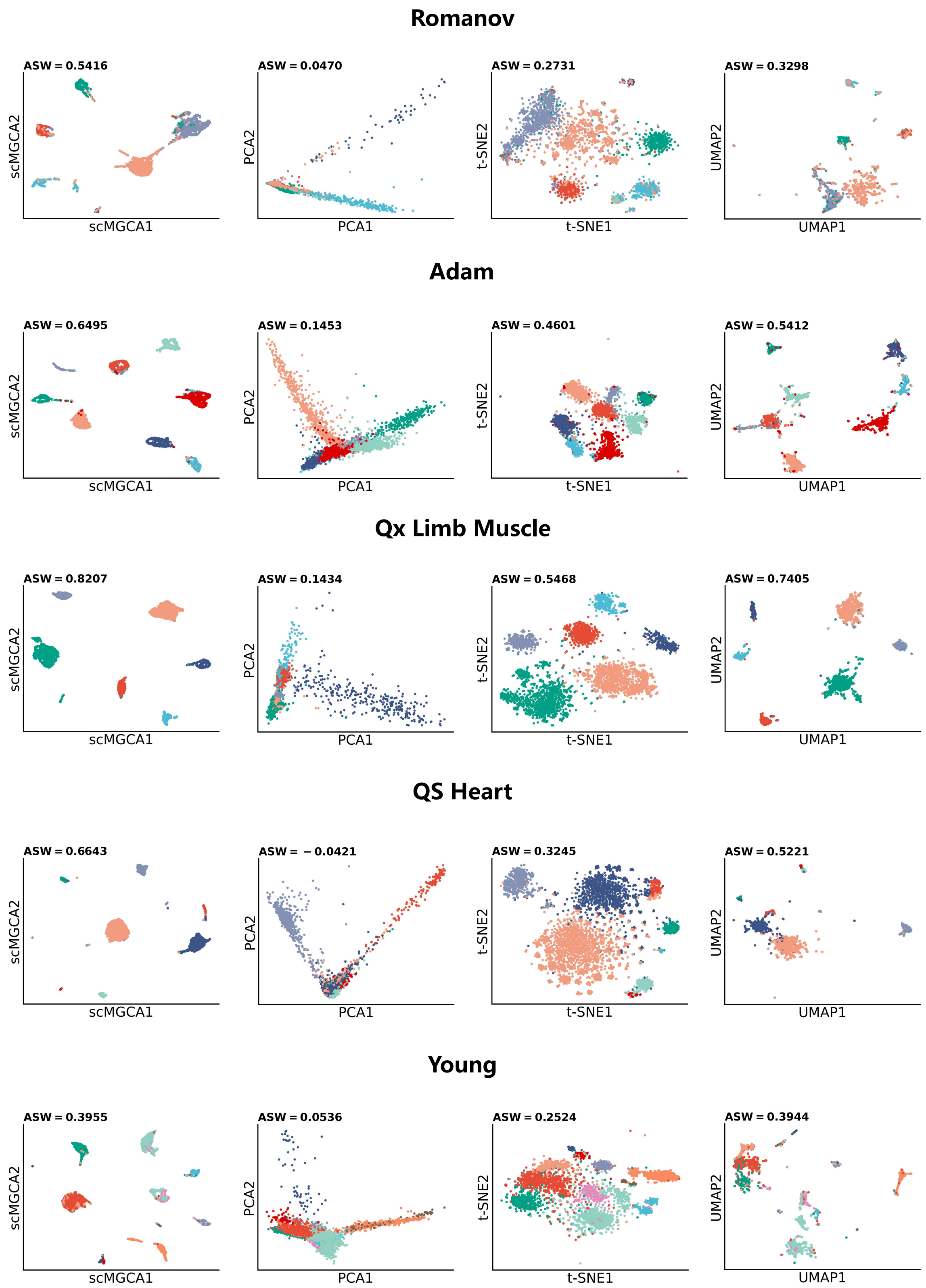

Supplementary Fig. 14. Comparison of dimensionality reduction and visualization between scMGCA and other three dimensionality reduction methods (PCA,t-SNE and UMAP) on the 'Romanov', 'Adam', 'Qx Limb Muscle', 'QS Heart', and 'Young' dataset.

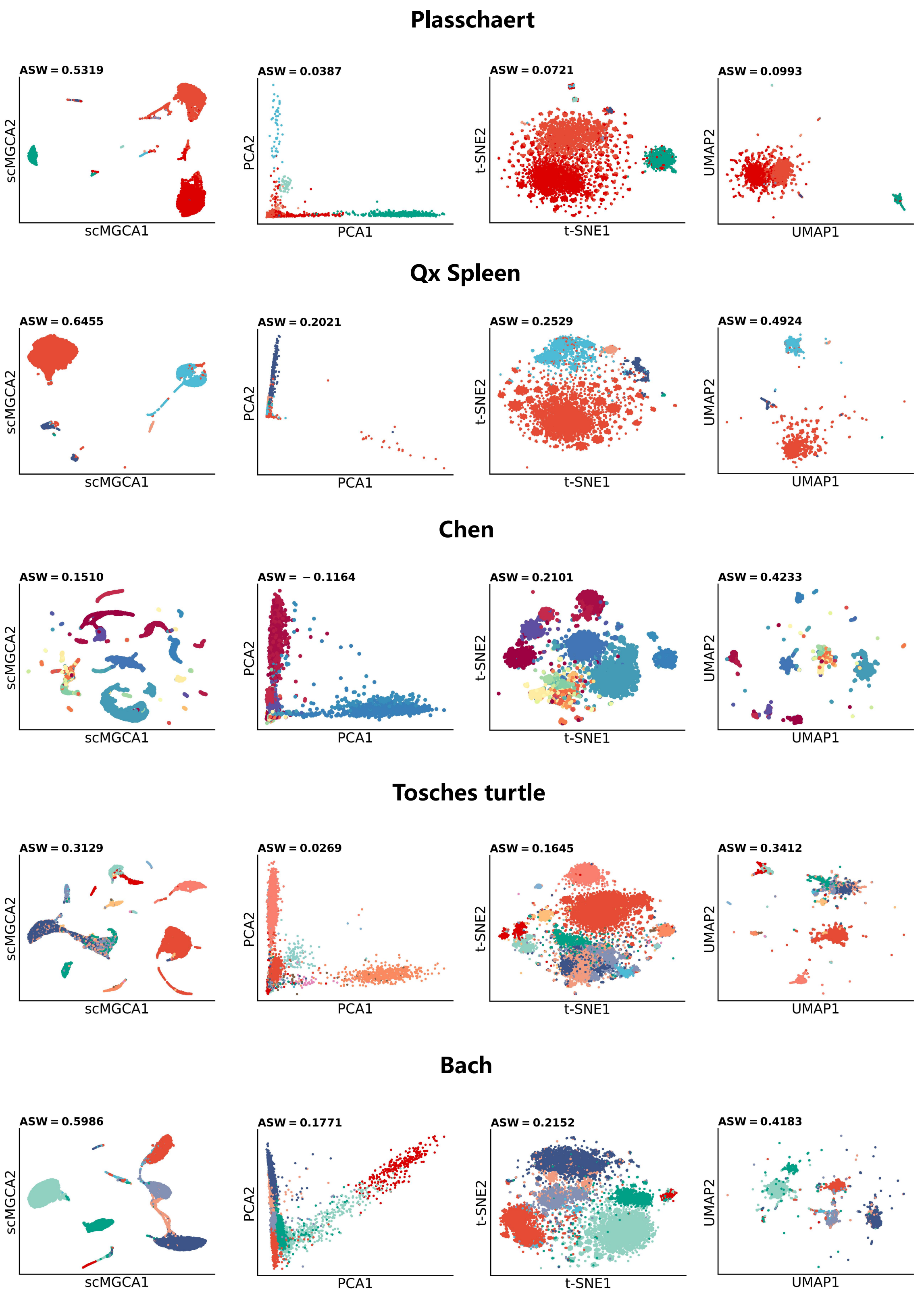

Supplementary Fig. 15. Comparison of dimensionality reduction and visualization between scMGCA and other three dimensionality reduction methods (PCA,t-SNE and UMAP) on the 'Plasschaert', 'Qx Spleen', 'Chen', 'Tosches turtle', and 'Bach' dataset.

Supplementary Note 8: Clustering algorithms and parameter settings for SCANPY and Seurat

SCANPY: The version of SCANPY used is 1.6.0. For preprocessing the data, we used "$scanpy.pp.normalize\_per\_cell$" function to normalize gene expression levels for each cell and "$scanpy.pp.log1p$" function to log-transform the data. Then, after normalization the data used "$scanpy.pp.highly\_variable\_genes$" function with (min_mean=0.0125, max_mean=3, min_disp=0.5, n_top_genes = 500) to select the top 500 highly variable genes. For clustering the data, we first used "$scanpy.tl.pca$" function with (svd_solver='arpack') and "$scanpy.pp.neighbors$" function with (n_neighbors=10, n_pcs=40) to find the neighborhood of the data, and then used "$scanpy.tl.leiden$" function with (resolution = 1, random_state = 0) for clustering.

Seurat: The version of Seurat we used is 4.0.0. For preprocessing data, we used "$log2$" and "$ScaleData$" function to normalize the data. After normalization, the data used "$RunPCA$" and "$FindNeighbors$" functions with (dims = 1:10) to find the neighborhood of the data, and then used "$FindClusters$" function with (resolution = 0.8, method = "matrix", algorithm = 1) for clustering.

Supplementary Note 9: Comparison of scMGCA with SCANPY and Seurat under multiple evaluation metrics

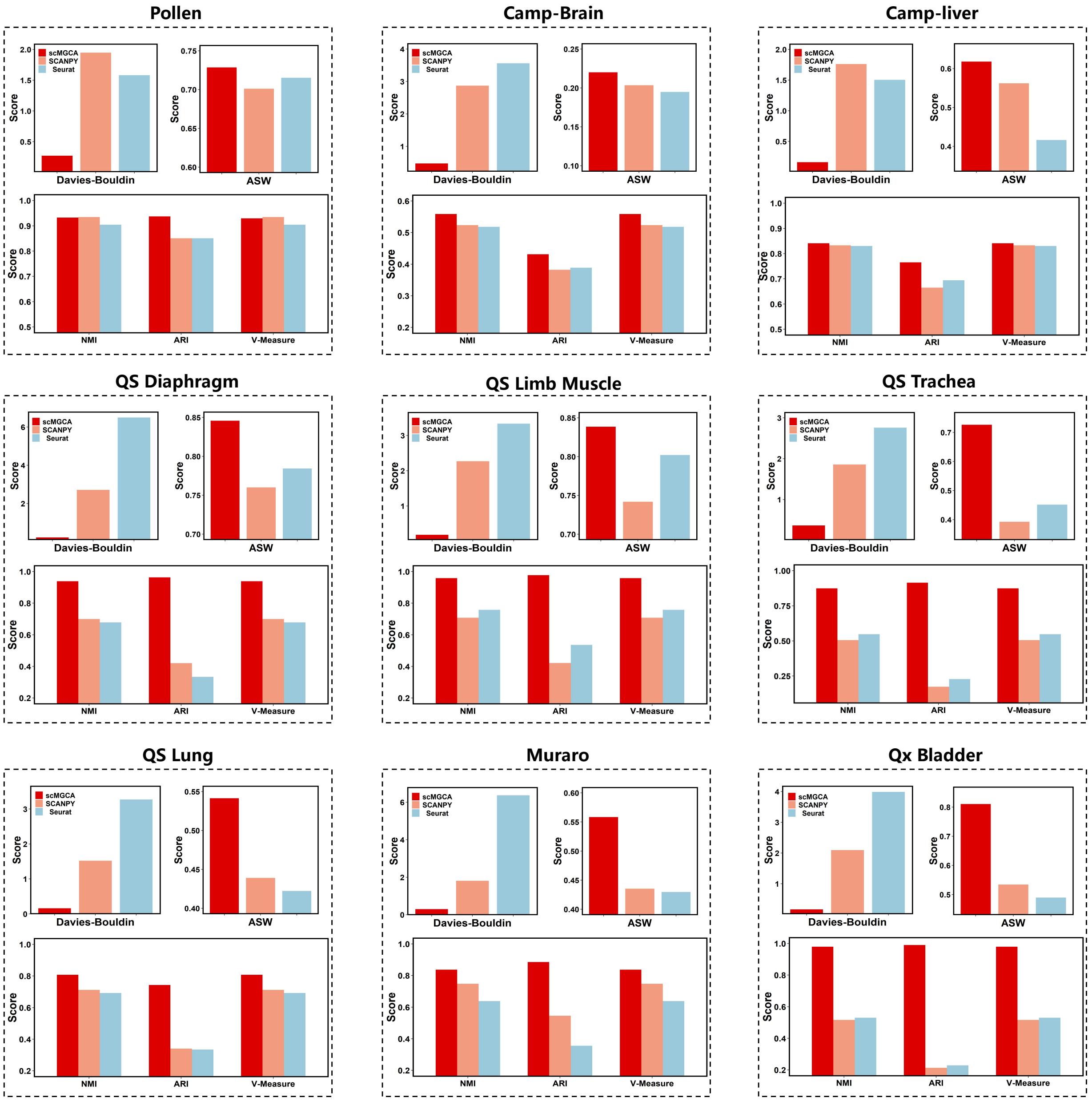

Supplementary Fig. 16. Comparison of scMGCA with SCANPY and Seurat on 5 metrics for 'Pollen', 'Camp-Brain', 'Camp-Liver', 'QS Diaphgram', 'QS Limb Muscle', 'QS Trachea', 'QS Lung', 'Muraro', and 'Qx Bladder' dataset. Source data are provided as a Source Data file.

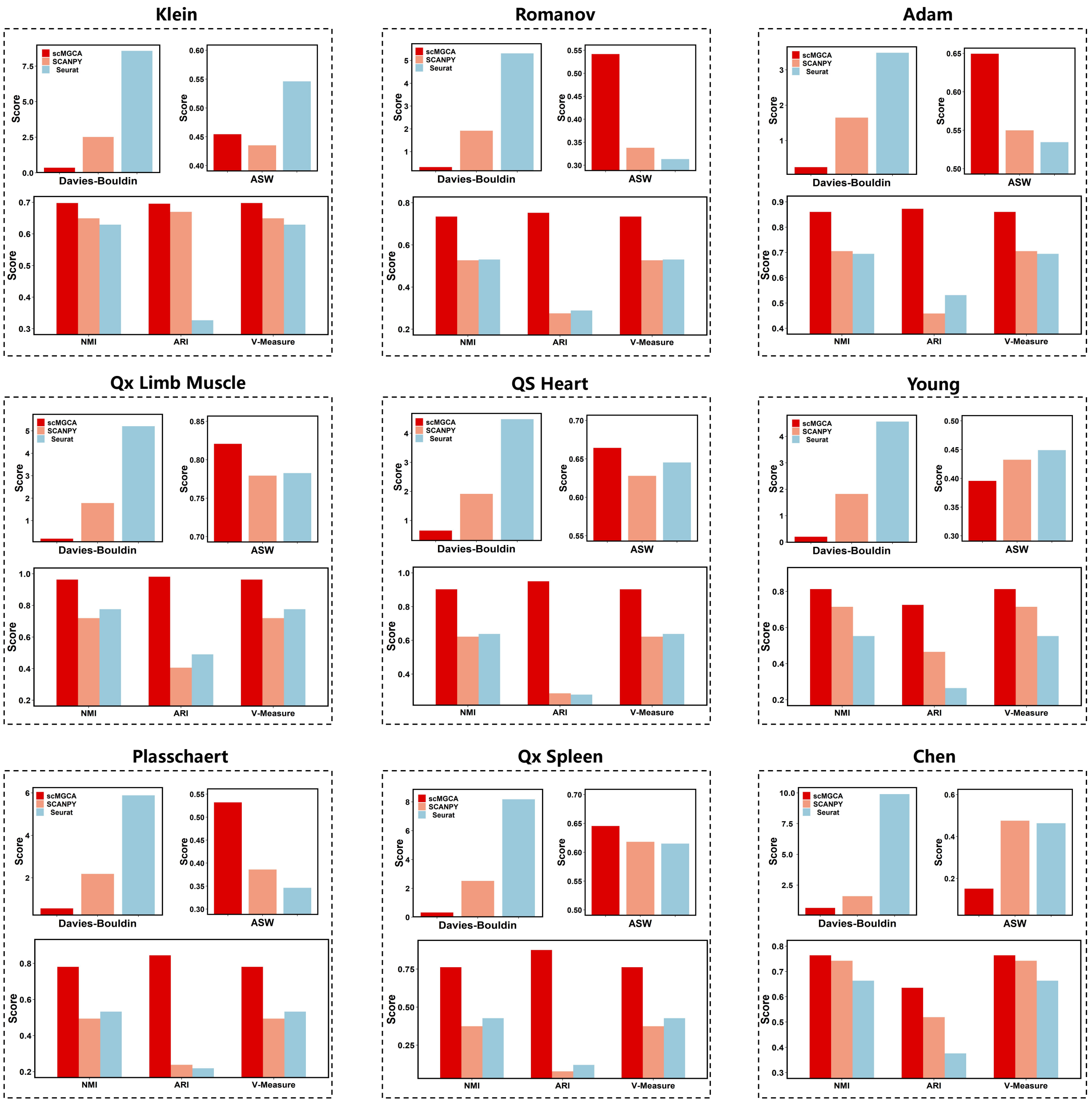

Supplementary Fig. 17. Comparison of scMGCA with SCANPY and Seurat on 5 metrics for 'Klein', 'Romanov', 'Adam', 'Qx Limb Muscle', 'QS Heart', 'Young', 'Plasschaert', 'Qx Spleen', and 'Chen' dataset. Source data are provided as a Source Data file.

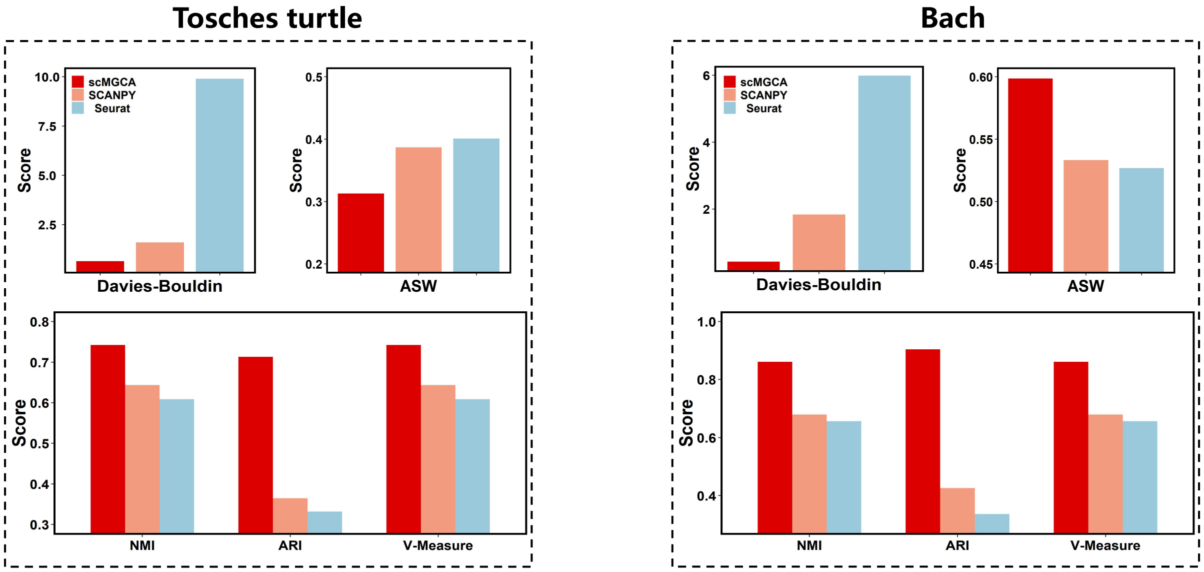

Supplementary Fig. 18. Comparison of scMGCA with SCANPY and Seurat on 5 metrics for ‘Tosches turtle’, and ‘Bach’ dataset. Source data are provided as a Source Data file.

Supplementary Note 10: A comparison of the initialization performance of K-means and spectral clustering and the conditions under which these two initialization methods can be effective

The cell-PPMI graph of some single-cell datasets can preserve more cell topological information, so the information contained in the cell-PPMI graph will be richer than that in the latent embedding representation obtained only by pre-training. Therefore, using spectral clustering to initialize the the clustering centers can result in a better final clustering performance than K-means for these datasets. On the contrary, when the cell-PPMI graph of other single-cell datasets preserves less information than others, the latent embedding representation obtained by pre-training will supplement some gene expression information in topology information, and it is preferable to use K-means to initialize the cluster centers in this case.

To demonstrate this point, we first construct a truth cell graph $\mathbf{T}$ using the ground-truth labels underlying the scRNA-seq data, which can be defined as follows:

$$T_{ij} = \begin{cases} 1, & l_j = l_i \\ 0, & l_j \neq l_i \end{cases} \quad \textbf{(1)}$$

where i and j represent the i-th cell and j-th cell, and l_i and l_j represent the true labels of the i-th cell and the j-th cell, respectively. According to Eq. (1), it is known that in the truth cell graph $\mathbf{T}$, only cells belonging to the same cluster are edge-connected; and there is not any edge connection between the cells from different clusters. Therefore, $\mathbf{T}$ provides the real and valid topological information in single-cell data, and can be used as the “true labels" of topological information. Then, we calculate the occupancy (t) of the real topological information by measuring the cell-PPMI graph $\mathbf{A}$ and the truth cell graph $\mathbf{T}$, defined as follows:

$$t = \frac{\text{sum}(\mathbf{A} \odot \mathbf{T})}{\text{sum}(\mathbf{A})} = \frac{\sum_{i=1}^{n}\sum_{j=1}^{n}(A_{ij}T_{ij})}{\sum_{i=1}^{n}\sum_{j=1}^{n}A_{ij}} \times 100\% \quad \textbf{(2)}$$

where $\odot$ is the Hadamard product. The cell connection edges in $\mathbf{A}$ that co-occur with the true topology $\mathbf{T}$ are retained by $\mathbf{A} \odot \mathbf{T}$, and the proportion of the sum of the weights of these valid edges to the sum of all weights in $\mathbf{A}$ is calculated to measure how much effective topology the cell-PPMI graph $\mathbf{A}$ can retain. On this basis, we calculated the effective topological occupancy (t) of the cell-PPMI graph $\mathbf{A}$ for each dataset, as well as their clustering performance (NMI) of initialized cluster centers using K-means and spectral clustering across the 20 datasets, as summarized in Supplementary Table 4.

From the table, we find that the difference in clustering performance between kmeans and spectral clustering as initialization methods is not very large on most of the datasets while there is a significant difference on a few datasets. For the "Camp-Liver", "QS Trachea" and "Qx Bladder" datasets (blue), the K-means model as an initialized clustering center is significantly better than spectral clustering, and the effective topological occupancy (t) of the cell-PPMI graph for these three datasets is below 90%. On the contrary, spectral clustering is significantly better than K-means as a method for initializing cluster centers across the ‘Adam’, ‘Qx Limb Muscle’, ‘QS Heart’, ‘Plasschaert’ and ‘Qx Spleen’ datasets (red), where the t of the cell-PPMI graph of these datasets is higher than 90%. For the rest, no matter whether K-means or spectral clustering is chosen as the initialized clustering center, they have similar results. In addition, on the whole, the effective topological occupancies (t) of the cell-PPMI graph of the datasets where spectral clustering is better than K-means are generally higher than those in the opposite cases (datasets). These cases verify our previous hypothesis that when the true topological occupancy of the cell-PPMI graph

constructed from the dataset is high, it preserves more topological information than the information obtained through pre-training in the latent representation, therefore spectral clustering would be better than K-means as the initialization method, while, when the true topological occupancy of the cell-PPMI graph constructed from the dataset is low, K-means becomes a better choice than spectral clustering.

Supplementary Table 22. Comparison of clustering performance and t with different initialization methods.

Methods	Pollen	Camp-Brain	Camp-Liver	QS Diaphragm	QS Limb Muscle
K-means	0.9263	0.5352	0.9224	0.9291	0.9586
Spectral	0.8971	0.5585	0.7730	0.9263	0.9586
t	84.97%	92.35%	62.37%	89.64%	92.60%
Methods	QS Trachea	QS Lung	Muraro	Qx Bladder	Klein
K-means	0.8736	0.8402	0.8255	0.9595	0.6971
Spectral	0.7369	0.8068	0.8385	0.8033	0.6694
t	87.15%	93.17%	92.05%	85.80%	82.84%
Methods	Romanov	Adam	Qx Limb Muscle	QS Heart	Young
K-means	0.7236	0.7894	0.8633	0.7999	0.7999
Spectral	0.6909	0.8593	0.9459	0.9020	0.8109
t	82.70%	92.50%	97.69%	96.38%	90.25%
Methods	Plasschaert	Qx Spleen	Chen	Tosches turtle	Bach
K-means	0.6962	0.7213	0.7241	0.7046	0.8195
Spectral	0.7732	0.7840	0.7640	0.7320	0.8241
t	95.22%	98.12%	89.02%	88.45%	94.98%

Supplementary Note 11: Parameter analysis

Clustering performance of scMGCA when perturbing the number of clusters

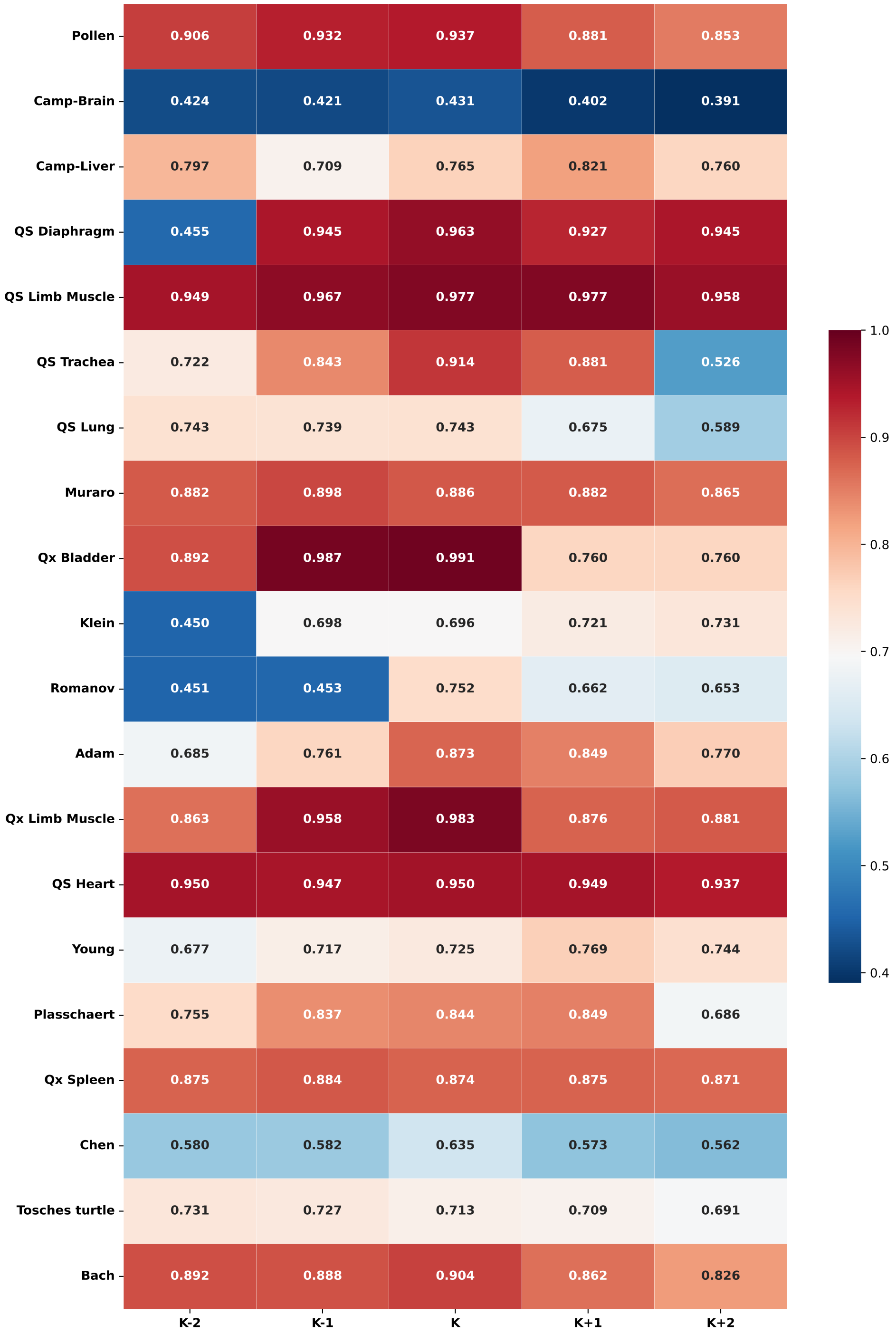

Supplementary Fig. 19. The heatmap of ARI for different numbers of cell clusters in scMGCA.

Clustering performance of scMGCA with different loss weight assignments

In our study, the total loss function trained and optimized by scMGCA is defined as follows:

$$\mathcal{L} = \gamma_1 \mathcal{L}_r + \gamma_2 \mathcal{L}_m + \gamma_3 \mathcal{L}_c \tag{3}$$

where $\mathcal{L}_r$ represents the reconstruction loss between the original cell graph (cell-PPMI graph) and the reconstructed cell graph obtained by the inner product decoder; $\mathcal{L}_m$ is the multinomial-based loss that simulates the distribution of the single-cell data by the multinomial-based decoder; $\mathcal{L}_c$ denotes the KL divergence-based clustering loss simultaneously optimized with other losses; γ_1, γ_2 and γ_3 are weight coefficients assigned to each loss.

It is extremely difficult to set the weights of different losses in deep learning, and most research is based on the experience of the researchers themselves or on the papers of earlier studies as a guide. Indeed, in this paper, the weight coefficients were chosen (after some preliminary experiments) to give robust results. We collected and summarized different weight coefficients from previous studies (2). On this basis, γ_1, γ_2, and γ_3 were selected from [0.3, 0.6, 0.9], [1, 1.5, 2], and [1, 1.5, 2] respectively. Then, to provide comprehensive performance evaluations, we enumerated them to obtain 27 distinct loss weight assignments and compared the clustering performance in those scenarios (Supplementary Fig. 20). From Supplementary Fig. 20, we can observe that variation in the loss weight over a certain range has little effect on the clustering performance of scMGCA across the majority of datasets. Of these three datasets, 'QS Trachea', 'QS Lung' and 'Chen' are sensitive to the weight coefficients of the loss function with noticeable performance fluctuations. Overall, when γ_1, γ_2, and γ_3 are set to [0.3, 1, 1.5], the average NMI and ARI values are the best among those 27 cases.

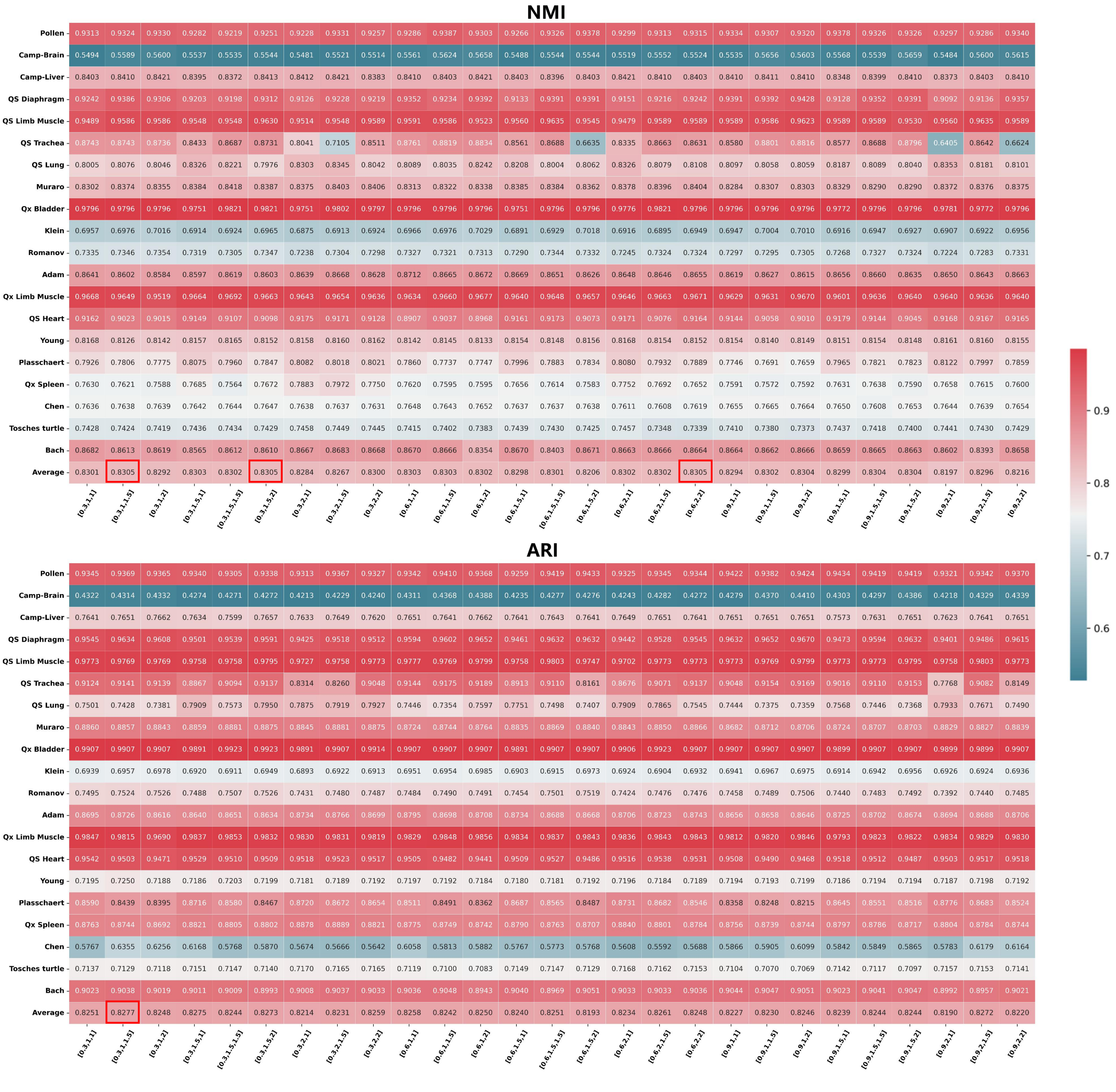

Supplementary Fig. 20. The clustering performance of different loss weight assignments on 20 single-cell datasets evaluated using NMI and ARI evaluation metrics. Source data are provided as a Source Data file.

Clustering performance of scMGCA with different parameters in cell-PPMI graph

The cell graph (cell-PPMI graph) in scMGCA is mainly divided into three parts: 1. Convert the gene expression matrix into an unweighted KNN graph through the KNN algorithm, and preliminarily determine the neighborhood of each cell; 2. Capture the transition probabilities between different cells from the KNN graph structural information using a random surfing model and then aggregate the adjacent cells; 3. The transition probability matrix is summed within a finite transfer step to obtain the probability matrix, and its positive point mutual information (PPMI) matrix is calculated to characterize similar cells, and finally the cell graph is further enhanced by assigning co-occurrence attention mechanisms.

To clearly demonstrate the performance of different parameters in the cell graph construction, we first supplemented the experiments by adjusting the three parameters in the construction of cell graph. The first parameter is the initial number of neighbors (k) when building the KNN graph, which we choose from [10, 15, 20]. The second parameter is the distance function (m) to determine the neighbors of each cell when building the KNN graph, where we have four distance functions to choose including the Euclidean distance, the cosine distance, the Pearson correlation coefficient, and the Minkowski distance. The last parameter is the number of transfer steps (s) in the random surfing to determine the scope of search and aggregation, which we choose from [1, 2, 3] for our analysis. To provide comprehensive comparisons, we compare the clustering performance of different combinations of these three parameters (36 cases) under the evaluation metrics NMI and ARI Supplementary Fig. 21. The experimental results showed that the average NMI and ARI for the 20 datasets were ranged from 0.794 to 0.831 and from 0.748 to 0.828 respectively. Indeed, we can observe that the clustering effect is the best at "k=15, m=Euclidean, s=2", with the highest average NMI and ARI. Therefore, we adopt this parameter setting when constructing the cell-PPMI graph.

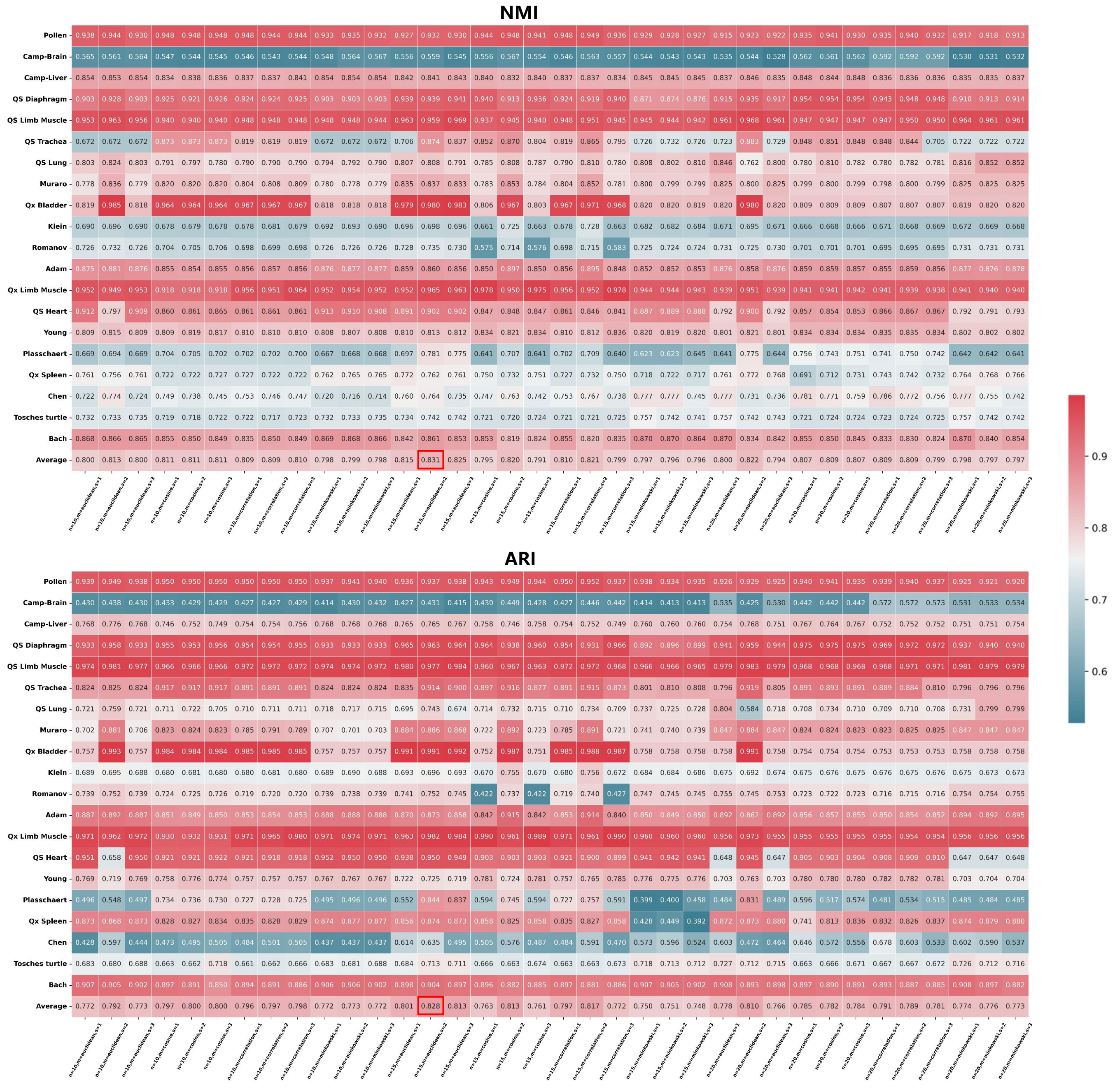

Supplementary Fig. 21. The clustering performance of different parameters for building the cell-PPMI graph on 20 single-cell datasets evaluated by NMI and ARI evaluation. Source data are provided as a Source Data file.

Clustering performance of scMGCA with different network layers

For the number of consecutive layers of GCN, we have benchmarked four cases as one, two, three, and four layers with the number of neuron nodes [15], [128, 15], [256, 128, 15] and [512, 256, 128, 15], respectively, which are denoted as scMGCA1, scMGCA2 (our proposed scMGCA), scMGCA3, and scMGCA4 respectively. We compared the clustering performance of these four different frameworks on 20 single-cell datasets with NMI and ARI as evaluation metrics Supplementary Fig. 22. It can be clearly observed that the NMI and ARI of scMGCA2 (scMGCA) outperform the other frameworks on most datasets, and the average clustering performance on all 20 datasets is also the best. For the ‘QS Trachea’, ‘Qx Bladder’ and ‘Romanov’ datasets, the clustering performance of the two-layer GCN is significantly better than the other layers. The ARI score on the ‘Chen’ dataset is greatly affected by the number of layers of GCN. scMGCA4 has the worst clustering performance among all the comparison frameworks. For the reasons, it could be possible that the network structure with 3 or more layers could be over-complicated due to the excessive number of hidden layer nodes. It is more likely to fall into local minima during the back-propagation process with gradient vanishing, and the learning speed will also be too slow.

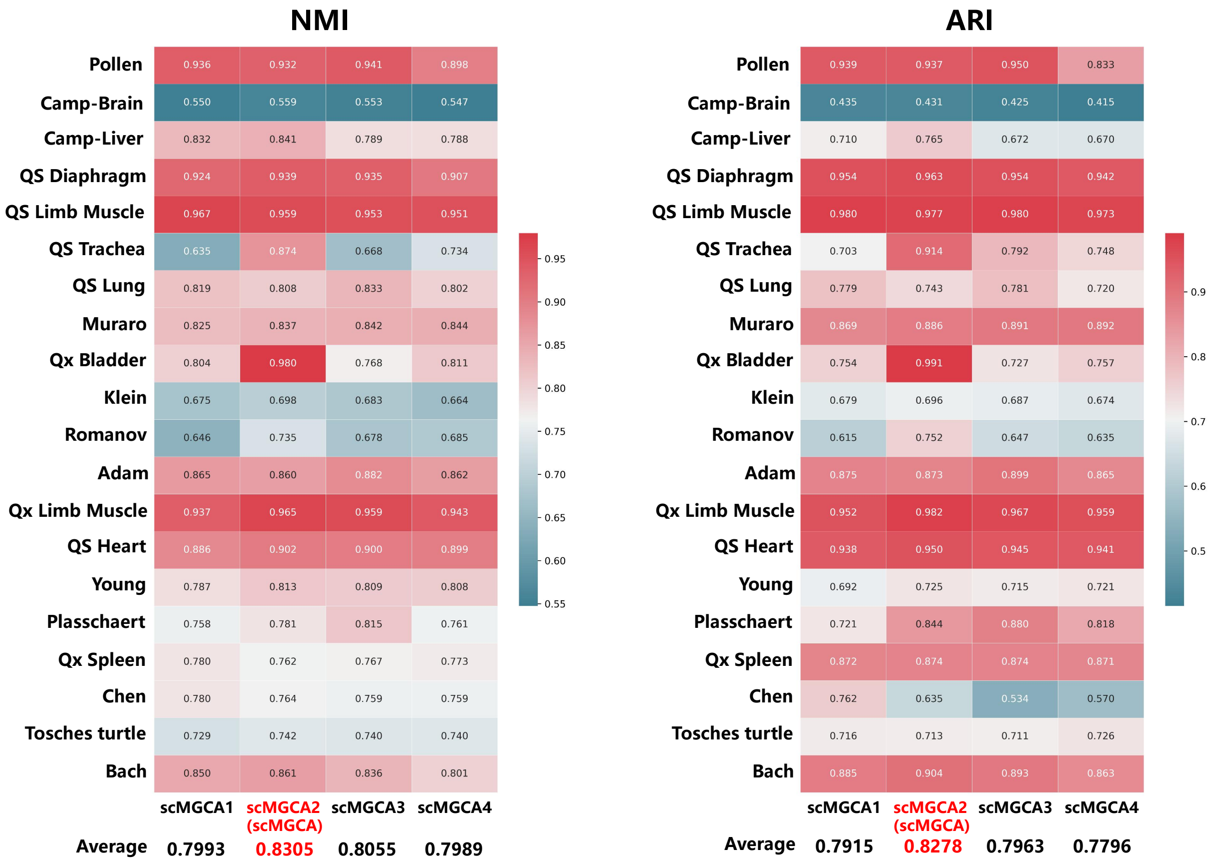

Supplementary Fig. 22. The clustering performance of four different frameworks on 20 single-cell datasets was evaluated using NMI and ARI as evaluation metrics.

Clustering performance of scMGCA with different network frameworks

We have designed a parallel framework-based scMGCA inspired by SEDR (3) called scMGCA-p, as shown in Supplementary Fig. 23 that considers count data and PPMI matrix in parallel. In scMGCA-p, we parallelize the multinomial-based autoencoder and the graph convolution autoencoder, allowing for the parallel inputs of the gene expression matrix and cell-PPMI graph. Indeed, the multinomial-based autoencoder simulates the dropout event of the gene expression matrix to obtain the latent representation z_1 while the graph convolutional autoencoder integrates the gene expression matrix and cell-PPMI graph capturing the cell topology to obtain the latent representation z_2. Finally, z_1 and z_2 are concatenated and optimized with KL divergence-based clustering loss to obtain the final clustering result. The experimental results comparing scMGCA and scMGCA-p are summarized in Supplementary Fig. 24, showing the clustering performance of the two architectures on 20 single-cell datasets with NMI and ARI as evaluation metrics. From the results, we can observe that the clustering performance of scMGCA-p is lower than that of scMGCA and then we compared the results of scMGCA and scMGCA-p using the pairwise t-test, with p-values of 5.4×10^{-3} (ARI) and 1.1×10^{-2} (NMI), indicating a significant difference.

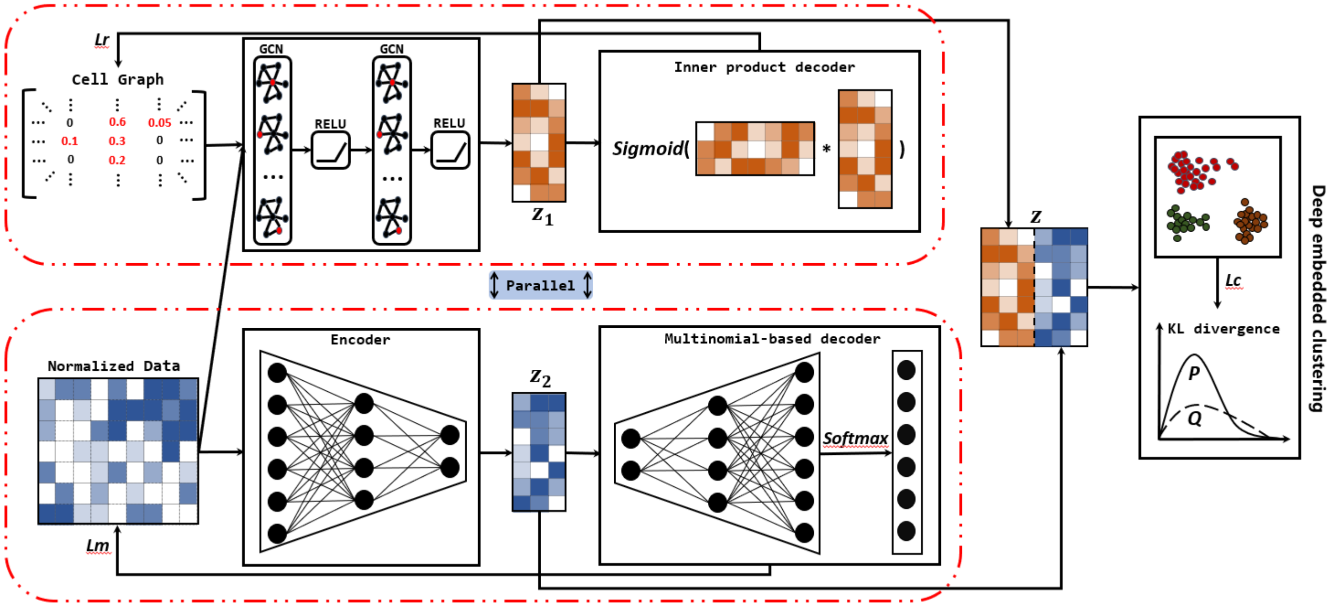

Supplementary Fig. 23. The network architecture of the scMGCA-p algorithm.

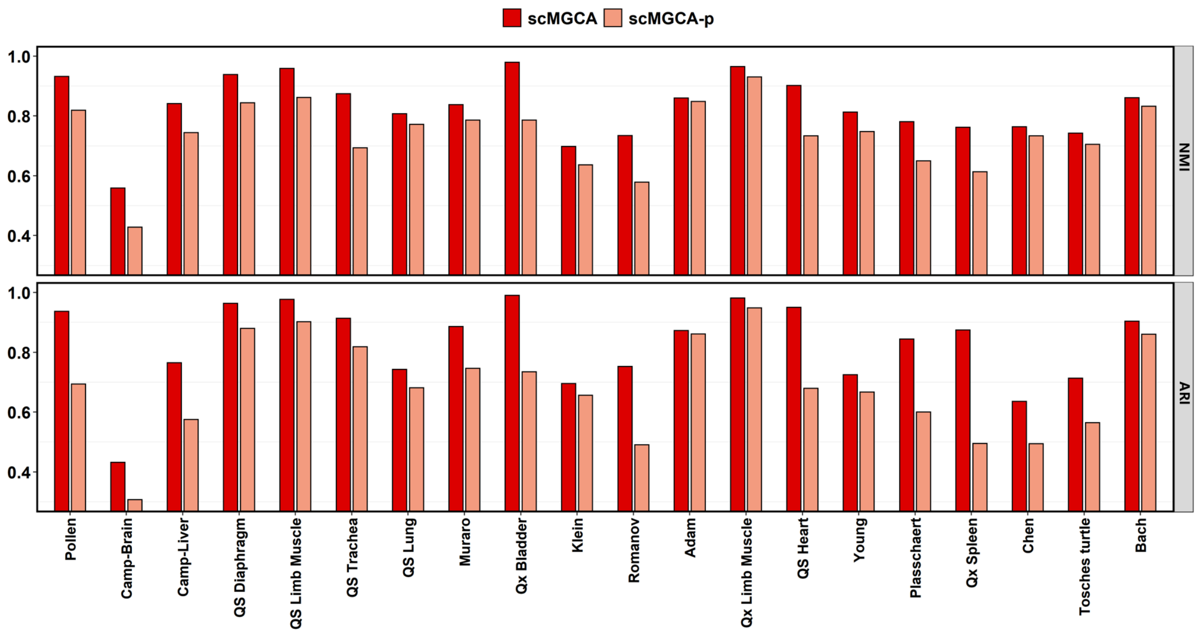

Supplementary Fig. 24. The comparison of the clustering performances of scMGCA and scMGCA-p on 20 single-cell datasets using NMI and ARI as evaluation metrics. Source data are provided as a Source Data file.

Ablation study

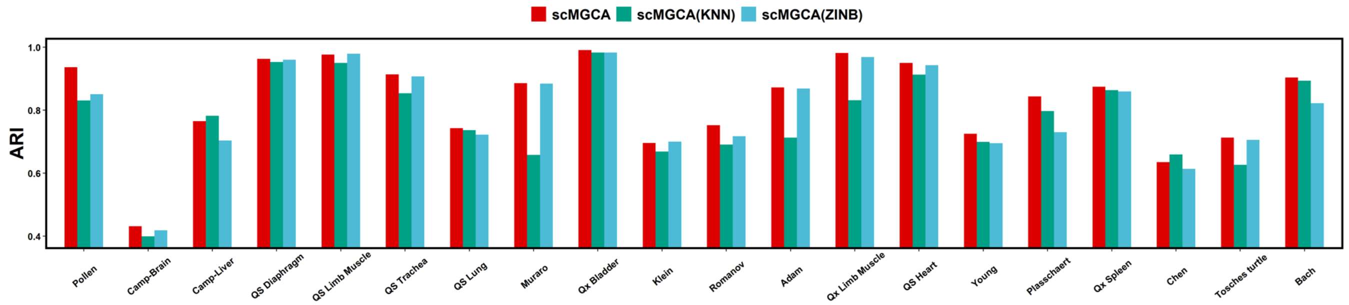

Supplementary Fig. 25. The ablation study for cell graph and multinomial-based distribution decoder. Source data are provided as a Source Data file.

Supplementary Note 12: Comparison of the cell-PPMI graph and the KNN graph

For constructing a cell graph through KNN, it is to calculate the distance between each cell and other cells, and select the top k closest cells as neighbors, assign higher weight edge connections, and have no edge connections with the other remaining cells (weight is 0). Indeed, the KNN graph, is straightforward in its construction. However, according to some previous studies (2, 4), the KNN graph, which directly assigns weights only by the distance between two nodes, is more suitable for completely random networks. For the single-cell network composed of cells with homogeneity and consistency, the KNN graph may not be compatible with the single-cell network properties from different platforms. The reason could be that the KNN graph only focuses on the relationship between two cells and does not perceive the underlying information of surrounding cells (e.g. cell clusters), which often also plays an important role in the single-cell network.

The cell-PPMI graph in scMGCA can address the shortcomings of KNN graph and can also strengthen the aggregated cell neighborhood. The construction of the cell-PPMI graph is mainly divided into three parts. First, the cell-PPMI graph starts from an unweighted KNN graph $\mathbf{K}$, which is defined as follows:

$$K_{ij} = \begin{cases} 1, & j \in \mathcal{N}_k(i) \\ 0, & j \notin \mathcal{N}_k(i) \end{cases} \tag{4}$$

where $\mathcal{N}_k(i)$ represents the neighborhood containing the nearest k nodes with the i-th node as the center. This step mainly uses the KNN algorithm to initialize the neighborhood of each cell for cell-PPMI graph. Then, we use the random surfing algorithm to process $\mathbf{K}$ to obtain the transition matrix $\mathcal{P}^s$, which is defined as follows:

$$\begin{aligned} \mathcal{P}^s &= \alpha \cdot \mathcal{P}^{s-1}\mathbf{K} + (1-\alpha)\mathcal{P}^0 \\ &= \alpha^s \cdot \mathcal{P}^0\mathbf{K}^s + (1-\alpha)\sum_{t=1}^{s} \alpha^{s-t} \cdot \mathcal{P}^0\mathbf{K}^{s-t} \end{aligned} \tag{5}$$

where s is the transfer step ($s \leq 3$), and $\mathcal{P}^0$ is the initial transition matrix equal to the identity matrix ($\mathcal{P}^0 = \mathcal{I}$). The probability of returning to the initial vertex and restarting the process is set to $(1-\alpha)$. $\mathcal{P}^s_{ij}$ represents the probability of the i-th node reaching the j-th node after s steps of transitions. By transferring and walking in the network, the random surfing algorithm obtains the local and global topology among neighboring cells. Finally, we sum all the transition matrices after s steps to get the probability matrix $\mathbf{U} = \sum_{s=1}^{S} \mathcal{P}^s$, and use PPMI to further augment the graph, which is defined as follows:

$$\begin{aligned} PPMI(x,y) &= \max(PMI(x,y), 0) \\ &= \max(\log\left(\frac{p(x,y) \cdot |\mathbf{S}|}{p(x)p(y)}\right), 0) \end{aligned} \tag{6}$$

where $|\mathbf{S}| = \sum_x \sum_y p(x,y)$, x, and y represent different nodes; $p(x)$ and $p(y)$ denote the probability of x and y, respectively; $p(x,y)$ represents the co-occurrence probability of x and y. These probabilities are all obtained from $\mathbf{U}$, so the final cell-PPMI graph $\mathbf{A}$ is defined as follows:

$$\mathbf{A} = \max\left(\log\left(\frac{\mathbf{U}\Theta}{\text{row}(\mathbf{U})\text{col}(\mathbf{U})}\right), 0\right) \tag{7}$$

where Θ denotes the sum of all elements in $\mathbf{U}$. $\mathrm{row}(\mathbf{U})$ and $\mathrm{col}(\mathbf{U})$ are the column vector of the sum of each row of $\mathbf{U}$ and the row vector of the sum of each column of $\mathbf{U}$, respectively. To verify our point, we first demonstrate that the cell-PPMI graph can uncover underlying information in the cell network that cannot be perceived by the KNN method in the analytical manner. Mathematically, the KNN graph directly assigns weights by the distance between two cells, and it is easy to ignore potential neighbor information (such as neighbors' neighbors) due to the selection of the number of neighbors and the calculation of the distance. Suppose that in the KNN graph cell i has an edge with cell j, and cell j has an edge with cell m, but i has no edge with m and m is a potential neighbor of i. We now demonstrate that the cell-PPMI graph is capable of discovering cell k. According to equation 5, since $(1-\alpha)\mathcal{P}^0$ only changes the value of the diagonal of $\mathcal{P}^s$, it does not change the matrix structure of $\mathcal{P}^s$, and does not affect matrix multiplication, so we can conclude that $\mathcal{P}^s$ is equivalent to $\mathbf{K}^s$, that is:

$$\mathcal{P}^s \Longleftrightarrow \mathcal{P}^0(\underbrace{\mathbf{K}\cdot\mathbf{K}\cdots\mathbf{K}}_{s}) \Longleftrightarrow \mathbf{K}^s \quad \textbf{(8)}$$

According to the previous assumption, both K_{ij} and K_{jm} are equal to 1 and K_{im} is equal to 0. However, when K is exponentiated, the potential neighbor cell m of cell i is found, as follows:

$$\begin{aligned}(\mathbf{K}\cdot\mathbf{K})_{im} &= \sum_{c=1}^{n} K_{ic}\cdot K_{cm} \\ &= \sum_{\substack{c=1 \\ c\neq j}}^{n} K_{ic}\cdot K_{cm} + K_{ij}\cdot K_{jm} \\ &\geqslant K_{ij}\cdot K_{jm} > 0\end{aligned} \quad \textbf{(9)}$$

where n is the total number of cells. According to equation 9, it can be induced that the random surfing algorithm finds the information of cell i and cell m that cannot be found in the KNN graph through their common neighbor cell j when transferring and walking.

We now deduce that the cell-PPMI graph can aggregate neighboring cells to augment the cell graph. Equation 9 can also be rewritten as follows:

$$\begin{aligned}(\mathbf{K}\cdot\mathbf{K})_{ij} &= \sum_{c=1}^{n} K_{ic}\cdot K_{cj} \\ &= \sum_{\substack{c=1 \\ c\neq a}}^{n} K_{ic}\cdot K_{cj} + \sum_{\substack{a\in\mathcal{N}_k(i) \\ a\in\mathcal{N}_k(j)}}^{n} K_{ia}\cdot K_{aj} \\ &\geqslant \sum_{\substack{a\in\mathcal{N}_k(i) \\ a\in\mathcal{N}_k(j)}}^{n} K_{ia}\cdot K_{aj}\end{aligned} \quad \textbf{(10)}$$

where cell a is the common neighbor of cell i and cell j. According to equation 10, it is proved that the weight between cell i and cell j in the random surfing algorithm is affected by the number of common neighbor cells. In other words, the random surfing algorithm amplifies the weights between closely related cells, aggregates neighboring cells, and propagates this information to the probability matrix $\mathbf{U}$. Furthermore, according to equation 7, PPMI can stretch the low-weight part by logarithmic

transformation and remove it to augment the cell graph.

Apart from this, we also provide the comparison results for scMGCA, scMGCA(SNN), scMGCA(wKNN1) and scMGCA(wKNN2) on those 20 scRNA-seq data, as depicted in Supplementary Fig. 26. scMGCA(SNN), scMGCA(wKNN1) and scMGCA(wKNN2) employed SNN graph constructed in Seurat (5), the weighted KNN graph in which the weights between cells are specified as the min-max normalized Euclidean distance subtracted from 1 and the weighted KNN graph constructed in UMAP (6), respectively. From the results, the clustering performance of scMGCA outperforms other methods on most datasets.

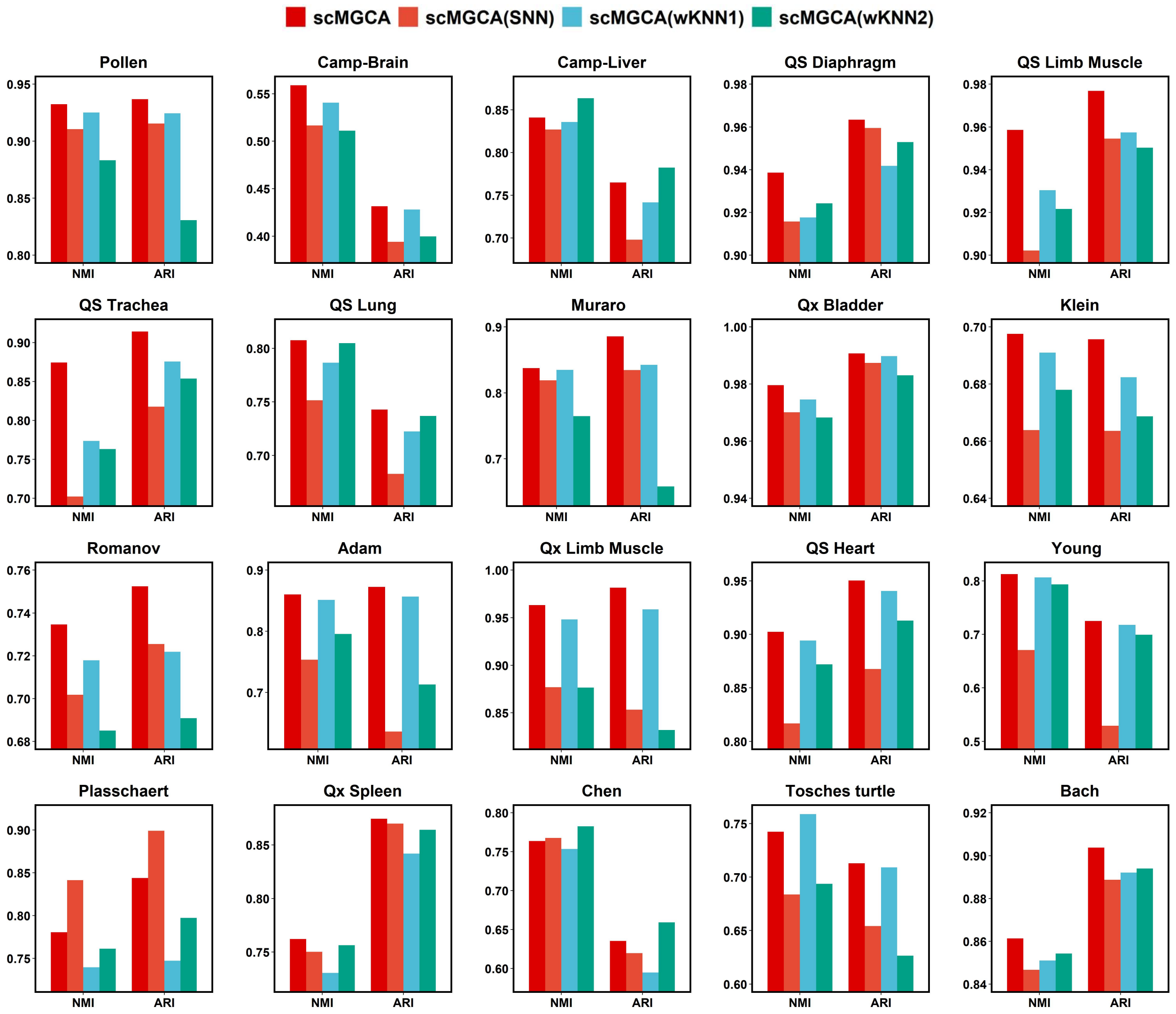

Supplementary Fig. 26. Comparison of NMI and ARI between scMGCA, scMGCA(SNN), scMGCA(wKNN1) and scMGCA(wKNN2) on 20 datasets. Source data are provided as a Source Data file.

Supplementary Note 13: Comparison of the performance of scMGCA with other clustering algorithms in dropout correction

We compare the clustering performance of scMGCA with 10 other deep learning clustering approaches as the single-cell data dropout rate is increased. Following the reference (7), we evaluated different methods by randomly masking the non-zero gene expression information of each cell (replacing with zeros) on single-cell data and then simulating the increase in the probability of data dropout as the masking probability increases. We test the 20 real single-cell datasets where the rate of increase in dropout events is set to [0%, 5%, 10%, 20%, 50%]. The comparison results are shown in Supplementary Fig. 27 and Fig. 28. On the majority of the datasets, it can be observed that scMGCA has a small decrease in NMI and ARI values on most of the datasets and outperforms all other comparison methods. In particular, the clustering performance of scGNN decreases significantly when the dropout events is increased from 20% to 50% on many datasets. These experimental findings confirm that scMGCA is sufficiently stable and robust with promising performance in dropout event correction.

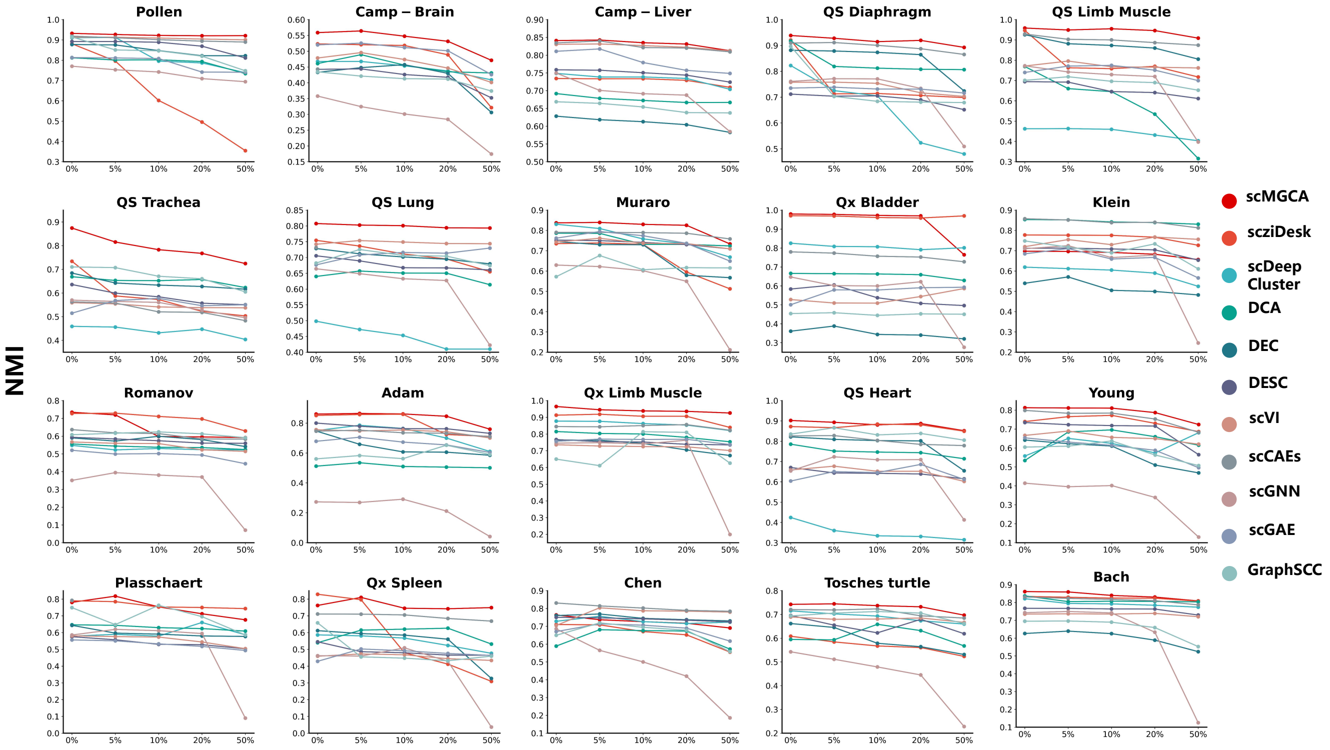

Supplementary Fig. 27. The NMI values of scMGCA with different probabilities of artificial dropout events on 20 real single-cell datasets. Source data are provided as a Source Data file.

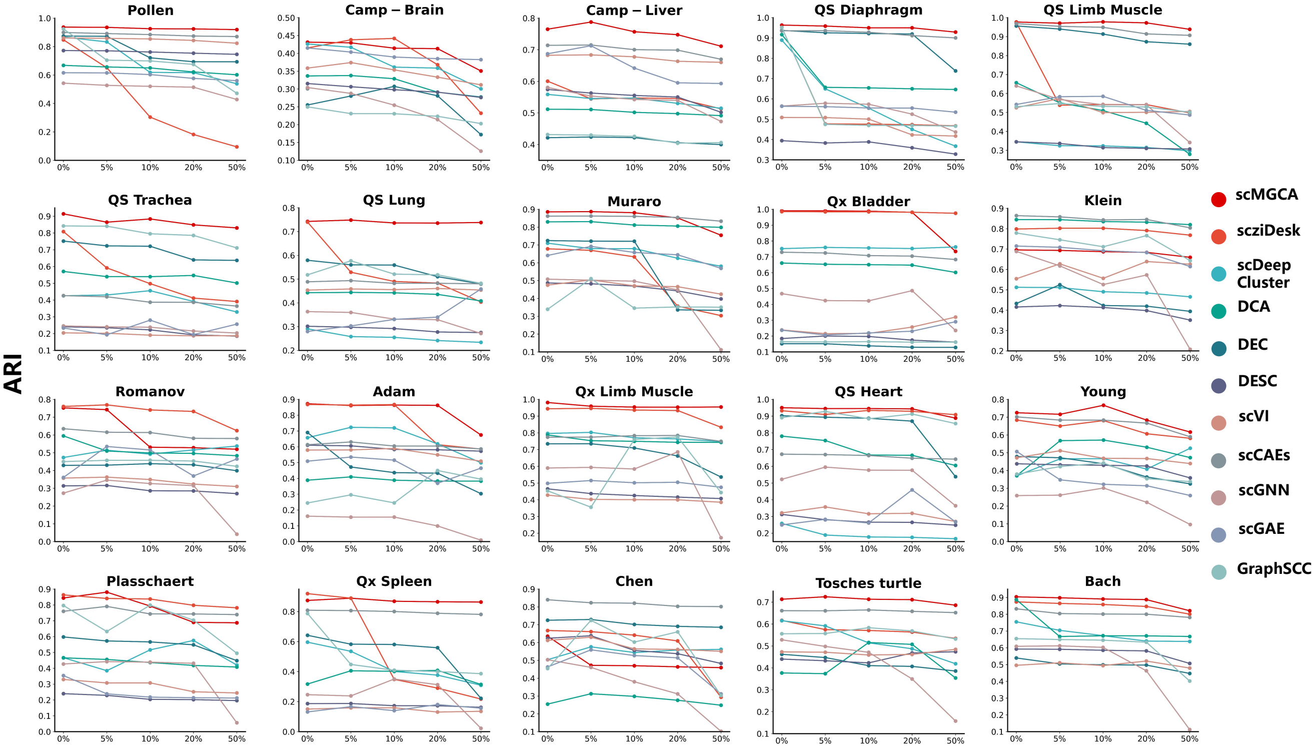

Supplementary Fig. 28. The ARI values of scMGCA with different probabilities of artificial dropout events on 20 real single-cell datasets. Source data are provided as a Source Data file.

Supplementary Note 14: Comparison of scMGCA and other clustering methods on functional genomics studies

We provided a comparison of scMGCA with other clustering algorithms for functional genomics studies. Since our functional interpretation approach is used to explain deep learning models, we adopt six deep clustering algorithms as comparison methods including three non-GNN-based methods (scziDesk, scDeepCluster, and DCA) and three GNN-based algorithms (scGNN, scGAE, and GraphSCC).

In scMGCA, we propose an algorithm based on the standard deviation of the weight matrix in the encoder to uncover the key interpretable genes of the latent representation. To construct a fair comparison, we also applied this algorithm to explain all other compared methods, enabling each method to extract 200 highly expressed genes on 'Qx Limb Muscle'. After that, we visualized the top 10 highly expressed genes in different cell clusters by various deep clustering algorithms, as depicted in Supplementary Fig. 29. It can be observed that scMGCA can clearly find the respective highly expressed genes for each cell cluster without confusion with other cell clusters, while the other clustering methods overlap or fail in clarifying certain cell clusters; for instance, for B cells and skeletal muscle cells, the expression levels of the highly expressed genes identified by scziDesk were not prominent. scDeepCluster, DCA and scGNN found that the top 10 highly expressed genes in some cell clusters were also highly expressed in other cell clusters, without the obvious identification of specific genes that were highly expressed in one cell cluster alone. The gene expression of GraphSCC in B cells and mesenchymal cells is not high. scGAE essentially did not find highly expressed genes in macrophages, skeletal muscle cells, and B cells.

In addition, we further compared the capacity of scMGCA and other clustering methods to uncover differentially expressed genes. For each algorithm, we extracted 200 differentially expressed genes based on their predicted labels by performing the wilcoxon analysis of one group against the other groups. We then performed multiple hypothesis tests using the Benjamini-Hochberg correction to adjust for the correct p values ($pvals\_adj$). Finally, we computed the $pvals\_adj$ of the 200 differential genes of each method as the calling probability of the differential genes while those of the real differentially expressed genes obtained from the the annotated labels in the data are considred as the true measures. We then used the scikit-learn python package to evaluate the area under the ROC curve (AUC) for each method and plotted the ROC curve, as summarized in Supplementary Fig. 30. Overall, scMGCA (AUC = 0.89) is generally superior to other clustering methods.

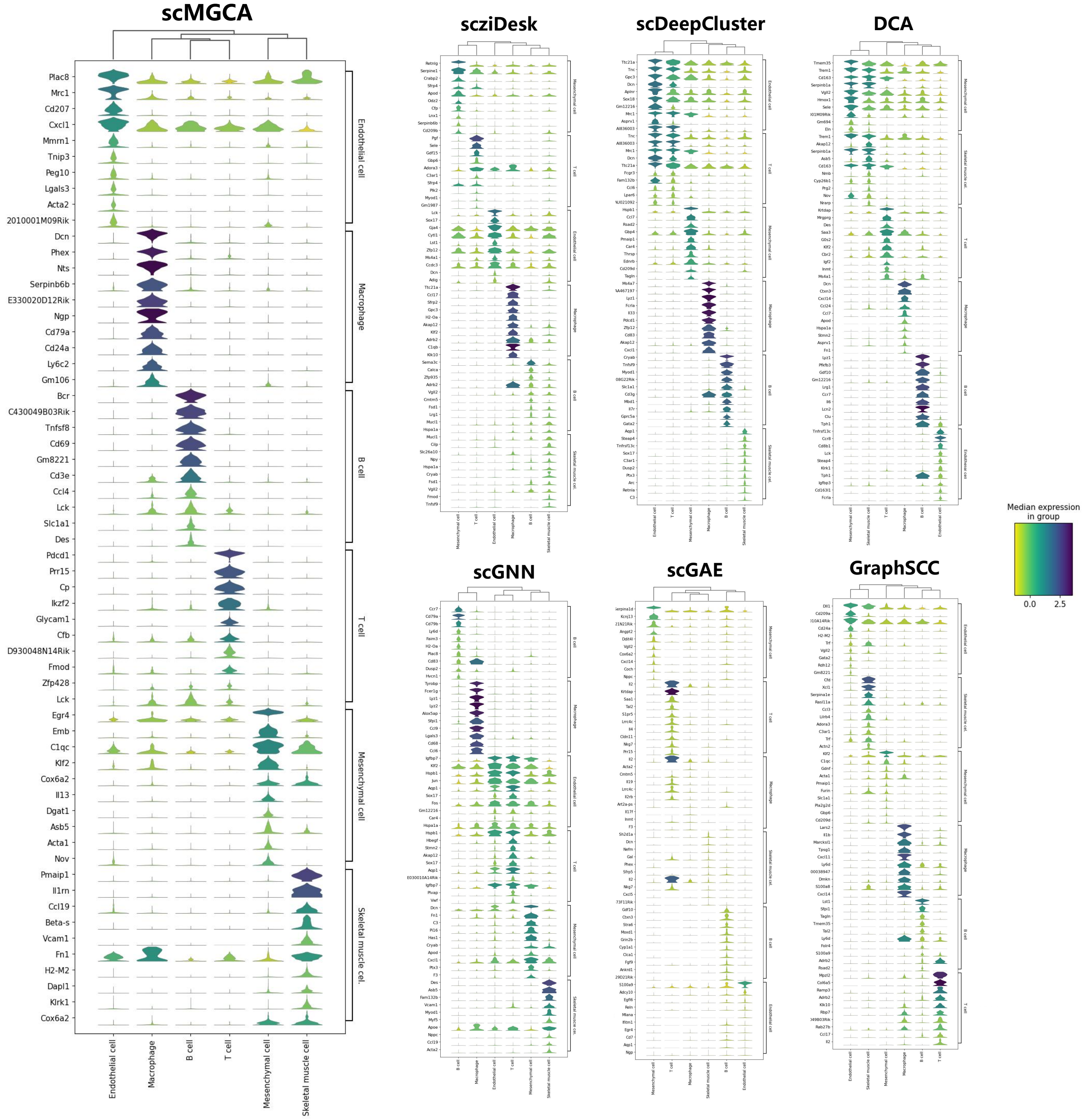

Supplementary Fig. 29. Stacked violin plots of the top 10 genes expressed in each cluster for scMGCA and comparative clustering methods.

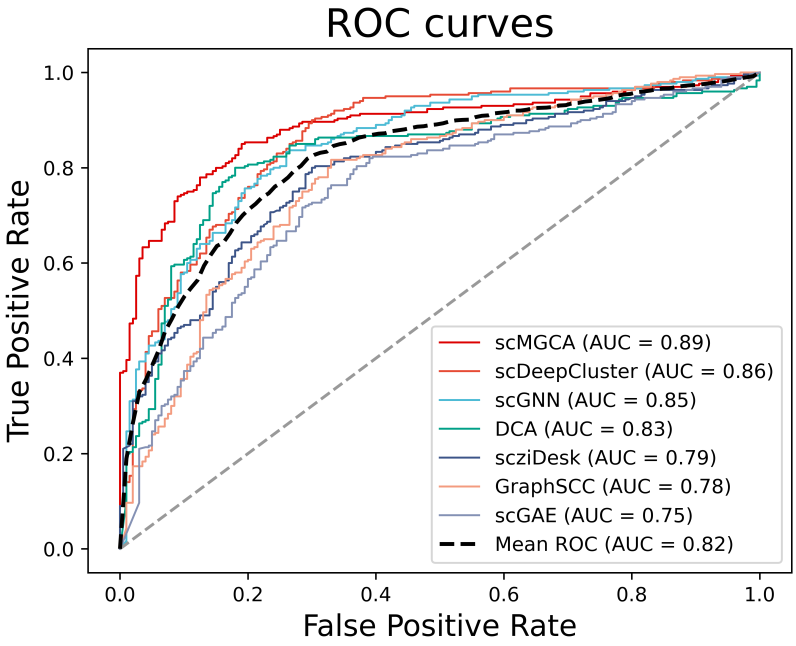

Supplementary Fig. 30. Accuracy measurements of differentially expressed genes by different clustering methods.

Supplementary Note 15: Comparison of scMGCA with SCANPY and Seurat in detecting genomic features

We provided a comparison of scMGCA with SCANPY and Seurat for detecting genomic features. For SCANPY, we clustered the data using the function *scanpy.tl.leiden* with the parameter set to "*resolution* = '0.2', and detected differential genes using the function *scanpy.tl.rank_genes_groups* with the parameter set to " *method*='wilcoxon'". For Seurat, we used the function *FindClusters* to cluster the data, where the parameters were set to "*resolution* = '0.2'", and employed the function *FindAllMarkers* to detect differential genes, where the parameters were set to "*min.pct* = '0.25', *logfc.threshold* = '0.5'".

We used SCANPY and Seurat, respectively, to detect differentially expressed genes with high gene expression levels in each cell cluster, and then compared with the highly expressed genes among the 200 genes extracted by scMGCA. We selected three important marker genes that only scMGCA could detect, and compared the expression of these three marker genes in the three methods using violin plots (Supplementary Fig. 31). The marker genes are Dcn, Pdcd1 and Il1rn, respectively. The Dcn gene provides instructions for making a protein called decorin, which enhances IFN-γ- and LPC-dependent macrophage activation in mice (8). The Pdcd1 correlates with clonal size of follicular T cells and is a known gene that positively correlates with clonal expansion (9). The expression of the Il1rn gene affects body weight and glucose uptake in skeletal muscle cells, providing a hypothesis for a functional link between obesity and insulin resistance (10). It can be clearly seen from Supplementary Fig. 31 that the two genes Pdcd1 and Il1rn were not detected using the SCANPY and Seurat methods. Although the gene Dcn was identified by all three methods, only scMGCA used it as a marker gene of the macrophage cell cluster.

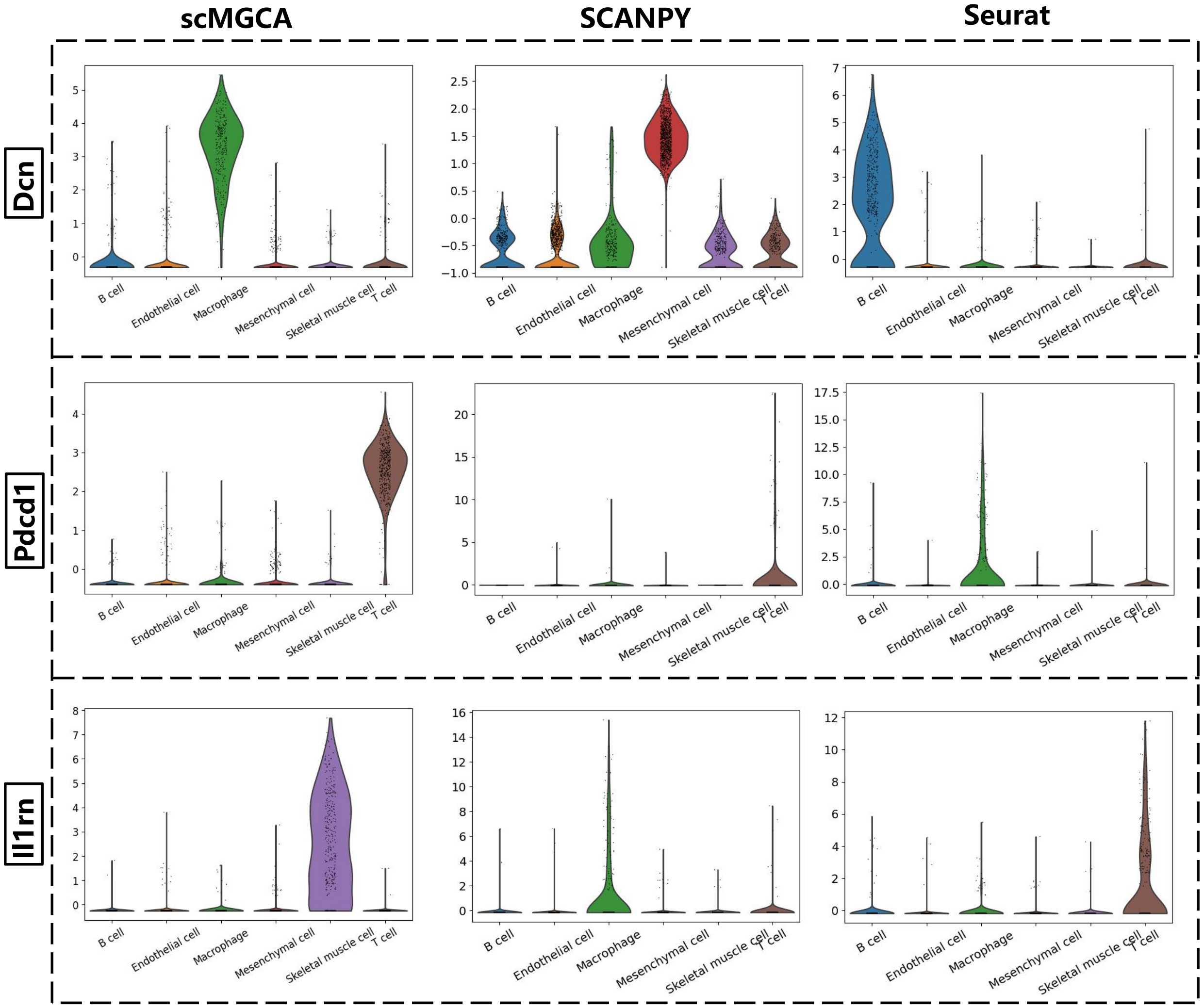

Supplementary Fig. 31. Stacked violin plots of the selected genes expressed in each cluster for scMGCA, SCANPY, and Seurat.

Supplementary Note 16: Cell trajectory inference by scMGCA on time-series datasets

We adopted two time-series datasets (‘Klein’ and hESC dataset) to explain the ability of scMGCA in cell trajectory. The ‘Klein’ dataset (GSE65525) is composed of mouse embryonic stem (ES) cells with time-series information on the ES cell differentiation process (11). Specifically, a total of 2717 cells were profiled at 0 (cell number n = 933), 2 (n = 303), 4 (n = 683) and 7 (n = 798) days after ES cell differentiation. To demonstrate that the interpretable genes selected by scMGCA have the ability to annotate cell trajectories, we compared trajectory inference and pseudo-time analysis of the "Klein" dataset using the original gene expression matrix and the gene expression matrix consisting of 200 genes selected by scMGCA (Supplementary Fig. 32a). From Supplementary Fig. 32a, it can be observed that the raw data does not lead to the trajectory path well and reveals the wrong stage of cell differentiation, whereas the cell trajectories inferred by the interpretable genes picked by scMGCA were closely related to the true stage of cell differentiation and followed the true stage of differentiation accurately: from Day 0 to Day 2, to Day 4, and finally to Day 7.

In addition, we also analyzed a time-course hESC dataset (GSE75748) derived from the differentiation of H1 ESC into definitive endoderm cells (DEC) (12). A total of 758 cells were profiled at 0 (cell number n = 92), 12 (n = 102), 24 (n = 66), 36 (n = 172), 72 (n = 138) and 96 (n = 188) hours after inducing the differentiation from H1 ESCs to DECs. We also compared the trajectory inference and pseudo-time analysis using the original gene expression matrix and the gene expression matrix consisting of 200 genes selected by scMGCA (Supplementary Fig. 32b). As shown in Supplementary Fig. 32b, in contrast to the raw data showing incorrect cell differentiation stages, the interpretable genes picked by scMGCA inferred cell trajectories with the annotated cell differentiation stages and accurately followed the true differentiation stages: from Hour 0 to Hour 12, to Hour 24, to Hour 36, to Hour 72, and finally to Hour 96.

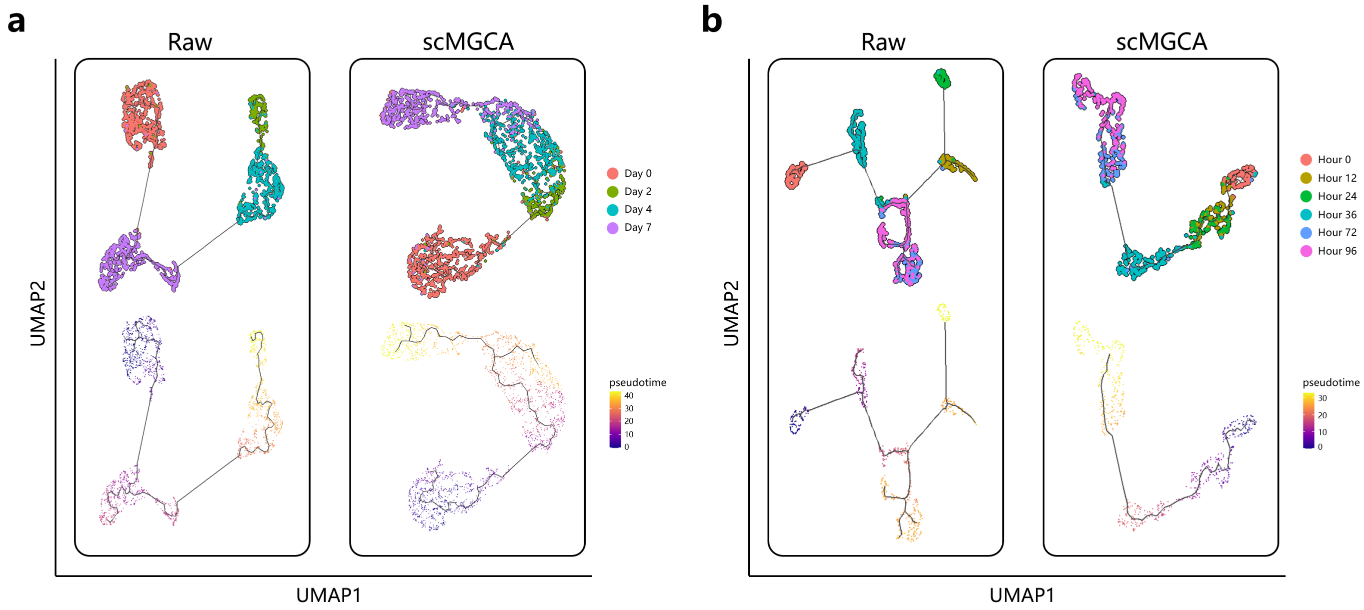

Supplementary Fig. 32 Trajectory inference and pseudotime analysis using the raw gene expression matrix and the gene expression matrix composed of those 200 interpretable genes selected by scMGCA on **a** ‘Klein’ dataset and **b** hESC dataset via Monocle.

Supplementary Note 17: Analysis of the PDAC dataset

The original dataset contains 57,530 cells and 18,008 genes from CRA001160.

Data preprocessing: We used SCANPY to preprocess the dataset: 1. Cells and genes were filtered using "$scanpy.pp.filter\_cells$" with (min_counts=200) and "$scanpy.pp.filter\_genes$" with (min_counts=20), respectively. 2. Calculate the quality control index by "$scanpy.pp.calculate\_qc\_metrics$", and then filter the cell quality according to (n_genes_by_counts < 2500 and pct_counts_mt < 5). 3. Gene expression levels were normalized using a "$scanpy.pp.normalize\_total$" with (target_sum=1e4). 4. The data was log-transformed using"$scanpy.pp.log1p$". 5. The data after normalization used "$scanpy.pp.highly\_variable\_genes$" with (min_mean=0.0125, max_mean=3, min_disp=0.5, n_top_genes = 2000) to select the top 2000 highly variable genes. After the above filtering and data processing, there are 35,942 cells and 2,000 highly variable genes remained.

Parameters setting: The initialization center adopted the Leiden algorithm, and the resolution was set to 0.2. The parameters we used were pretrain_epochs=500, maxiter=300, batch=10000, W_a=0.6. Other parameters were set to default values.

Supplementary Table 23. Marker genes used to characterise unknown cell types

Cell Type	Marker gene
Acinar cell	PRSS1, CTRB1, CTRB2, REG1B
B cell	MS4A1, CD79A, CD79B, CD52
Ductal cell 1	AMBP, CFTR, MMP7
Ductal cell 2	KRT19, KRT7, TSPAN8, SLPI
Endocrine cell	CHGB, CHGA, INS, IAPP
Endothelial cell	CDH5, PLVAP, VWF, CLDN5
Fibroblast	LUM, DCN, COL1A1
Macrophage	AIF1, CD64, CD14, CD68
Stellate cell	RGS5, ACTA2, PDGFRB, ADIRF
T cell	CD3D, CD3E, CD4, CD8

Supplementary Note 18: GSVA enrichment pathway diagram of the result of scMGCA and TCGA dataset

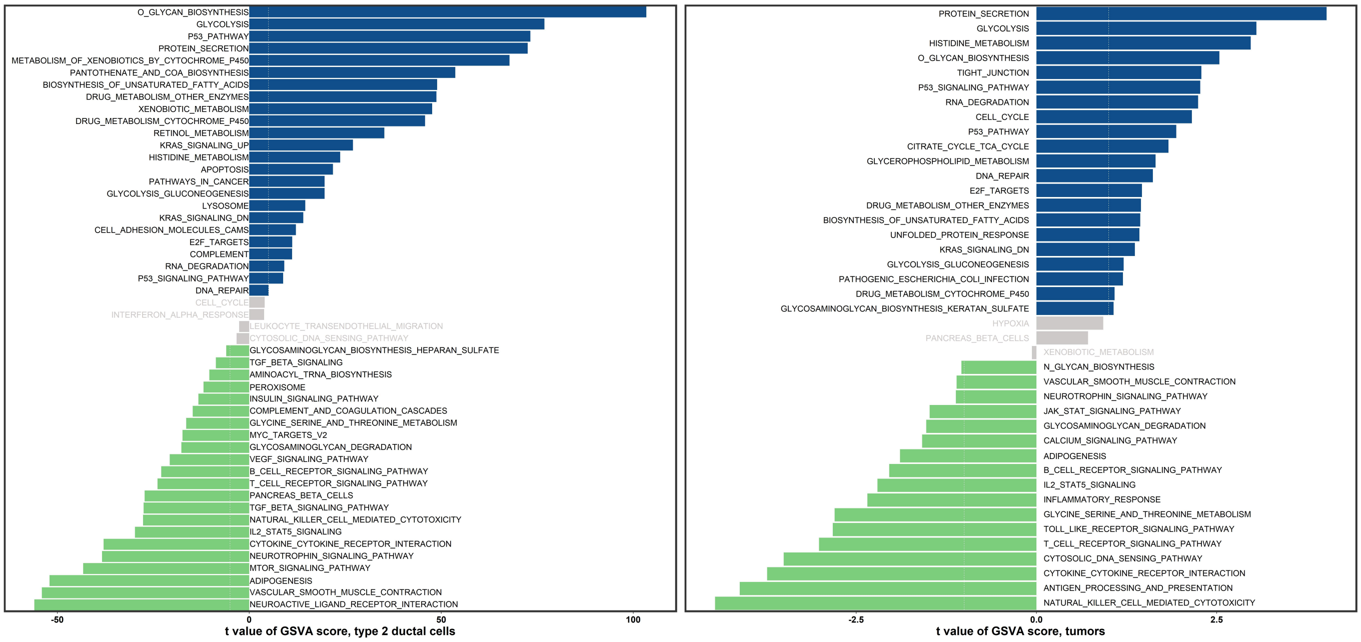

Supplementary Fig. 33. GSVA enrichment pathway diagram of the result of scMGCA (left) and TCGA dataset (right).

Supplementary Note 19: scMGCA can reveal potential relationship between SLE and PDAC

The pathway for systemic lupus erythematosus (SLE) is also enriched in PDAC (Supplementary Fig. 34). For further research, we have extracted the genes enriched in the SLE pathway and employed STRING (13) to construct the PPI network for the selected genes (Supplementary Fig. 35a). Then, we used molecular complex detection (MCODE) (14) in Cytoscape (15) to identify three important modules in the PPI network (Supplementary Fig. 35b). Further, we selected the top three genes in the PPI network through CytoHubba (16), which were also in the module with the highest score; thus, we use these three genes as the hub genes of SLE (Supplementary Fig. 35c). Finally, we studied the differential expression of these three hub genes between tumors and adjacent normal tissues in the TCGA pan-cancer dataset (Supplementary Fig. 36). It can be observed from Supplementary Fig. 36 that the gene expression levels of these three genes on the PAAD data are relatively significant, and the p-values calculated by the Wilcoxon test are all less than 0.01. Therefore, we believe that SLE and PDAC are potentially related.

To verify whether the SLE signaling pathways identified by the single-cell PDAC data are indeed clinically relevant, we also independently adopted the TCGA's database public of invasive pancreatic ductal adenocarcinomas and its variants (PAAD) to validate the insight consistency. Specifically, we performed GSVA enrichment of SLE-related genes on tumor cells from PDAC and 178 tumor samples from TCGA data, respectively, and then compared them (Supplementary Fig. 37). It can be found that the SLE pathway can be enriched in both PDAC and its TCGA data, and both are expressed in tumor cells. In particular, some important signaling pathways in pathogenic process of SLE were also enriched, including coagulation cascade and complement system (17–19). These results demonstrate that an association of SLE with PDAC can be found in large cohort studies. In addition, several studies have shown that SLE has been studied as a risk factor for pancreatic cancer (20, 21), and that SLE may be associated with an increased risk of pancreatic cancer (22, 23). In summary, we have revealed a potential relationship between SLE and PDAC, which has also been demonstrated in clinical data.

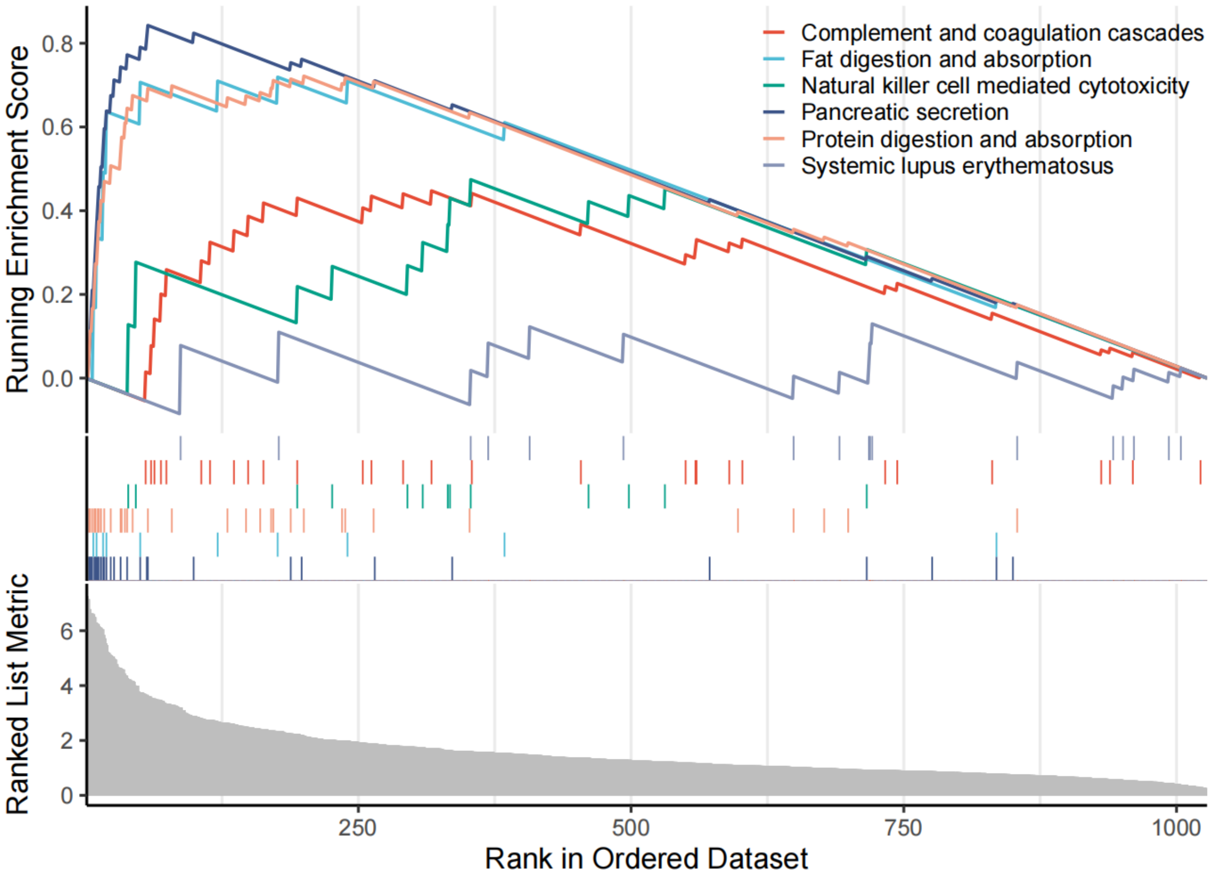

Supplementary Fig. 34. GSEA on KEGG pathways for PDAC.

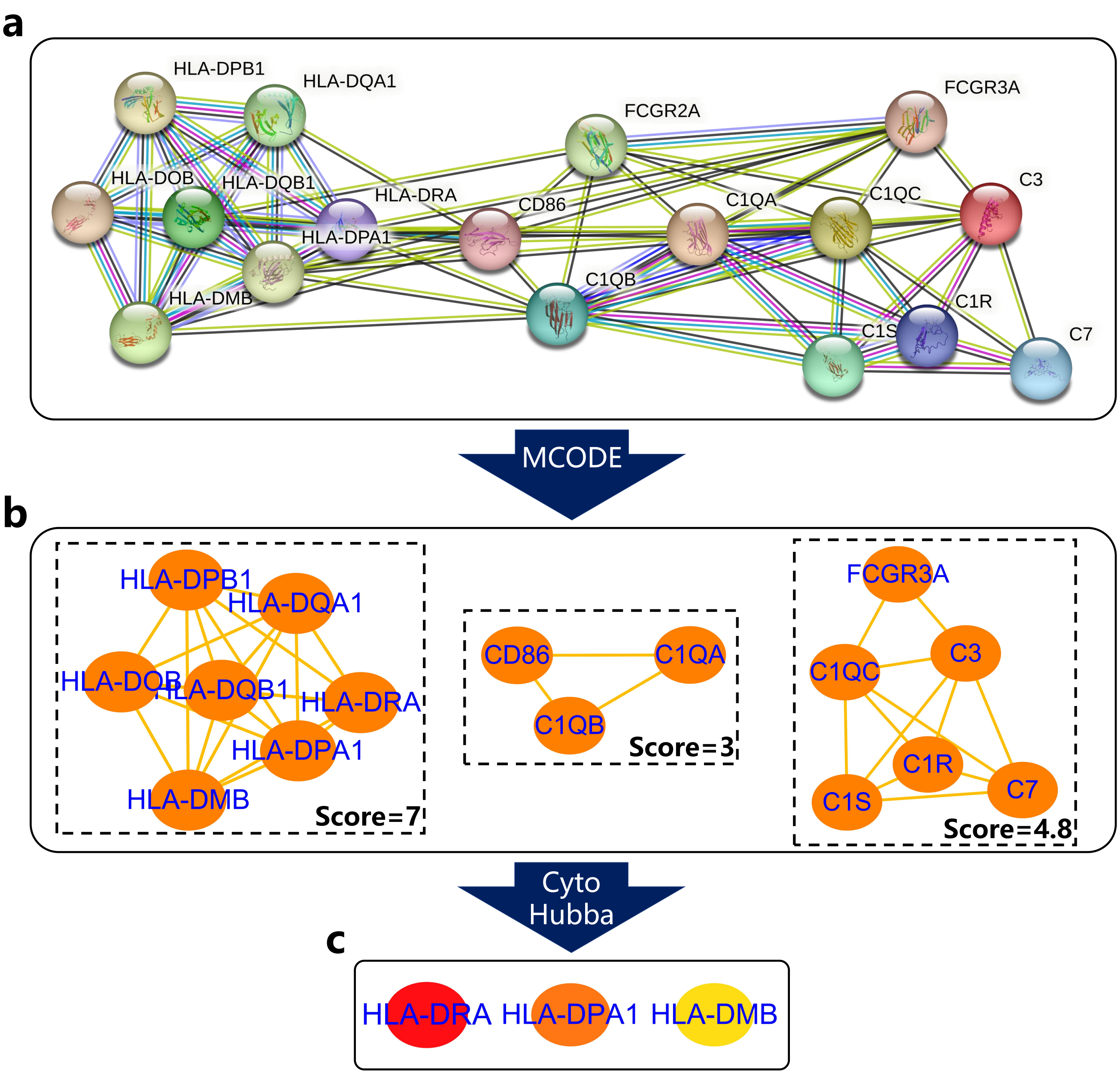

Supplementary Fig. 35. Protein-protein interaction (PPI) network complex and modular analysis of genes enriched in the SLE pathway. **a** PPI network of genes enriched in the SLE pathway. **b** PPI networks in MCODE analysis. **c** Top three hub genes in CytoHubba analysis.

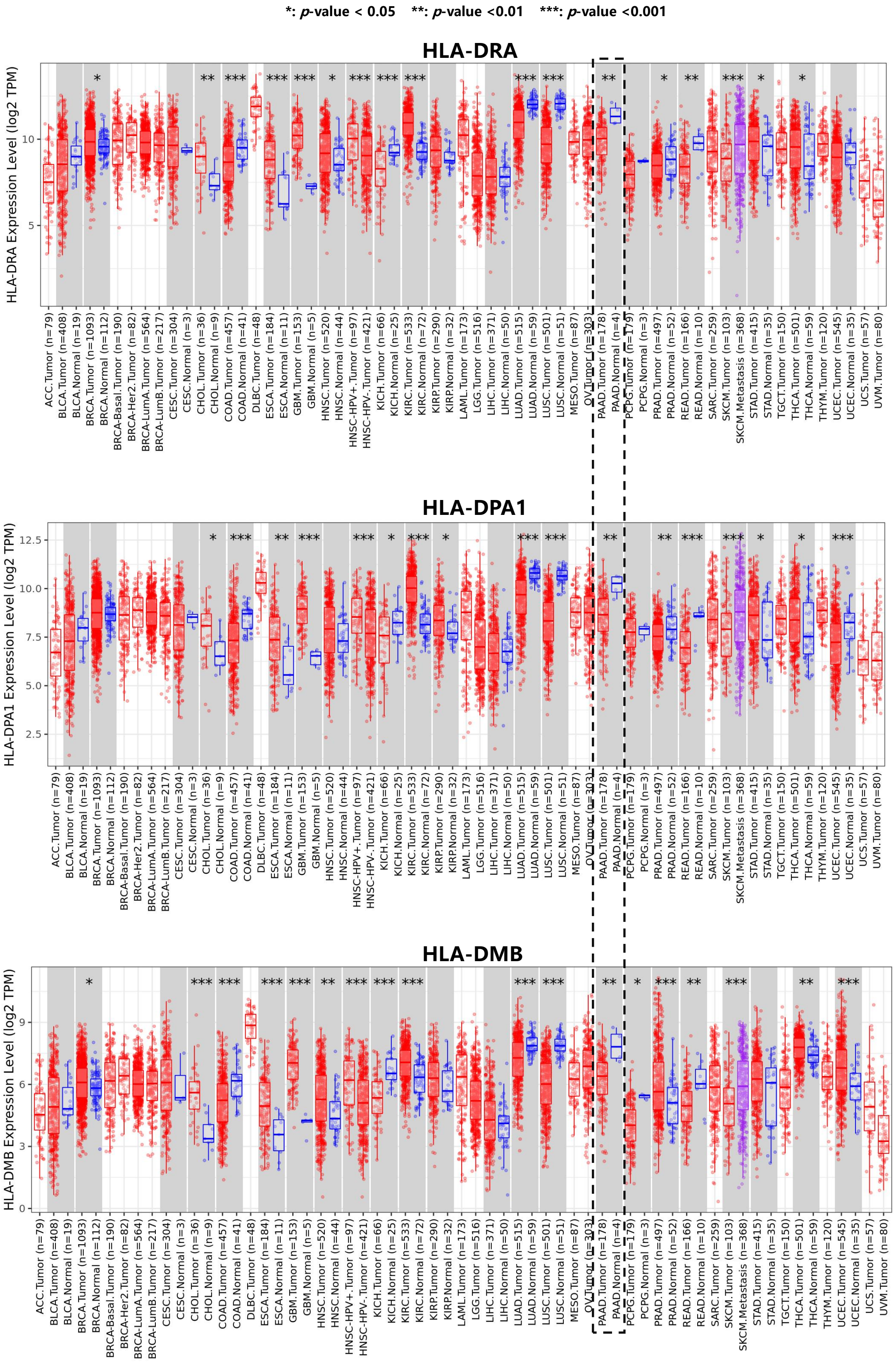

Supplementary Fig. 36. The differential expression of three hub genes between tumors and adjacent normal tissues in the TCGA pan-cancer dataset (center line, median; box limits, upper and lower quartiles; whiskers, 1.5× interquartile range). Significance is determined by two-sided Wilcoxon test (adopt BH to adjust p-values for multiple comparisons), and the p-values are 0.0049, 0.0065, and 0.0067, respectively.

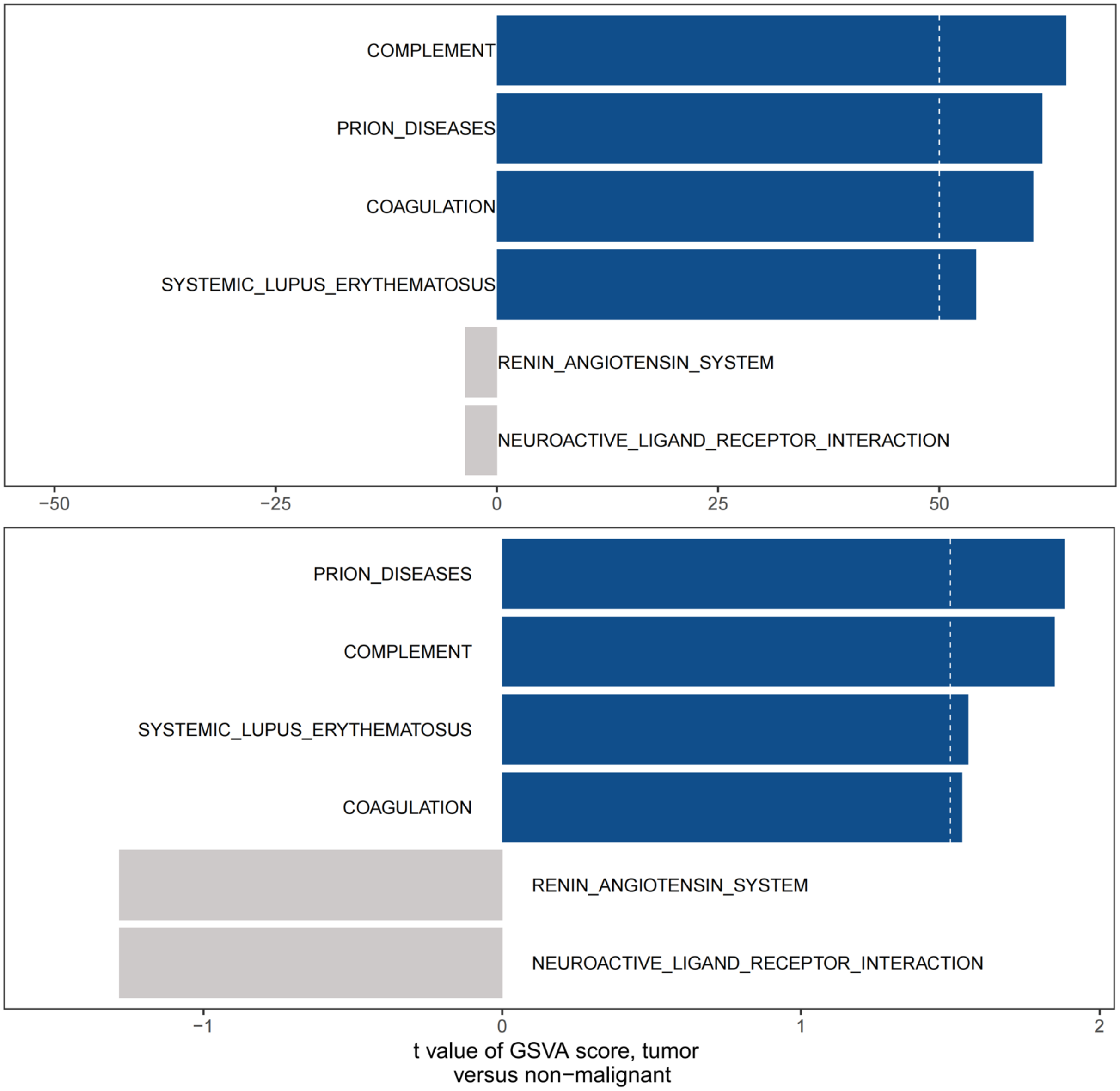

Supplementary Fig. 37. GSVA enrichment pathway diagram of the result of single-cell RNA-seq data (top) and TCGA dataset (down).

Supplementary Note 20: Data description

Supplementary Table 24. Summary of 20 real scRNA-seq datasets across multiple platforms

Dataset	Organ	Cell	Gene	Class	Platform	Reference
Pollen	Tissues	301	21721	11	SMARTer	(24)
Camp-Brain	Brain	734	18927	6	SMARTer	(25)
Camp-Liver	Liver	777	19020	7	SMARTer	(26)
QS Diaphragm	Diaphragm	870	23341	5	Smart-seq2	(27)
QS Limb Muscle	Limb Muscle	1090	23341	6	Smart-seq2	(27)
QS Trachea	Trachea	1350	23341	2	Smart-seq2	(27)
QS Lung	Lung	1676	23341	11	Smart-seq2	(27)
Muraro	Pancreas	2122	19046	9	CEL-seq2	(28)
Qx Bladder	Bladder	2500	23341	4	10x	(27)
Klein	Embryonic Stem Cell	2717	24047	4	inDrop	(11)
Romanov	Hypothalamus	2881	21143	7	SMARTer	(29)
Adam	Kidney	3660	23797	8	Drop-seq	(30)
Qx Limb Muscle	Limb Muscle	3909	23341	6	10x	(27)
QS Heart	Heart	4365	23341	8	Smart-seq2	(27)
Young	Kidney	5685	33658	11	10x	(31)
Plasschaert	Trachea	6977	28205	8	inDrop	(32)
Qx Spleen	Spleen	9522	23341	5	10x	(27)
Chen	Brain	12089	23284	46	Drop-seq	(33)
Tosches turtle	Brain	18664	23500	15	Drop-seq	(34)
Bach	Gammary Gland	23184	19965	8	10x	(35)

References

1. Xiangjie Li, Kui Wang, Yafei Lyu, Huize Pan, Jingxiao Zhang, Dwight Stambolian, Katalin Susztak, Muredach P Reilly, Gang Hu, and Mingyao Li. Deep learning enables accurate clustering with batch effect removal in single-cell rna-seq analysis. *Nature communications*, 11(1):1–14, 2020.
2. Juexin Wang, Anjun Ma, Yuzhou Chang, Jianting Gong, Yuexu Jiang, Ren Qi, Cankun Wang, Hongjun Fu, Qin Ma, and Dong Xu. scgnn is a novel graph neural network framework for single-cell rna-seq analyses. *Nature communications*, 12(1):1–11, 2021.
3. Huazhu Fu, Hang Xu, Kelvin Chong, Mengwei Li, Kok Siong Ang, Hong Kai Lee, Jingjing Ling, Ao Chen, Ling Shao, Longqi Liu, et al. Unsupervised spatially embedded deep representation of spatial transcriptomics. *Biorxiv*, 2021.
4. Zixiang Luo, Chenyu Xu, Zhen Zhang, and Wenfei Jin. scgae: topology-preserving dimensionality reduction for single-cell rna-seq data using graph autoencoder. *bioRxiv*, 2021.
5. Chen Xu and Zhengchang Su. Identification of cell types from single-cell transcriptomes using a novel clustering method. *Bioinformatics*, 31(12):1974–1980, 2015.
6. Ruizhi Xiang, Wencan Wang, Lei Yang, Shiyuan Wang, Chaohan Xu, and Xiaowen Chen. A comparison for dimensionality reduction methods of single-cell rna-seq data. *Frontiers in genetics*, 12: 646936, 2021.
7. Cédric Arisdakessian, Olivier Poirion, Breck Yunits, Xun Zhu, and Lana X Garmire. Deepimpute: an accurate, fast, and scalable deep neural network method to impute single-cell rna-seq data. *Genome biology*, 20(1):1–14, 2019.
8. Mònica Comalada, Marina Cardó, Jordi Xaus, Annabel F Valledor, Jorge Lloberas, Francesc Ventura, and Antonio Celada. Decorin reverses the repressive effect of autocrine-produced tgf-β on mouse macrophage activation. *The Journal of Immunology*, 170(9):4450–4456, 2003.
9. Elliot H Akama-Garren, Theo van den Broek, Lea Simoni, Carlos Castrillon, Cees E van der Poel, and Michael C Carroll. Follicular t cells are clonally and transcriptionally distinct in b cell-driven mouse autoimmune disease. *Nature communications*, 12(1):1–19, 2021.
10. E Somm, P Cettour-Rose, C Asensio, A Charollais, M Klein, C Theander-Carrillo, CE Juge-Aubry, J-M Dayer, MJH Nicklin, P Meda, et al. Interleukin-1 receptor antagonist is upregulated during diet-induced obesity and regulates insulin sensitivity in rodents. *Diabetologia*, 49(2):387–393, 2006.
11. Allon M Klein, Linas Mazutis, Ilke Akartuna, Naren Tallapragada, Adrian Veres, Victor Li, Leonid Peshkin, David A Weitz, and Marc W Kirschner. Droplet barcoding for single-cell transcriptomics applied to embryonic stem cells. *Cell*, 161(5):1187–1201, 2015.
12. Li-Fang Chu, Ning Leng, Jue Zhang, Zhonggang Hou, Daniel Mamott, David T Vereide, Jeea Choi, Christina Kendziorski, Ron Stewart, and James A Thomson. Single-cell rna-seq reveals novel regulators of human embryonic stem cell differentiation to definitive endoderm. *Genome biology*, 17(1):1–20, 2016.
13. Damian Szklarczyk, Annika L Gable, David Lyon, Alexander Junge, Stefan Wyder, Jaime Huerta-Cepas, Milan Simonovic, Nadezhda T Doncheva, John H Morris, Peer Bork, et al. String v11: protein–protein association networks with increased coverage, supporting functional discovery in genome-wide experimental datasets. *Nucleic acids research*, 47(D1):D607–D613, 2019.
14. Gary D Bader and Christopher WV Hogue. An automated method for finding molecular complexes in large protein interaction networks. *BMC bioinformatics*, 4(1):2, 2003.
15. Michael E Smoot, Keiichiro Ono, Johannes Ruscheinski, Peng-Liang Wang, and Trey Ideker. Cytoscape 2.8: new features for data integration and network visualization. *Bioinformatics*, 27(3): 431–432, 2011.
16. Chia-Hao Chin, Shu-Hwa Chen, Hsin-Hung Wu, Chin-Wen Ho, Ming-Tat Ko, and Chung-Yen Lin. cytohubba: identifying hub objects and sub-networks from complex interactome. *BMC systems biology*, 8(4):1–7, 2014.
17. Celine C Berthier, Matthias Kretzler, and Anne Davidson. A systems approach to renal inflammation in sle. *Clinical Immunology*, 185:109–118, 2017.
18. Orthodoxia Nicolaou, Andreas Kousios, Andreas Hadjisavvas, Bernard Lauwerys, Kleitos Sokratous, and Kyriacos Kyriacou. Biomarkers of systemic lupus erythematosus identified using mass spectrometry-based proteomics: a systematic review. *Journal of cellular and molecular medicine*, 21(5):993–1012, 2017.
19. Anupam Guleria, Avadhesh Pratap, Durgesh Dubey, Atul Rawat, Smriti Chaurasia, Edavalath Sukesh, Sanat Phatak, Sajal Ajmani, Umesh Kumar, Chunni Lal Khetrapal, et al. Nmr based serum metabolomics reveals a distinctive signature in patients with lupus nephritis. *Scientific reports*, 6(1):1–11, 2016.
20. Paulina Gomez-Rubio, Janet Piñero, Esther Molina-Montes, Alba Gutiérrez-Sacristán, Mirari Marquez, Marta Rava, Christoph W Michalski, Antoni Farré, Xavier Molero, Matthias Löhr, et al. Pancreatic cancer and autoimmune diseases: An association sustained by computational and epidemiological case–control approaches. *International journal of cancer*, 144(7):1540–1549, 2019.
21. Min Zhang, Yizhou Wang, Yutong Wang, Ye Bai, and Dongqing Gu. Association between systemic lupus erythematosus and cancer morbidity and mortality: Findings from cohort studies. *Frontiers in oncology*, page 1710, 2022.
22. Min-Seok Seo, Jina Yeo, In Cheol Hwang, and Jae-Yong Shim. Risk of pancreatic cancer in patients with systemic lupus erythematosus: a meta-analysis. *Clinical rheumatology*, 38(11):3109–3116, 2019.
23. Margaret A Tempero, Mokenge P Malafa, Mahmoud Al-Hawary, Stephen W Behrman, Al B Benson, Dana B Cardin, E Gabriela Chiorean, Vincent Chung, Brian Czito, Marco Del Chiaro, et al. Pancreatic adenocarcinoma, version 2.2021, nccn clinical practice guidelines in oncology. *Journal of the National Comprehensive Cancer Network*, 19(4):439–457, 2021.
24. Alex A Pollen, Tomasz J Nowakowski, Joe Shuga, Xiaohui Wang, Anne A Leyrat, Jan H Lui, Nianzhen Li, Lukasz Szpankowski, Brian Fowler, Peilin Chen, et al. Low-coverage single-cell mrna sequencing reveals cellular heterogeneity and activated signaling pathways in developing cerebral cortex. *Nature biotechnology*, 32(10):1053–1058, 2014.
25. J Gray Camp, Farhath Badsha, Marta Florio, Sabina Kanton, Tobias Gerber, Michaela Wilsch-Bräuninger, Eric Lewitus, Alex Sykes, Wulf Hevers, Madeline Lancaster, et al. Human cerebral organoids recapitulate gene expression programs of fetal neocortex development. *Proceedings of the National Academy of Sciences*, 112(51):15672–15677, 2015.
26. J Gray Camp, Keisuke Sekine, Tobias Gerber, Henry Loeffler-Wirth, Hans Binder, Malgorzata Gac, Sabina Kanton, Jorge Kageyama, Georg Damm, Daniel Seehofer, et al. Multilineage communication regulates human liver bud development from pluripotency. *Nature*, 546(7659):533–538, 2017.
27. Nicholas Schaum, Jim Karkanias, Norma F Neff, Andrew P May, Stephen R Quake, Tony Wyss-Coray, Spyros Darmanis, Joshua Batson, Olga Botvinnik, Michelle B Chen, et al. Single-cell transcriptomics of 20 mouse organs creates a tabula muris: The tabula muris consortium. *Nature*, 562(7727):367, 2018.
28. Mauro J Muraro, Gitanjali Dharmadhikari, Dominic Grün, Nathalie Groen, Tim Dielen, Erik Jansen, Leon Van Gurp, Marten A Engelse, Francoise Carlotti, Eelco Jp De Koning, et al. A single-cell transcriptome atlas of the human pancreas. *Cell systems*, 3(4):385–394, 2016.
29. Roman A Romanov, Amit Zeisel, Joanne Bakker, Fatima Girach, Arash Hellysaz, Raju Tomer, Alan Alpar, Jan Mulder, Frederic Clotman, Erik Keimpema, et al. Molecular interrogation of hypothalamic organization reveals distinct dopamine neuronal subtypes. *Nature neuroscience*, 20(2):176–188, 2017.
30. Mike Adam, Andrew S Potter, S Steven Potter, et al. Psychrophilic proteases dramatically reduce single-cell rna-seq artifacts: a molecular atlas of kidney development. *Development*, 144(19):

3625–3632, 2017.

31. Matthew D Young, Thomas J Mitchell, Felipe A Vieira Braga, Maxine GB Tran, Benjamin J Stewart, John R Ferdinand, Grace Collord, Rachel A Botting, Dorin-Mirel Popescu, Kevin W Loudon, et al. Single-cell transcriptomes from human kidneys reveal the cellular identity of renal tumors. *Science*, 361(6402):594–599, 2018.
32. Lindsey W Plasschaert, Rapolas Žilionis, Rayman Choo-Wing, Virginia Savova, Judith Knehr, Guglielmo Roma, Allon M Klein, and Aron B Jaffe. A single-cell atlas of the airway epithelium reveals the cftr-rich pulmonary ionocyte. *Nature*, 560(7718):377–381, 2018.
33. Renchao Chen, Xiaoji Wu, Lan Jiang, and Yi Zhang. Single-cell rna-seq reveals hypothalamic cell diversity. *Cell reports*, 18(13):3227–3241, 2017.
34. Maria Antonietta Tosches, Tracy M Yamawaki, Robert K Naumann, Ariel A Jacobi, Georgi Tushev, and Gilles Laurent. Evolution of pallium, hippocampus, and cortical cell types revealed by single-cell transcriptomics in reptiles. *Science*, 360(6391):881–888, 2018.
35. Karsten Bach, Sara Pensa, Marta Grzelak, James Hadfield, David J Adams, John C Marioni, and Walid T Khaled. Differentiation dynamics of mammary epithelial cells revealed by single-cell rna sequencing. *Nature communications*, 8(1):1–11, 2017.
