# Supplementary material for: Topological identification and interpretation for single-cell gene regulation elucidation across multiple platforms using scMGCA
Source: Nat Commun. 2023 Jan 25;14:400. doi: 10.1038/s41467-023-36134-7 (PMC9877026; doi:10.1038/s41467-023-36134-7)
Supplement: Supplementary file 3 — Reporting Summary [file 41467_2023_36134_MOESM3_ESM.pdf]

nature portfolio

Corresponding author(s): Xiangtao Li, Ka-Chun Wong

Last updated by author(s): 2022-Dec-23

Reporting Summary

Nature Portfolio wishes to improve the reproducibility of the work that we publish. This form provides structure for consistency and transparency in reporting. For further information on Nature Portfolio policies, see our Editorial Policies and the Editorial Policy Checklist.

Statistics

For all statistical analyses, confirm that the following items are present in the figure legend, table legend, main text, or Methods section.

n/a	Confirmed	
[ ]	[x]	The exact sample size (*n*) for each experimental group/condition, given as a discrete number and unit of measurement
[x]	[ ]	A statement on whether measurements were taken from distinct samples or whether the same sample was measured repeatedly
[ ]	[x]	The statistical test(s) used AND whether they are one- or two-sided<br>*Only common tests should be described solely by name; describe more complex techniques in the Methods section.*
[x]	[ ]	A description of all covariates tested
[x]	[ ]	A description of any assumptions or corrections, such as tests of normality and adjustment for multiple comparisons
[ ]	[x]	A full description of the statistical parameters including central tendency (e.g. means) or other basic estimates (e.g. regression coefficient) AND variation (e.g. standard deviation) or associated estimates of uncertainty (e.g. confidence intervals)
[ ]	[x]	For null hypothesis testing, the test statistic (e.g. *F*, *t*, *r*) with confidence intervals, effect sizes, degrees of freedom and *P* value noted<br>*Give P values as exact values whenever suitable.*
[x]	[ ]	For Bayesian analysis, information on the choice of priors and Markov chain Monte Carlo settings
[x]	[ ]	For hierarchical and complex designs, identification of the appropriate level for tests and full reporting of outcomes
[ ]	[x]	Estimates of effect sizes (e.g. Cohen's *d*, Pearson's *r*), indicating how they were calculated

Our web collection on statistics for biologists contains articles on many of the points above.

Software and code

Policy information about availability of computer code

Data collection	No software was used for data collection.
Data analysis	We used R package ClusterProfile (v3.18.1), Monocle3 (v1.2.9), Seurat (v4.0.0), RcisTarget (v1.10.0), GSVA (v1.38.2), and CellChat (v1.1.3); Python package SCANPY (v1.6.0); STRING (v11.5), Cytoscape (v3.9.0), and Timer (v2.0) for data analysis. Source code, documentation, and instruction for scMGCA package (v1.0.7) is available at GitHub repository: https://github.com/Philyzh8/scMGCA and scMGCA is also released as a python package at: https://pypi.org/project/scMGCA.

For manuscripts utilizing custom algorithms or software that are central to the research but not yet described in published literature, software must be made available to editors and reviewers. We strongly encourage code deposition in a community repository (e.g. GitHub). See the Nature Portfolio guidelines for submitting code & software for further information.

Data

Policy information about availability of data

All manuscripts must include a data availability statement. This statement should provide the following information, where applicable:

- Accession codes, unique identifiers, or web links for publicly available datasets
- A description of any restrictions on data availability
- For clinical datasets or third party data, please ensure that the statement adheres to our policy

We collected 20 real and public scRNA-seq datasets across multiple platforms. These datasets come from different organs in humans and mouse, such as the brain, pancreas, trachea, and other. These datasets include `Pollen' (SRP041736), `Camp-Brain' (GSE75140), `Camp-Liver' (GSE81252), `Muraro' (GSE85241), `Klein' (GSE65525), `Romanov' (GSE74672), `Adam' (GSE94333), `Young' (EGAS00001002171), `Plasschaert' (GSE102580), `Chen' (GSE87544), `Tosches turtle' (PRJNA408230), `Bach' (GSE106273), and so on. The remaining `QS' and `Qx' datasets are derived from mouse scRNA-seq data generated by Smart-seq2

sequencing and 10x Genomics sequencing, respectively, in the Stanford University study. The details of the 20 scRNA-seq datasets including the number of cells, number of cell types, and organs are tabulated in Supplementary Table 24, and can be downloaded from https://github.com/Philyzh8/scMGCA and https://hemberg-lab.github.io/scRNA.seq.datasets. The dataset `Tabula Muris' (GSE109774) can be downloaded from https://doi.org/10.6084/m9.figshare.5968960.v3. The 1.3 million mouse brain cells dataset contains 1,306,127 cells and 27,998 genes, downloaded from the 10x Genomics website: https://support.10xgenomics.com/single-cell-gene-expression/datasets/1.3.0/1M_neurons. The human pancreatic islet datasets are generated from four different scRNA-seq protocols, including CEL-seq (GSE81076), CEL-seq2 (GSE85241), Fluidigm C1 (GSE86469), and Smart-seq2 (E-MTAB-5061). The hESC dataset (GSE75748) can be downloaded from https://www.ncbi.nlm.nih.gov/geo/query/acc.cgi?acc=GSE75748. The accession number for the PDAC data reported in this paper is GSA: CRA001160. These data have been deposited in the Genome Sequence Archive under project PRJCA001063. The PAAD data comes from TCGA and can be downloaded from https://gdc-hub.s3.us-east-1.amazonaws.com/download/TCGA-PAAD.htseq_counts.tsv.gz. The MSigDB databases for gene set analysis can be downloaded from https://www.gsea-msigdb.org/gsea/msigdb. These datasets have been deposited in the Zenodo database (https://doi.org/10.5281/zenodo.7475687). Source data are provided with this paper.

Field-specific reporting

Please select the one below that is the best fit for your research. If you are not sure, read the appropriate sections before making your selection.

- [x] Life sciences
- [ ] Behavioural & social sciences
- [ ] Ecological, evolutionary & environmental sciences

For a reference copy of the document with all sections, see nature.com/documents/nr-reporting-summary-flat.pdf

Life sciences study design

All studies must disclose on these points even when the disclosure is negative.

Sample size	Sample data were obtained from public repositories. Sample size was not predetermined and is the maximum number of samples available for each datasets.
Data exclusions	No data were excluded from the analysis.
Replication	The experiments were performed three times. All attempts at replication were successful.
Randomization	Not applicable - no experimental groups were assigned.
Blinding	No.

Reporting for specific materials, systems and methods

We require information from authors about some types of materials, experimental systems and methods used in many studies. Here, indicate whether each material, system or method listed is relevant to your study. If you are not sure if a list item applies to your research, read the appropriate section before selecting a response.

Materials & experimental systems

n/a	Involved in the study	
x		Antibodies
x		Eukaryotic cell lines
x		Palaeontology and archaeology
x		Animals and other organisms
x		Human research participants
x		Clinical data
x		Dual use research of concern

Methods

n/a	Involved in the study	
x		ChIP-seq
x		Flow cytometry
x		MRI-based neuroimaging
